# In-vivo T-cell depleted reduced-intensity conditioned allogeneic haematopoietic stem-cell transplantation for patients with acute lymphoblastic leukaemia in first remission: results from the prospective, single-arm evaluation of the UKALL14 trial

**DOI:** 10.1016/S2352-3026(22)00036-9

**Published:** 2022-03-28

**Authors:** David I Marks, Laura Clifton-Hadley, Mhairi Copland, Jiaull Hussain, Tobias F Menne, Andrew McMillan, Anthony V Moorman, Nicholas Morley, Dina Okasha, Bela Patel, Pip Patrick, Michael N Potter, Clare J Rowntree, Amy A Kirkwood, Adele K Fielding

**Affiliations:** a Adult BMT Unit, Bristol Haematology and Oncology Unit, University Hospitals Bristol NHS Trust, Bristol, UK; b Cancer Research UK and UCL Cancer Trial Centre, UCL Cancer Institute, University College London, London, UK; c Paul O'Gorman Leukaemia Research Centre, College of Medical Veterinary and Life Sciences, University of Glasgow, Glasgow, UK; d Department of Haematology, Newcastle Upon Tyne Hospitals NHS Foundation Trust, Newcastle upon Tyne, UK; e Centre for Clinical Haematology, Nottingham City Hospital, Nottingham, UK; f Translational and Clinical Research Institute, Newcastle University, Newcastle-upon-Tyne, UK; g Department of Haematology, Sheffield Teaching Hospitals NHS Trust, Sheffield, UK; h Cancer Institute, University College London, London, UK; i Barts Cancer Institute, The London School of Medicine, Queen Mary University of London, London, UK; j Department of Haematology, Royal Marsden Hospital, Sutton, UK; k Cardiff and Vale University Health Board, Cardiff, UK

## Abstract

**Background:**

The outcome of chemotherapy in patients older than 40 years with acute lymphoblastic leukaemia is poor and myeloablative allogeneic haematopoietic stem-cell transplantation (HSCT) has a high transplant-related mortality (TRM) in this age cohort. The aim of this study was to assess the activity and safety of reduced-intensity conditioned allogeneic HSCT in this patient population.

**Methods:**

This was a single-arm, prospective study within the UKALL14 trial done in 46 centres in the UK, which recruited patients to the transplantation substudy. Participants in UKALL14 had B-cell or T-cell acute lymphoblastic leukaemia, were aged 25–65 years (*BCR-ABL1*-negative) or 18–65 years (*BCR-ABL1*-positive), and for this subcohort had a fit, matched sibling donor or an 8 out of 8 allelic matched unrelated donor (HLA-A, HLA-B, HLA-C, and HLA-DR). On June 20, 2014, the protocol was amended to allow 7 out of 8 matched unrelated donors if the patient had high risk cytogenetics or was minimal residual disease (MRD)-positive after the second induction course. Patients were given fludarabine, melphalan, and alemtuzumab (FMA; intravenous fludarabine 30 mg/m^2^ on days –6 to –2, melphalan 140 mg/m^2^ on day –2, and alemtuzumab 30 mg on day –1 [sibling donor] and days –2 and –1 [unrelated donor]) before allogeneic HSCT (aged ≥41 years patient pathway). Donor lymphocyte infusions were given from 6 months for mixed chimerism or MRD. The primary endpoint was event-free survival and secondary and transplantation-specific endpoints included overall survival, relapse incidence, TRM, and acute and chronic graft-versus-host disease (GVHD). This study is registered with ClinicalTrials.gov, NCT01085617.

**Findings:**

From Feb 22, 2011, to July 26, 2018, 249 patients (236 aged ≥41 years and 13 younger than 41 years) considered unfit for a myeloablative allograft received an FMA reduced-intensity conditioned HSCT. 138 (55%) patients were male and 111 (45%) were female. 88 (35%) participants received transplantations from a sibling donor and 160 (64%) received transplantations from unrelated donors. 211 (85%) participants had B-precursor acute lymphoblastic leukaemia. High-risk cytogenetics were present in 43 (22%) and another 63 (25%) participants were *BCR-ABL1*-positive. At median follow-up of 49 months (IQR 36–70), 4-year event-free survival was 46·8% (95% CI 40·1–53·2) and 4-year overall survival was 54·8% (48·0–61·2). 4-year cumulative incidence of relapse was 33·6% (27·9–40·2) and 4-year TRM was 19·6% (15·1–25·3). 27 (56%) of 48 patients with TRM had infection as the named cause of death. Seven (15%) of 48 patients had fungal infections, 13 (27%) patients had bacterial infections (six gram-negative), and 11 (23%) had viral infections (three cytomegalovirus and two Epstein-Barr virus). Acute GVHD grade 2–4 occurred in 29 (12%) of 247 patients and grade 3–4 occurred in 12 (5%) patients. Chronic GVHD incidence was 84 (37%) of 228 patients (50 [22%] had extensive chronic GVHD). 49 (30%) of 162 patients had detectable end-of-induction MRD, which portended worse outcomes with event-free survival (HR 2·40 [95% CI 1·46–3·93]) and time-to-relapse (HR 2·41 [1·29–4·48]).

**Interpretation:**

FMA reduced-intensity conditioned allogeneic HSCT in older patients with acute lymphoblastic leukaemia in first complete remission provided good disease control with moderate GVHD, resulting in better-than-expected event-free survival and overall survival in this high-risk population. Strategies to reduce infection-related TRM will further improve outcomes.

**Funding:**

Cancer Research UK.

**Research in context**

**Evidence before this study**

Survival in adults with acute lymphoblastic leukaemia decreases with advancing age. Allogeneic haematopoietic stem-cell transplantation (HSCT) is a widely used therapy when the chance of cure with chemotherapy is low, with a range of studies and meta-analyses from the UK, Europe, and the USA showing benefit. However, full-intensity myeloablative-conditioned allogenic HSCT has a high transplant-related mortality (TRM), with some studies reporting more than 30% TRM in older patients, negating its powerful antileukaemic effect. In October, 2021, we searched PubMed for primary research and reviews published since 2000 using the keywords “ALL”, “adults”, “reduced intensity”, or “non-myeloablative conditioning”; there were no language restrictions. We found a retrospective, single institution, single-arm study from the USA using T-replete, reduced-intensity conditioned allogeneic HSCT with fludarabine and melphalan, which showed good leukaemic control with lower TRM but substantial morbidity due to graft-versus-host disease (GVHD), a complication poorly tolerated by older patients. We also identified retrospective registry-based studies comparing different reduced-intensity conditioning regimens. None of these studies have the strengths and applicability of a prospective study of reduced-intensity conditioned allogeneic HSCT embedded within a national acute lymphoblastic leukaemia trial. We hypothesised that GVHD would be less frequent and less severe after adding alemtuzumab, a monoclonal antibody against CD52 used as in-vivo T-cell depletion. After feasibility enquiries made it clear that random assignment between allogeneic HSCT and chemotherapy did not have equipoise, we designed a single-arm, prospective study within the umbrella UK National Cancer Research Institute adult acute lymphoblastic leukaemia trial, UKALL14, in which we aimed to determine event-free survival after reduced-intensity conditioning with fludarabine, melphalan, and alemtuzumab (FMA) in all patients older than 40 years of age with a suitable matched sibling or unrelated donor.

**Added value of this study**

To our knowledge, this 249-patient prospective study is the largest study of reduced-intensity conditioned allogeneic HSCT and demonstrates excellent 4-year event-free survival and overall survival, with low rates of GVHD and reduced TRM, in patients with high-risk acute lymphoblastic leukaemia. Outcomes in this high-risk group exceeded those that would have been expected with chemotherapy alone. The presence of minimal residual disease before allogeneic HSCT was the major risk factor for relapse. This study demonstrates feasibility and tolerability in this patient subset, within a large, prospective, national acute lymphoblastic leukaemia trial.

**Implications of all the available evidence**

This study provides evidence that reduced-intensity conditioned allogeneic HSCT is an appropriate option for older patients with acute lymphoblastic leukaemia with sibling or unrelated donors. In-vivo T-cell depletion with alemtuzumab moderates GVHD. Reducing relapse is a principal goal of the follow-on ALL-RIC study (ISRCTN 99927695), which compares FMA with low dose, total body irradiation-based conditioning.

## Introduction

The results of treating adults with acute lymphoblastic leukaemia are unsatisfactory. In UKALL12,^1^ the largest prospective adult acute lymphoblastic leukaemia trial to date, 5-year overall survival was 39% in 1929 patients aged 18–65 years. Older age was associated with worse outcomes, with 5-year survival of just 23% in those aged 40–49 years and 15% in those aged 50–64 years.^2^, ^3^ Within UKALL12, a donor versus no donor comparison showed that myeloablative sibling allogeneic haematopoietic stem-cell transplantation (HSCT) offered superior protection against relapse in younger patients compared with chemotherapy.^1^ However, there was no overall survival advantage in patients older than 35 years due to a 36% 2-year transplant-related mortality (TRM) ^1^

The UKALL14 trial recruited patients with B-cell and T-cell acute lymphoblastic leukaemia, aged 25–65 years, and included induction randomisations to add rituximab to standard of care for patients with B-cell disease, and to add nelarabine to standard of care for patients with T-cell disease. As post-consolidation therapy for patients older than 40 years, we aimed to retain the antileukaemic potency of allografting while reducing the TRM by using a novel strategy of reduced-intensity conditioned allogeneic HSCT with intensive post-transplantation monitoring, accompanied by donor lymphocyte administration for those with T-cell mixed chimerism or persistent minimal residual disease (MRD).

We selected a single conditioning regimen, FMA (fludarabine, melphalan, and alemtuzumab), which is widely used in the UK and is associated with a low TRM, good results in myeloid malignancies,^4^ and known antileukaemic potency in acute lymphoblastic leukaemia.^5^ The substantial incidence of T-cell mixed chimerism after FMA conditioning, which is linked to relapse in other diseases, informed the plan to administer donor lymphocyte infusion at set doses and time intervals. Because well matched (ie, 8 of 8 allelic matched), unrelated donor allogeneic HSCT results in similar survival to that seen with sibling donors, this strategy was expected to be applicable to most eligible patients.^6^, ^7^

Our aims were to assess event-free survival and relapse rates, to evaluate the role of pretransplantation and post-transplantation MRD, and to document the incidence of mixed chimerism and its response to protocolised donor lymphocyte infusion in the largest prospective trial of FMA reduced-intensity conditioned allogeneic HSCT. UKALL14 aimed to assess the effect of introducing non-myeloablative conditioning in transplantation-eligible patients aged 41 years and older: the final results are presented here.

## Methods

### Study design and participants

This was a single-arm, prospective study within the UKALL14 trial open in 46 acute haematology centres in the UK, which recruited patients to this transplantation substudy (appendix p 13–14). We investigated whether chemotherapy-only, reduced-intensity conditioned allograft using FMA was safe and could improve survival of patients with *BCR-ABL1*-negative or *BCR-ABL1*-positive acute lymphoblastic leukaemia in first complete remission by reducing relapse and TRM. Eligibility for UKALL14 was broad (see protocol in appendix p 40), accepting patients with B-cell or T-cell acute lymphoblastic leukaemia, aged 25–65 years (*BCR-ABL1*-negative) and 18–65 years (*BCR-ABL1*-positive). Patients aged 41 years and older who were in remission after induction were directed to reduced-intensity conditioned allogeneic HSCT and patients younger than 41 years who were considered unfit for myeloablative conditioning were also eligible. The trial comprised two phases of induction, followed by high-dose methotrexate consolidation. Patients eligible for reduced-intensity conditioned HSCT were then allografted.

Patients were initially eligible if they had a fit, matched sibling donor or an 8 out of 8 allelic matched unrelated donor (HLA-A, HLA-B, HLA-C, and HLA-DR). As the trial proceeded, the protocol was amended on June 20, 2014, to allow 7 out of 8 matched unrelated donors if the patient had high risk cytogenetics or was MRD-positive after the second induction course.

The decision about fitness for transplantation was left to investigators, but was based on cardiac and pulmonary function, performance status, and other comorbidities including hepatic and renal function. Patients with borderline fitness could be discussed with the Substudy chief investigator and HSCT lead (DM). Ethical approval for the trial was obtained from the London–Fulham Research Ethics Committee and written informed consent was obtained in accordance with the Declaration of Helsinki. The trial was managed by the Cancer Research UK and University College London Cancer Trials Centre.

When randomised sample sizes were met, UKALL14 was converted into an observational study, but the patients in the observational study were not eligible for inclusion in the reduced-intensity conditioning substudy.

### Procedures

Pretransplantation chemotherapy is described in the appendix (p 2). In the initial stages of the UKALL14 trial, patients with precursor B-lineage acute lymphoblastic leukaemia were randomly assigned (1:1) to receive chemotherapy alone or chemotherapy plus four doses of intravenous rituximab 375 mg/m^2^ given on days 3, 10, 17, and 24 of induction 1. Patients with B-cell disease were eligible for random assignment irrespective of CD20 status. Patients with T-cell disease were randomly assigned (1:1) to receive intravenous nelarabine 1·5 mg/m^2^ on days 1, 3, and 5 or no additional therapy as consolidation after induction 2. Patients with *BCR-ABL1*-positive disease received continuous daily oral imatinib from day 1, starting at 400 mg and escalating to 600 mg throughout induction. After induction, because of the absence of CNS-directed therapy in the chemotherapy-only conditioning regimen, all patients were scheduled to receive two doses of intravenous methotrexate (3 g/m^2^).

During transplantation conditioning, patients received intravenous fludarabine 30 mg/m^2^ on days –6 to –2, melphalan 140 mg/m^2^ on day –2, and alemtuzumab 30 mg on day –1 (sibling donor) and days –2 and –1 (unrelated donor) along with intravenous ciclosporin-A prophylaxis, switching to oral ciclosporin-A before discharge, with concentrations maintained according to institutional practice. Ciclosporin concentrations were used to guide dosage, with weaning recommended at day 40–60 in the absence of graft-versus-host disease (GVHD). Supportive care was delivered according to institutional guidelines, but antibacterial, antiviral, and antifungal prophylaxis were recommended, as was anti-*Pneumocystis jirovecii* prophylaxis for 12 months. The use of tyrosine-kinase inhibitors after the allogeneic HSCT was left to the discretion of investigators, and data on their use were not collected. Adverse event reporting focused on the first 30 days after allogeneic HSCT. Adverse events meeting the definition of serious adverse events had to be reported within 24 h of learning of the event. Adverse events were graded according to Common Terminology Criteria for Adverse Events (version 4.0).

MRD response was assessed on bone marrow aspirates by clonal immunoglobulin (immunoglobulin and T-cell receptor gene rearrangement quantification or *BCR-ABL1* transcript quantification for patients with Philadelphia-positive acute lymphoblastic leukaemia) after induction two, with the result supplied to centres as part of the risk assessment. MRD was assessed every 3 months for 2 years after transplantation within a EuroMRD-accredited central laboratory and was reported per European Leukaemia Network guidelines as positive (numerical value given), positive outside the quantitative range (which was considered as negative for the purpose of the study), negative, or indeterminate, where the sensitivity of the assay did not reach that which was required. MRD of less than 0·01% was considered negative.

Multilineage lymphohaematopoietic chimerism was assessed in the peripheral blood every 3 months for 2 years (appendix p 2). Chimerism of more than 95% donor was considered full donor, below this proportion was considered as mixed chimerism. Mixed chimerism in T cells at 6 months was managed by escalating donor lymphocyte infusion doses starting at 1 × 10^6^ cells per kg, increasing by half a log every 3 months if there was no GVHD and full donor chimerism was not reached. Donor lymphocyte infusions could be given at any time for molecular or haematological relapse, at any level of disease (ie, any detectable leukaemia by molecular MRD or morphology), and at any dose the investigator chose, although investigators were invited to discuss this with the substudy chief investigator and HSCT lead (DM). The dose recommended varied with the level of relapse, type of donor, and whether there was antecedent GVHD.

For the quality of life assessment, patients surviving at 4 years after allogeneic HSCT were asked to complete the General Health Questionnaire-12 (GHQ-12), which has psychological and social domains. Further details of the patients assessed, the scoring system and comparator group are shown in the appendix (p 2).

### Outcomes

The primary endpoint of UKALL14 and the transplantation substudy was event-free survival, with events defined as relapse or death of any cause. Secondary endpoints were overall survival (with an event defined as death by any cause), relapse incidence (haematological relapse defined as ≥5% blasts), safety, and quality-of-life. There were also prespecified transplantation-specific endpoints of TRM, grade 2–4 and grade 3–4 acute GVHD and chronic GVHD (limited or extensive), rates of mixed chimerism and conversion to full donor chimerism after donor lymphocyte infusion, and MRD after transplantation, including the effect of donor lymphocyte infusion.

### Statistical analysis

This study was a single-arm design embedded in the UKALL14 randomised trial. There was no formal sample size calculation for the reduced-intensity conditioned allogeneic HSCT question, with all patients given an FMA reduced-intensity conditioned HSCT as study treatment to be included and analysed. All time-to-event endpoints are from date of transplantation until first event, with patients not having the event censored at the date last seen. Kaplan-Meier survival analyses, Cox regression, and the log-rank-test were used in analyses of event-free survival and overall survival. The assumption of proportional hazards was tested using the Schoenfeld residuals and linearity of continuous variables was assessed by testing the deviance between the continuous-power polynomial model for the variable and a model which is linear for the variable. For relapse and non-relapse mortality, competing risks analysis by the method of Fine and Gray was used, with death in first complete remission or relapse treated as competing risks. Multivariable analyses were performed for all time-to-event outcomes using complete cases (no imputation for missing data), and included the following prespecified variables: age, sex, cell type, Haematopoietic Cell Transplantation-specific Comorbidity Index score, white blood cell count risk group, cytogenetic group, MRD at the end of phase 2 induction, donor, and body-mass index. The model was reduced using backwards selection with p=0·05 for inclusion. Quality of life and analyses of adverse events were descriptive, and the safety of donor lymphocyte infusion for mixed chimerism was assessed using a time varying covariates Cox model, which included donor lymphocyte infusion given for mixed chimerism as a covariate (time to donor lymphocyte infusion taken as the time from transplantation to first donor lymphocyte dose). The effect of GVHD on time-to-event endpoints was analysed in a similar manner, including acute and chronic GVHD as time varying covariates. Chimerism analyses were mainly descriptive, with time-to-event comparisons (mixed chimerism *vs* full donor chimerism) carried out as for the whole population, but with the origin date taken as that of the 6-month chimerism sample. All patients receiving a study FMA reduced-intensity conditioned allogeneic HSCT were included in the analyses, unless otherwise specified. Acute GVHD was analysed in all engrafting patients; chronic GVHD was analysed in all patients in complete remission at day 100. All analyses were done in STATA (version 16.1). This study is registered with ClinicalTrials.gov, NCT01085617.

### Role of the funding source

The funder of the study had no role in study design, data collection, data analysis, data interpretation, or writing of the report.

## Results

**Figure 1:**
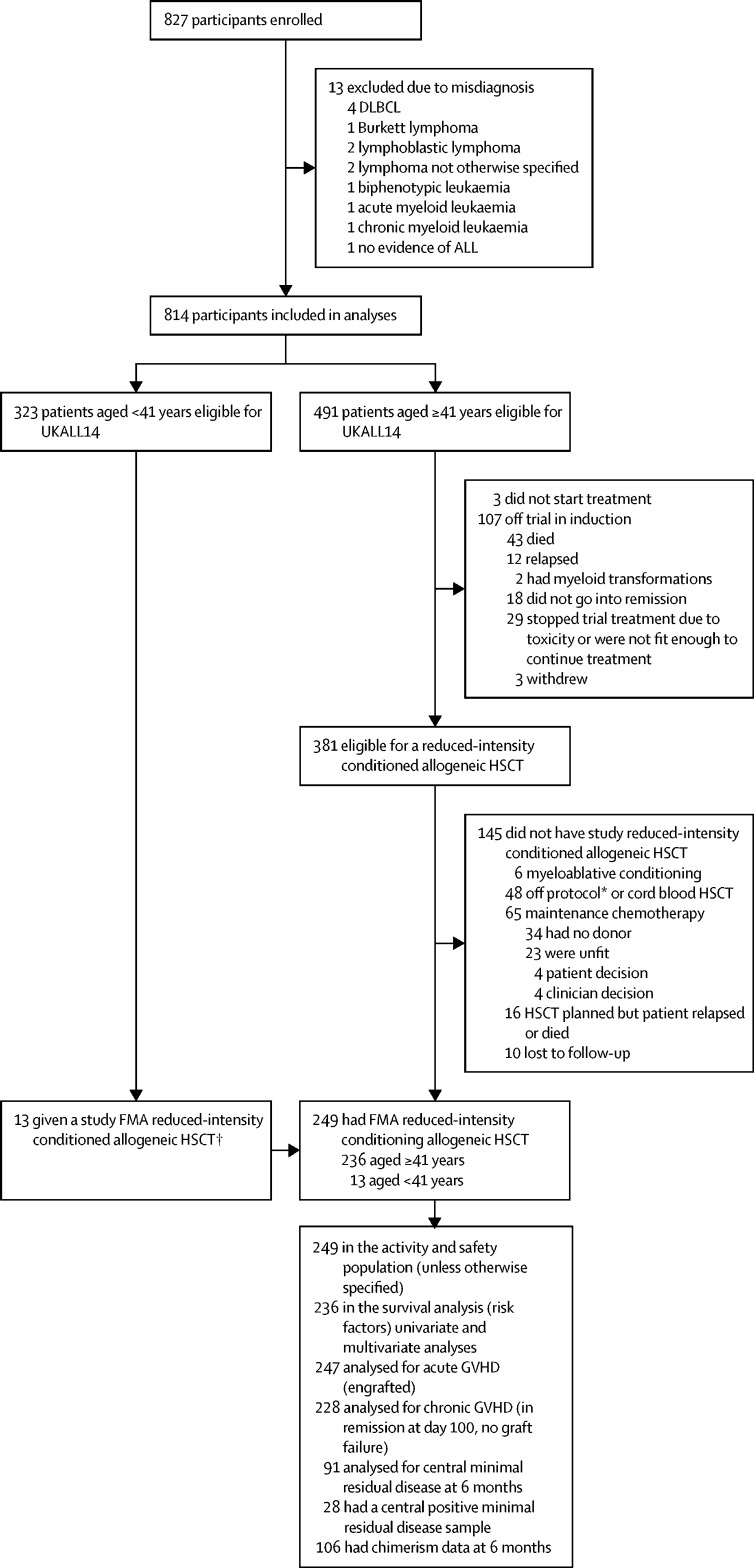
Study profile ALL=acute lymphoblastic leukaemia. DLBCL=diffuse large B-cell lymphoma. FMA=fludarabine, melphalan, and alemtuzumab. GVHD=graft-versus-host disease. HSCT=haematopoietic stem-cell transplantation. *Patient taken off trial for non-protocol conditioning or donor. †Clinician choice based on patient fitness (excluded from univariable and multivariable survival analysis).

Between Feb 22, 2011, and July 26, 2018, 827 participants were enrolled in UKALL14 and 814 were included in analyses (figure 1). 491 patients in the trial were aged 41 years and older, of whom 110 were not eligible for reduced-intensity conditioned allogeneic HSCT and 145 of those who were potentially eligible did not receive reduced-intensity conditioned allogeneic HSCT for the reasons outlined in figure 1. 54 patients were treated by investigators in ways that were considered as deviations from the protocol: eight (15%) received cord blood, six (11%) received myeloablative conditioning, 12 (22%) received transplantations from non-trial donors, six (11%) were in other HSCT trials, 19 (35%) received non-trial conditioning, one (2%) moved site, one (2%) received additional pretransplantation therapy, and the details of one (2%) are unknown. 236 patients aged 41 years and older and a further 13 patients who were younger than 41 years therefore received an FMA reduced-intensity conditioned HSCT, bringing the total cohort to 249 patients from 46 centres (figure 1).

Median age was 50 years (IQR 45–55), 138 (55%) of 249 patients were male, 111 (45%) were female, and ethnicity data were not collected. Baseline demographics and clinical characteristics are shown in table 1. Of the 162 patients who were analysable for molecular MRD after induction chemotherapy, 113 (70%) were considered MRD-negative (72 [44%] negative, 41 [25%] positive outside of quantitative range) and 49 (30%) were positive. Median time from MRD sampling to HSCT was 69 days (IQR 55–90). Time from complete remission to transplantation was similar in patients with sibling and unrelated donors (123 days [IQR 107–151] *vs* 128 days [106–150]; p=0·57).

**Table 1:** Patient demographics and clinical characteristics

	**Enrolled patients (n=249)**
**Treatment group**	
Standard phase 1 alone	110 (44%)
Standard phase 1 plus rituximab	101 (41%)
Standard phase 1 and 2 alone	25 (10%)
Standard phase 1 and 2 plus nelarabine	13 (5%)
**Cell type**	
B-precursor cell disease	211 (85%)
T-cell disease	38 (15%)
**Age group**	
Younger than 41 years at random assignment	13 (5%)
41 years or older at randomisation	236 (95%)
Age, years	50 (45–55)
**Sex**	
Male	138 (55%)
Female	111 (45%)
**Donor**	
Sibling	88/248 (35%)
8 of 8 matched unrelated donor	154/248 (62%)
7 of 8 matched unrelated donor	5/248 (2%)
Mismatched unrelated donor	1/248 (<1%)
**ECOG performance status at end of induction**	
0	107/218 (49%)
1	98/218 (45%)
2	12/218 (6%)
3	1/218 (<1%)
Baseline white blood cell count*	
Standard risk	194 (74%)
High risk	34 (14%)
**BCR-ABL1**	
Negative†	185/248 (75%)
Positive	63/248 (25%)
**Cytogenetic group as used in analyses**	
None	87/193 (45%)
*BCR-ABL1*-positive	63/193 (33%)
Other UKALL14 high-risk cytogenetics‡	43/193 (22%)
**Minimal residual disease after induction chemotherapy**§	
Negative or positive outside quantitative range	113/162 (70%)
Positive	49/162 (30%)
**Time to transplantation, days**	
From end of phase 1 induction	128 (111–153)
From end of phase 2 induction	70 (56–90)
From minimal residual disease sampling to transplantation	69 (55–90)

Data are n (%) or median (IQR). ECOG=Eastern Cooperative Oncology Group.

* More than 30 × 10^9^ cells per L for B cells and more than 100 × 10^9^ cells per L for T cells.

† 55 *BCR-ABL1*-negative patients did not have other cytogenetic data available.

‡ Other high risk: t (4,11), low hypodiploidy or near triploidy and complex karyotype.

§ 87 patients had unknown or indeterminate minimal residual disease in phase 2.

Median time to myeloid engraftment was 12 days (IQR 11–15) and to platelet engraftment was 11 days (10–12). Graft failures were reported in four (2%) patients (two primary and two secondary graft failures). Of the four patients with graft failure, two received cells from unrelated donors and both died; one of thrombosis at 7 months and the second of infection at 13 months. The other two received sibling donor allogeneic HSCT: one was alive 36 months after a second haploidentical donor transplantation and the other was in remission 18 months after the transplantation with no second allogeneic HSCT reported.

**Figure 2:**
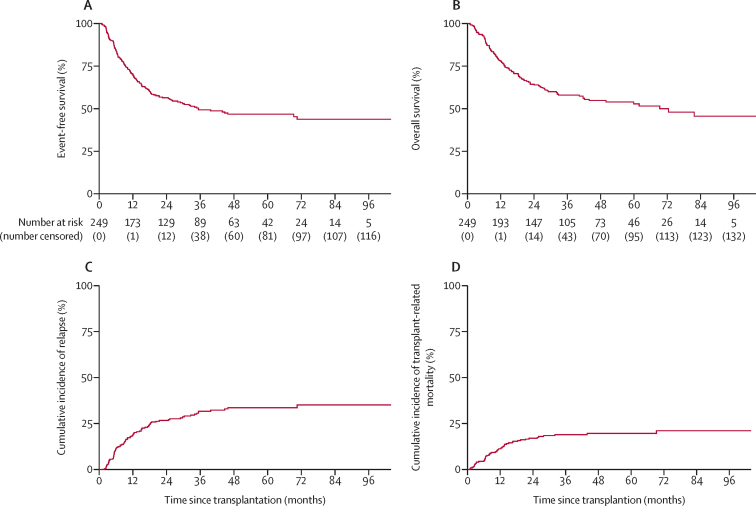
Major outcomes of reduced-intensity conditioned allogeneic haematopoietic stem-cell transplantation (A) Event-free survival. (B) Overall survival. (C) Cumulative incidence of relapse. (D) Cumulative incidence of transplant-related mortality.

After median follow-up of 48·9 months (IQR 35·7–70·3), there were 128 event-free survival events and 112 deaths. 4-year event-free survival was 46·8% (95% CI 40·1–53·2) and 4-year overall survival was 54·8% (48·0–61·2; figure 2A, B). In the *BCR-ABL1*-positive B-cell group, event-free survival was 41·7% (28·6–54·2) and overall survival was 57·6% (43·8–69·2) compared with event-free survival of 45·4% (36·8–53·6) and overall survival of 51·5% (42·6–59·6) in the *BCR-ABL1*-negative B-cell group (appendix p 10).

On univariable analysis, high-risk white-blood-cell count, older age, and MRD-positivity were associated with inferior event-free survival but in multivariable analysis, only age (HR [for an increase of 10 years] 1·93 [95% CI 1·30–2·86]; p=0·0011) and MRD-positivity (2·40 [1·46–3·93]; p=0·0005) were associated with a significant decrease in event-free survival (table 2). 4-year event-free survival for MRD-positive patients was 27·3% (95% CI 15·0–41·2) and for MRD-negative patients was 60·8% (48·0–71·4). Age and MRD-positivity were also the only two factors associated with poorer overall survival in both univariable and multivariable analysis (table 2). Of the six mismatched unrelated donor recipients, one was alive at 24·4 months and one was alive at 36·4 months after HSCT.

**Table 2:** Univariable and multivariable analyses of factors affecting event-free survival and overall survival

		All cases*			**Complete cases**†			**Multivariable analysis**‡	
		Events/N	HR (95% CI)	p value	Events/N	HR (95% CI)	p value	HR (95% CI)	p value
**Event-free survival**									
Age (increase of 10 years)		122/236	1·79 (1·34–2·40)	0·0010	65/120	2·04 (1·38–3·01)	0·0003	1·93 (1·30–2·86)	0·0011
Sex									
	Male	67/134	1 (ref)	0·86	38/70	1 (ref)	0·73	..	..
	Female	55/102	1·03 (0·72–1·47)	..	27/50	0·92 (0·56–1·50)	..	..	..
Cell type									
	B cell	109/202	1 (ref)	0·20	59/105	1 (ref)	0·38	..	..
	T cell	13/34	0·69 (0·39–1·22)	..	6/15	0·69 (0·30–1·59)	..	..	..
HCT-CI score		114/223	1·01 (0·91–1·13)	0·82	65/120	1·00 (0·87–1·15)	0·98	..	..
Baseline white blood cell count									
	Standard risk	93/190	1 (ref)	0·042	47/90	1 (ref)	0·36	..	..
	High risk	29/46	1·54 (1·02–2·34)	..	18/30	1·29 (0·75–2·22)	..	..	..
High-risk cytogenetics									
	None	41/81	1 (ref)	0·67	25/48	1 (ref)	0·73	..	..
	*BCR-ABL1*-positive	23/41	1·13 (0·71–1·77)	..	13/23	1·09 (0·63–1·87)	..	..	..
	Other high risk	34/60	1·26 (0·75–2·09)	..	27/49	1·31 (0·67–2·57)	..	..	..
MRD phase 2									
	Negative (including POQR)	49/106	1 (ref)	0·0010	36/81	1 (ref)	0·0002	1 (ref)	0·0005
	Positive	34/48	2·16 (1·39–3·36)	..	29/39	2·59 (1·58–4·24)	..	2·40 (1·46–3·93)	..
Donor									
	Sibling	44/81	1 (ref)	0·52	26/43	1 (ref)	0·31	..	..
	Unrelated donor	78/155	0·89 (0·61–1·28)	..	39/77	0·77 (0·47–1·27)	..	..	..
Body-mass index									
	Normal or underweight	60/131	1 (ref)	0·44	36/81	1 (ref)	0·53	..	..
	Overweight	44/84	1·31 (0·83–2·09)	..	24/47	1·26 (0·66–2·41)	..	..	..
	Obese	47/85	1·30 (0·82–2·06)	..	26/42	1·44 (0·76–2·71)	..	..	..
**Overall survival**									
Age (increase of 10 years)		106/236	1·98 (1·44–2·71)	<0·0001	53/120	2·40 (1·55–3·73)	0·0001	2·32 (1·49–3·63)	0·0002
**Sex**									
	Male	60/134	1 (ref)	0·96	31/70	1 (ref)	0·90	..	..
	Female	46/102	0·99 (0·67–1·45)	..	22/50	0·96 (0·56–1·67)	..	..	..
Cell type									
	B cell	94/202	1 (ref)	0·41	48/105	1 (ref)	0·53	..	..
	T cell	12/34	0·78 (0·43–1·42)	..	5/15	0·74 (0·30–1·87)	..	..	..
HCT-CI score		98/223	1·06 (0·94–1·18)	0·34	53/120	1·03 (0·89–1·21)	0·68	..	..
Baseline white blood cell count									
	Standard risk	82/190	1 (ref)	0·17	38/90	1 (ref)	0·40	..	..
	High risk	24/46	1·38 (0·87–2·17)	..	15/30	1·30 (0·71–2·36)	..	..	..
High-risk cytogenetics									
	None	36/81	1 (ref)	0·83	21/48	1 (ref)	0·87	..	..
	*BCR-ABL1*-positive	20/41	1·04 (0·63–1·70)	..	11/23	1·01 (0·55–1·85)	..	..	..
	Other high risk	28/60	1·18 (0·69–2·05)	..	21/49	1·20 (0·58–2·50)	..	..	..
MRD phase 2									
	Negative	42/106	1 (ref)	0·0080	30/81	1 (ref)	0·0050	1 (ref)	0·011
	Positive	28/48	1·92 (1·18–3·10)	..	23/39	2·20 (1·27–3·80)	..	2·05 (1·18–3·54)	..
Donor									
	Sibling	35/81	1 (ref)	0·66	18/43	1 (ref)	0·72	..	..
	Unrelated donor	71/155	1·10 (0·73–1·64)	..	35/77	1·11 (0·63–1·96)	..	..	..
Body-mass index									
	Normal or underweight	50/131	1 (ref)	0·28	30/81	1 (ref)	0·46	..	..
	Overweight	39/84	1·44 (0·87–2·40)	..	21/47	1·48 (0·72–3·08)	..	..	..
	Obese	42/85	1·44 (0·87–2·38)	..	21/42	1·55 (0·75–3·21)	..	..	..

HCT-CI=Haematopoietic Cell Transplantation-specific Comorbidity Index. HR=hazard ratio. MRD=minimal residual disease. POQR=positive outside quantitative range.

* All cases includes all patients with data for the specific risk factor.

† Complete cases includes only patients with data for all risk factors (ie, the group used in the multivariable analysis).

‡ All variables included in a backwards selection model with p=0·05 for inclusion (the same model is chosen with forwards selection). No change in conclusions if treatment group is included (and group not selected).

4-year event-free survival in patients aged 41 years and older was similar in those given maintenance (45·6% [95% CI 32·7–57·6]) and those with non-protocol transplantations (55·6% [41·0–68·0]). However, treatment was not randomly allocated and deviations were likely to be related to fitness and risk factors. The proportion of patients with high-risk white blood cell count or cytogenetics at baseline was similar among those who received maintenance therapy and those who received allogeneic HSCT (32 [49%] of 65 *vs* 116 [49%] of 236) but slightly lower than patients receiving non-protocol HSCT (32 [59%] of 54; p=0·18). The maintenance group had non-significantly lower MRD-positivity rates compared with the allogeneic HSCT group (seven [22%] of 33 *vs* 48 [31%] of 154; p=0·26).

80 patients had relapsed at a median of 9·5 months (IQR 5·2–17·8), resulting in a cumulative incidence of 33·6% (95% CI 27·9–40·2) at 4 years (figure 2C). Cell lineage did not affect relapse incidence (appendix p 4). 62 (78%) 80 relapses were in the bone marrow only, ten (13%) were isolated to the CNS, and eight (10%) were extramedullary, half of which also had bone marrow relapse and one also included the CNS. Cumulative incidence of any CNS relapse at 4 years was 4·2% (95% CI 2·3–7·6) with a median time of those who relapsed of 5·3 months (IQR 2·3–17·9) from transplantation until relapse. Cumulative incidence of bone marrow relapse at 4 years was 28·0% (95% CI 22·6–34·3) with a median time of those who relapsed of 9·3 months (IQR 5·4–16·3) from transplantation until relapse. None of the ten patients with CNS relapse had CNS involvement at baseline. Nine (90%) of the ten patients with CNS relapse died at a median of 2·6 months (IQR 1·5–17·6) after relapse. Management of relapse is described in the appendix (p 3).

Only MRD-positivity after induction and donor type were significantly associated with relapse in univariable or multivariable analysis (appendix p 5). Patients who were MRD-positive at the end of induction had more than double the risk of relapse (hazard ratio [HR] 2·41 [95% CI 1·29–4·48]; p=0·0054) and patients with unrelated donors had half the risk of relapse compared with patients with sibling donors (0·47 [0·25–0·87]; p=0·016). High-risk cytogenetics and baseline white blood cell count were not significantly associated with relapse (appendix p 5), although both groups had higher rates of MRD-positivity than those with standard-risk cytogenetics and white blood cell counts (33 [27%] of 121 with standard-risk white blood cell counts *vs* 15 [46%] of 33 with high-risk white blood cell count; p=0·046 and 12 [24%] of 50 with standard-risk cytogenetics *vs* 18 [34%] of 53 *BCR-ABL1*-positive *vs* 11 [44%] of 25 other high risk; p=0·20).

64 (80%) of 80 patients died after relapse. Median overall survival after relapse was 6·7 months (95% CI 4·7–11·7), with 18·3% (95% CI 10·2–28·2) surviving 2 years. Median overall survival in patients with *BCR-ABL1*-positive disease was 14·1 months (95% CI 6·6–47·8), with *BCR-ABL1*-negative disease was 6·7 months (3·2–11·4), and with T-cell disease was 2·7 months (0·7–5·3; appendix p 10). Patients who relapsed after a longer period from HSCT survived longer (HR per month after HSCT 0·97 [95% CI 0·94–1·00]; p=0·026). Year of relapse did not significantly affect survival (data not shown).

More than half of the patients who died, died after relapse (63 [57%] of 112). TRM occurred in 48 (19%) of 249 patients, with infection being the most common cause of death (27 [11%] of 249) and GVHD the cause of death in seven (3%) patients.

TRM had a cumulative incidence of 3·6% (95% CI 1·9–6·8) at 100 days and 19·6% (15·1–25·3) at 4 years (figure 2D). Median time to TRM was 10·5 months (IQR 6·2–15·8). Older age was the only factor that was significantly associated with TRM in univariable or multivariable analysis and was associated with a more than doubling of the risk of a TRM event for each 10-year increase in age (HR 2·39 [95% CI 1·24–4·62]; p=0·010; appendix p 6).

Excluding patients with non-melanomatous skin cancers and one patient with non-Hodgkin lymphoma after relapse, there were 12 patients with second cancer events (four [2%] with melanomas, two [1%] with post-transplantation lymphoproliferative disorders, one [<1%] with lung cancer, one [<1%] with prostate cancer, one [<1%] with a neuroendocrine tumour, one [<1%] with myelodysplastic syndrome, one [<1%] with myeloproliferative disorder, and one [<1%] with oesophageal cancer). Three patients with second cancer events died: two (1%) with post-transplantation lymphoproliferative disorders and one (<1%) patient with non-small-cell lung cancer. The 4-year cumulative incidence of second cancer was 4·0% (95% CI 2·1–7·7). Details of acute adverse events are in table 3 and the appendix (pp 7–9).

**Table 3:** Adverse events reported during conditioning and up to 30 days after transplantation in 232 patients who had reduced-intensity conditioned HSCT

		**Grade 1–2**	**Grade 3**	**Grade 4**	**Grade 5**
Blood and lymphatic system disorders		66 (28%)	135 (58%)	..	0
	Anaemia	76 (33%)	119 (51%)	..	0
	Febrile neutropenia	..	63 (27%)	..	0
Gastrointestinal disorders		147 (63%)	59 (25%)	..	0
	Diarrhoea	144 (62%)	..	..	0
	Oral mucositis	69 (30%)	29 (13%)	..	0
	Nausea	135 (58%)	25 (11%)	..	0
	Vomiting	69 (30%)	..	..	0
General disorders and administration site conditions		153 (66%)	..	..	0
	Fatigue	48 (21%)	..	..	0
	Fever	99 (43%)	..	..	0
Immune system disorders		42 (18%)	..	..	0
	Allergic reaction	37 (16%)	..	..	0
Infections and infestations		43 (19%)	80 (34%)	..	3 (1%)
	Lung infection	..	..	..	1 (<1%)
	Sepsis	..	..	..	2 (<1%)
Investigations		..	..	192 (83%)	0
	Neutrophil count decreased	..	..	181 (78%)	0
	Pancytopenia	..	..	24 (10%)	0
	Platelet count decreased	..	..	61 (26%)	0
	White blood cell decreased	..	..	168 (72%)	0
Metabolism and nutrition disorders		70 (30%)	37 (16%)	..	0
	Anorexia	54 (23%)	..	..	0
Musculoskeletal and connective tissue disorders		42 (18%)	..	..	0
Nervous system disorders		79 (34%)	..	..	1 (<1%)
	Headache	63 (27%)	..	..	0
	Leukoencephalopathy	..	..	..	1 (<1%)
Respiratory, thoracic, and mediastinal disorders		56 (24%)	..	..	0
Skin and subcutaneous tissue disorders		120 (52%)	..	..	0
	Alopecia	44 (19%)	..	..	0
Vascular disorders		..	..	..	1 (<1%)
	Thromboembolic event	..	..	..	1 (<1%)
Any adverse event		..	..	193 (83%)	5 (2%)

Events presented: all grade 5 events, grade 3 and 4 events occurring in 10% or more patients and grade 1–2 events occurring in 15% or more patients. Full details of all grade 3–4 events and grade 1–2 events occurring in 10% or more patients are shown in the appendix (pp 7–9). Patients with more than one event are counted once in the system organ class row but are shown in multiple individual adverse event type rows; if a grade 3 or 4 event occurred in less 10% of patients or a grade 1–2 event occurred in less than 15% of patients that event type is not listed but is included in the total events for that system organ class. 17 patients did not return adverse events forms or have serious adverse events reported and are not included in the denominator. HSCT=haematopoietic stem-cell transplantation.

247 patients engrafted and were evaluable for acute GVHD. 29 (12%) patients had grade 2–4 GVHD and 12 (5%) patients had grade 3–4 GVHD. 61 (25%) patients had grade 1 GVHD, giving a total incidence of any grade of GVHD of 91 (37%). One (<1%) patient died of acute GVHD. 20 (20%) of 99 patients had additional GVHD after donor lymphocyte infusion (eight [8%] with grade 1 GVHD, six [6%] with grade 2, and six [6%] with grade 3). 13 (65%) of the 20 patients who had additional GVHD after donor lymphocyte infusion had one donor lymphocyte dose, five (25%) had two doses, one (5%) had three doses, and one (5%) had an unknown number of doses. The median maximum donor lymphocyte dose was 1 × 10^6^ cells per kg (IQR 1 × 10^6^ – 5 × 10^6^). Five (83%) of six patients who received donor lymphocyte infusion and had grade 3 GVHD had known donor lymphocyte doses; the median maximum dose for these patients was 1 × 10^6^ cells per kg (range 3 × 10^5^–5 × 10^6^).

228 patients were alive and in remission at day 100 and were analysable for chronic GVHD. 84 (37%) of these patients had chronic GVHD: 34 (15%) had limited chronic GVHD and 50 (22%) had extensive chronic GVHD. 22 (10%) of these patients had antecedent acute GVHD. Median onset of chronic GVHD was at 5·9 months (IQR 4·5–9·3) after HSCT. The cumulative incidence of any GVHD at 1 year was 53·0% (95% CI 47·0–59·3) and at 2 years was 55·9% (49·9–62·2). The cumulative incidence of GVHD was higher in unrelated donor recipients than in sibling donor recipients at 2 years (62% [54·5–69·5] *vs* 45·6% [35·9–56·6]; log-rank p=0·0020).

Multivariable models including GVHD as time-varying covariates (acute GVHD as grade 1–2 or 3–4 and chronic GVHD as limited or extensive) and baseline risk factors showed that chronic GVHD conferred better event-free survival and overall survival, with improvements in relapse risk (data not shown). However, extensive chronic GVHD was associated with TRM (HR 3·32 [95% CI 1·49–7·39]; p=0·0033). There were no significant differences in event-free survival or overall survival associated with acute GVHD (data not shown).

33 patients received donor lymphocyte infusions for positive MRD at a median of 10 months (IQR 7–14) after HSCT. Median number of doses was 1 (IQR 1–2) with a median dose of 1·2 × 10^6^ cells per kg (IQR 1 × 10^6^–1 × 10^7^) and a maximum dose of 8 × 10^7^ cells per kg. 16 (44%) of 33 patients relapsed (median 39·0 months [IQR 13·1–not reached]), four (12%) died in remission (two [6%] from infection, two [6%] from GVHD). 13 (39%) patients were in continuous remission, with a median follow-up after donor lymphocyte infusion dose one of 35·9 months (IQR 27·6–53·4). Ten (77%) of these 13 patients had at least one MRD-negative sample reported.

Of 206 patients alive and in remission without graft failure at 6 months after transplantation (ie, assessable for multilineage chimerism), 106 (51%) had chimerism samples analysed. 44 (42%) of 106 had full donor chimerism and 62 (48%) had mixed chimerism. Of the 62 patients with mixed chimerism, 46 (74%) were given donor lymphocyte infusions, and 38 (90%) of 42 who were subsequently assessed had full donor chimerism at a median of 5·5 months (IQR 3·3–9·3) after the first donor lymphocyte infusion. There was no difference in event-free survival, overall survival, or relapse rate between patients with and without mixed chimerism at 6 months (data not shown). However, patients with full donor had higher TRM than those with mixed chimerism (4 year rates: 29·5% [95% CI 17·7–46·7] *vs* 9·9% [4·6–20·8]; p=0·032) but there was no difference between these groups in lethal GVHD.

To assess donor lymphocyte infusion safety, patients reported to have been given donor lymphocyte infusion for mixed chimerism at any point (including those without multilineage chimerism data available) were assessed. At a median of 6·3 months (IQR 4·7–8·3) 70 patients were treated with a median of 1 dose (IQR 1–2) and median dose 1 × 10^6^ cells per kg (IQR 5 × 10^5^–3 × 10^6^). Multivariable models for event-free survival, overall survival, relapse rate, and TRM, including donor lymphocyte infusion as a time-varying covariate, showed no increased risk of any endpoint associated with donor lymphocyte infusion given for mixed chimerism (data not shown).

27 (56%) of 48 patients with TRM had one or more infections as the named cause of death. Seven (15%) patients had fungal infections, 13 (27%) patients had bacterial infections (six gram-negative), and 11 (23%) had viral infections (three cytomegalovirus and two Epstein-Barr virus). Median time-to-death from infection was 12·3 months (IQR 4·1–18·4). Of 27 patients with infection-related TRM, 17 (63%) had GVHD, but GVHD was not considered the cause of death.

For 28 analysable patients, the median GHQ-12 score was 1·0 (IQR 0·0–7·5). 11 (39%) patients had a GHQ-12 score of 0, five (18%) had a score of 1–3, and 12 (43%) had a score of 4 or more. Two (17%) of 12 patients with a GHQ-12 score of 4 or more had extensive chronic GVHD.

## Discussion

To our knowledge, this was the largest prospective trial of reduced-intensity conditioned allogeneic HSCT in adult acute lymphoblastic leukaemia using a uniform conditioning regimen in consecutively accrued patients within a national trial. UKALL14 opened in 77 centres recruiting more than 65% of the incident population (recruiting a mean of 138 patients per year of 205 registered diagnoses in the cohort of patients aged 25–65 years). UKALL14 elected to investigate reduced-intensity conditioned allogeneic HSCT because of the poor results of chemotherapy in this age group.^2^ Reduced-intensity conditioned allogeneic HSCT, which uses lower doses of chemotherapy or radiotherapy, is used to permit older, less fit patients to receive allogeneic HSCT with lower TRM. This therapy confers a lower direct cytotoxic antileukaemic effect and a greater reliance on the graft-versus-leukaemia effect than HSCT with myeloablative conditioning, which is crucial in the treatment of acute lymphoblastic leukaemia.^8^ The alternative of T-replete reduced-intensity conditioned allogeneic HSCT,^5^ especially with an unrelated donor, would expose older patients to a risk of severe acute and extensive chronic GVHD, which are poorly tolerated with high mortality.

In this study, in-vivo T-cell depletion with alemtuzumab was added to the well established fludarabine and melphalan regimen^5^ in an attempt to reduce severe GVHD, knowing that there would be a balance of risks with a possible increase in relapse. However, in patients with acute lymphoblastic leukaemia, this risk might be partly offset by the direct antileukaemic effect of alemtuzumab on CD52-positive B cells and T cells.^9^

In our study, at 4 years, event-free survival was 46·8% (95% CI 40·1–53·2) and overall survival was 54·8% (48·0–61·2) in a high-risk, older population, which is very encouraging and—with the caveat that these patients were selected as being fit for HSCT—better than previously reported outcomes.^1^, ^2^, ^3^ Event-free survival from UKALL12 was not reported, but 5-year overall survival in (younger) patients aged 35–55 years was 41%, compared with 52·8% (45·7–59·4) at 5 years in our current study (appendix p 3), in which 95% of the population were aged 41–65 years. A limitation of our study was that we did not do a randomised comparison of reduced-intensity conditioned allogeneic HSCT with non-transplantation therapy;^10^, ^11^ however, a pretrial feasibility survey suggested that such a study would fail to recruit. Similar event-free survival rates were reported by Peric and colleagues,^12^ who compared three reduced-intensity conditioning regimens in 417 patients older than 45 years with acute lymphoblastic leukaemia in first complete remission. Event-free survival was 42–45%, with slightly higher relapse rates (40%).^12^ However, Peric and colleagues’ study did not clearly define genetic risk factors.

Reduced-intensity conditioned allogeneic HSCT was less effective in patients who were MRD-positive before transplantation (38·4% [95% CI 23·8–52·8] 4-year overall survival). Although this might be better than with chemotherapy, which results in 12% 3-year overall survival,^13^ additional strategies are clearly needed. Blinatumomab before transplantation (which leads to molecular complete remission in 78% of patients with persistent MRD) could be beneficial,^14^ but it remains to be seen whether the chemoresistance conferred by high-risk genetics in patients with acute lymphoblastic leukaemia can be overcome by this drug. Post-allograft strategies, such as pre-emptive donor lymphocyte infusions or blinatumomab,^15^ might also be worthwhile.

Ten (4·2% [95% CI 2·3–7·6]) of 249 patients experienced CNS relapse at 4 years. This rate was not unexpected, although the literature on reduced-intensity conditioned allogeneic HSCT does not separately report CNS relapse incidence, so we have no direct comparator. The absence of total body irradiation and specific CNS-directed therapy in our protocol was a concern, so eight doses of post-allogenic-HSCT intrathecal therapy were included; however, we did not randomise this intervention and are unable to assess its efficacy.

The main cause of treatment failure was relapse. Notably, relapse was less frequent in recipients of transplantations from unrelated donors, and in patients who had chronic GVHD, confirming the importance of the graft-versus-leukaemia effect. The low incidence of extensive chronic GVHD is very important because chronic GVHD affects TRM and quality of life.^16^ GHQ-12 scores at 4 years after transplantation suggested preserved quality of life in most survivors, although 12 (43%) of 28 patients had scores of 4 or more. However, only 28 (47%) of 59 4-year survivors returned quality-of-life forms, limiting conclusions from these data.

This trial also investigated if pre-emptive donor lymphocyte infusion for mixed chimerism at 6 months after allogeneic HSCT could be safely given and prevent relapse. We showed that full donor chimerism can be reached after donor lymphocyte infusion. Donor lymphocyte infusion was largely safe, with grade 2–4 GVHD seen in 6% of patients after infusion. However, insufficient patients received door lymphocyte infusion for mixed chimerism to evaluate if relapse was reduced by achieving full donor chimerism. Therefore, whether correcting mixed chimerism with donor lymphocyte infusion reduces relapse remains an open question. Van Besien and colleagues^17^ reported that mixed chimerism in 120 alemtuzumab-conditioned allografts did not portend relapse, but this study did not specifically focus on patients with acute lymphoblastic leukaemia. With the median time-to-relapse in this study being 9·5 months (IQR 5·2–17·8), planning to give the first pre-emptive donor lymphocyte infusion at 6 months might be too late, and earlier intervention should be tested.

18·3% (95% CI 10·2–28·2) of patients survived 2 years after relapsing after HSCT, suggesting a possible effect of novel targeted therapies after allograft;^10^, ^11^, ^18^ an outcome that is likely to improve. For B-cell disease, blinatumomab and inotuzumab lead to complete remission in 40–80% of patients and might prolong survival, but there is little published evidence for second transplantation in adults with acute lymphoblastic leukaemia, leaving chimeric antigen receptor (CAR) T cells as the only proven curative therapy. Recently published data in older age groups suggest efficacy but possibly more toxicity compared with children receiving CAR T cells.^19^, ^20^

In our study, overall survival was similar and TRM was slightly lower than the 22% seen with fludarabine plus melphalan without T-cell depletion in an older population (median age 58 years).^5^ Furthermore, our study achieved its goal of providing effective anti-acute lymphoblastic leukaemia therapy with much lower toxicity compared with that in UKALL12 (36% 2-year TRM in the 35–55 year age group).^1^ As expected, the most frequent cause of TRM was infection after the allogeneic HSCT (27 [56%] of 48 cases). Alemtuzumab results in prolonged lymphopenia, predisposing patients to cytomegalovirus and Epstein-Barr virus infection. Viral PCR surveillance and early treatment is crucial. A counterbalance to high rates of infection was the very low incidence of fatal GVHD (seven [3%] of 249 patients) in our study, compared with that seen with the T-cell replete fludarabine plus melphalan conditioning regimen, which has been associated with mortality in seven (9·7%) of 72 participants from extensive chronic GVHD.^5^ The 4% incidence of second cancers in this study at 4 years suggests that continued cancer surveillance after allogeneic HSCT is warranted,^21^ especially in this older population.

This study had two major limitations. First, we are unable to reliably compare outcomes from the transplanted group with the 65 patients who investigators elected to treat with maintenance chemotherapy. This group was non-randomised and likely to differ from the cohort who received reduced-intensity conditioned allogeneic HSCT in both fitness and relapse risk, so we had to rely on historical comparators. Randomised leukaemia studies comparing transplantation and non-transplantation therapy are rarely undertaken, so other ways of comparing outcomes should be used. Second, our protocolised administration of donor lymphocyte infusion for patients with mixed chimerism was successful in leading to full donor chimerism, but patient numbers were insufficient for us to assess the effect on relapse. Large studies with greater patient numbers will be required to examine this issue.

For older patients with B-cell disease, an alternative strategy would be intravenous cyclophosphamide, vincristine, and dexamethasone (without anthracycline) plus inotuzumab,^22^ reserving reduced-intensity conditioned allogeneic HSCT for patients who don’t reach MRD-negativity or who subsequently relapse. Alternative strategies in *BCR-ABL1*-positive patients include regimens that contain blinatumomab and dasatinib (or ponatinib), although 38% of *BCR-ABL1*-positive patients had allogeneic HSCT.^23^ Reduced-intensity conditioned allogeneic HSCT might be used after CAR T-cell therapy where there is failed persistence (ie, no evidence of CAR T cells beyond 3 months).^20^

In summary, this large, UK-wide study provides practice-informing evidence for the role of reduced-intensity conditioned allogeneic HSCT in older adults with acute lymphoblastic leukaemia in first complete remission. We are currently investigating in ALL-RIC (ISRCTN 99927695) whether improved outcomes can be obtained by enhanced conditioning,^24^ and by giving post-transplantation donor lymphocyte infusion for mixed chimerism at earlier timepoints. Furthermore, following an exploration of the activity and safety of different alemtuzumab doses and schedules,^25^ in an attempt to reduce infection and relapse, we elected to use a single 30 mg dose in sibling and unrelated donor recipients.

## Data sharing

This study had no data-sharing plan, requests should be made to DIM and will be considered by the chief investigator (AKF).

## Declaration of interests

DIM reports educational events, consulting, and advisory boards for Pfizer, Amgen, Kite, and Novartis. MC declares grants or contracts with Incyte and Cyclacel; speakers bureau or honoraria for Incyte, Novartis, Pfizer, Astellas, Jazz, and Gilead; and advisory boards for Novartis, Pfizer, Jazz, and Daiichi-Sankyo. AM received educational honoraria and payments for advisory boards and travel sponsorship from Roche; advisory board honoraria from Amgen; educational and travel payments from BMS and Celgene; and advisory board honoraria from AbbVie. TFM declares travel grants from Amgen, Jazz, Pfizer, Bayer, Kyowa Kirin, Celgene, Kite/Gilead, Janssen, and Takeda; advisory board honoraria from Kite/Gilead, Amgen, Novartis, Pfizer, Celgene, Daiichi Sankyo, Atara, and Roche; lecture honoraria from Kite/Gilead, Takeda, Janssen, Roche, Servier, Novartis, and Celgene; and research funding from Janssen, AstraZeneca, and Novartis. AVM received honoraria for an educational event for Amgen. MNP received honoraria and meeting support from Kite. NM received speaker fees from Amgen, Janssen, and AbbVie; attended advisory boards of AbbVie and Kite; and had conference support from AbbVie and Takeda. CJR reports paid educational events for Kite and Incyte; and advisory boards for Kite, Novartis, Amgen, and Pfizer. AKF reports consulting for Amgen. BP received honoraria from Pfizer and Amgen. All other authors declare no competing interests.
