# Supplementary material for: In-vivo T-cell depleted reduced-intensity conditioned allogeneic haematopoietic stem-cell transplantation for patients with acute lymphoblastic leukaemia in first remission: results from the prospective, single-arm evaluation of the UKALL14 trial
Source: Lancet Haematol. 2022 Mar 28;9(4):e276–88. doi: 10.1016/S2352-3026(22)00036-9 (PMC8969058; doi:10.1016/S2352-3026(22)00036-9)
Supplement: Supplementary appendix [file mmc1.pdf]

THE LANCET
Haematology

Supplementary appendix

This appendix formed part of the original submission and has been peer reviewed. We post it as supplied by the authors.

Supplement to: Marks DI, Clifton-Hadley L, Copland M, et al. In-vivo T-cell depleted reduced-intensity conditioned allogeneic haematopoietic stem-cell transplantation for patients with acute lymphoblastic leukaemia in first remission: results from the prospective, single-arm evaluation of the UKALL14 trial. *Lancet Haematol* 2022; **9:** e276–88.

Prospective single-arm evaluation of *in vivo* T-cell depleted reduced intensity allogeneic hematopoietic stem cell transplantation (alloHSCT) for acute lymphoblastic leukemia in first remission: results from the UK ALL14 trial

Supplementary material

Methods

Pretransplant chemotherapy

The original phase 1 and phase 2 induction chemotherapy has been described in detail[1] . It was then modified because of excessive hepatotoxicity and mortality. Eighteen patients in this study's cohort were recruited pre-amendment. BCR-ABL1 negative patients eligible for a RIC transplant had only one dose of PEG asparaginase at day 18 and BCR-ABL1 positive patients did not receive asparaginase. All patients had lower doses of daunorubicin (30mg/m^2).

Multilineage chimerism and minimum residual disease

In the central laboratory StraightFrom™ MACS Microbeads were used on post-transplant peripheral blood samples, to positively select the desired lymphocyte and myeloid cell lineages. From extracted genomic DNA of the donor and recipient, polymorphic short tandem repeat sequences (STR) of the stem cell recipient and donor were PCR amplified and sequence-based typing using the Promega powerplex 16® system. Optimal STR loci that demonstrate suitable size differences between the transplant pair can then be selected for monitoring of donor/recipient chimerism. Quantification can then be measured by the percentage area of each peak.

Patients with indeterminate results (the assay did not reach the required level of accuracy) or non-assessable MRD were treated as standard risk within the trial protocol, but have been excluded from all analyses of MRD as a risk factor. Some sites also chose to analyse molecular MRD during follow-up in six accredited regional laboratories that support paediatric ALL trials in the UK.

Quality of life assessment

Quality-of-life forms were completed by 34 patients but patients who had relapsed or had second cancers were excluded. This left 28 patients analysable, out of the 59 survivors who have reached the 4 year time point (47.5%). A total score of 0 is no evidence of probable mental ill heath, 1-3 indicates less than optimal mental health and 4 or more is probable psychological disturbance or mental ill health and has been validated in a 3-year follow up study of alloHSCT patients[2,3]. We found no relevant patient group that had been subjected to the GHQ-12 for comparison and elected to compare RIC alloHSCT patients with the general population of England, thought to have a score ≥4 in between 17 and 20%, although this was likely to be slightly higher within the UKALL14 RIC age group (41-65) [4].

Definitions

Engraftment was defined as the first of 3 consecutive days the neutrophil count was >0.5 x 10^9/l. High-risk ALL consisted of one or more of: presenting white cell count >30x 10^9/l (B-cell), >100x10^9/l (T-cell), t(9;22), KMT2A-AFF1, low hypodiploidy (<40 chromosomes)/near triploidy, complex karyotype (≥5 abnormalities), any positive MRD at the end of induction phase 2 and age ≥41 years.

Results

Overall survival

For the purposes of comparison to UKALL XII we also estimated the 5-year survival in a group aged 35-55 years, this was 52.8% (95% CI: 45.7 – 59.4).

Relapse

Therapeutic options for treatment of relapse evolved over the course of the study with greater access to targeted therapies. Of the 65 B-cell patients who relapsed, 16 had blinatumomab, 7 inotuzumab, and 10 had CAR-T cells. Patients receiving the newer targeted therapies had a median survival of 12.5 months compared to 0.8 months for those receiving best supportive care. Six of the 10 CAR-T cell patients currently survive.

Second transplant post-relapse was infrequently performed (n=4) and was not routinely funded in the UK during the course of the study.

Infectious complications

CMV reactivation is a common complication of alemtuzumab-containing transplants but viral reactivation data were not collected and it was not routinely reported as an SAE as most patients were managed with valganciclovir or IV ganciclovir as outpatients.

The detection of EBV reactivation and the trigger for using rituximab will have varied in the transplant centres and is likely to have changed over the 7 years of study accrual. We did not collect these data. EBV-associated PTLD was reported in 2 patients (0.8%), both of whom died.

Other fatal viral infections included herpes zoster, human metapneumovirus, polyomavirus (all n=1) and norovirus (n=2).

References

1. Patel B, Kirkwood AA, Dey A, et al . Pegylated-asparaginase during induction therapy for adult acute lymphoblastic leukaemia: toxicity data from the UKALL14 trial. Leukemia. 2017 Jan;31(1):58-64.

2. Goldberg D and Williams PA. *User Guide to the General Health Questionnaire.* NFER-Nelson, Windsor, UK, 1988.

3. Broers S Kaptein AA, Le Cessie S, Fibbe W and Hengeveld MW. Psychological functioning and quality of life following bone marrow transplantation: A 3-year follow-up study. J Psychosomatic Res 48:11-21, 2000.

4. Health Survey for England 2016: Well-being and mental health.

http://healthsurvey.hscic.gov.uk/media/63763/HSE2016-Adult-wel-bei.pdf. Published December 13, 2017.

Supplementary Table 1: Univariable and multivariable analyses of factors affecting Time to relapse

	All cases			Complete cases			Multivariable	
	Events/N	HR (95% CI)	p-value	Events/N	HR (95% CI)	p-value	HR (95% CI)	p-value
**Age (increase of 10 years)**	76/236	1.17(0.81 - 1.68)	0.41	42/120	1.40(0.87 - 2.24)	0.16		
**Sex**								
Male	44/134	1.00	0.67	25/70	1.00	0.72		
Female	32/102	0.91(0.57 - 1.43)		17/50	0.89(0.48 - 1.65)			
**Cell type**								
B-cell	65/202	1.00	0.78	37/105	1.00	0.94		
T-cell	11/34	1.09(0.58 - 2.07)		5/15	1.04(0.41 - 2.64)			
**HCTCI score**	71/223	0.94(0.82 - 1.09)	0.43	42/120	1.01(0.85 - 1.22)	0.87		
**Baseline WCC**								
Standard risk	60/190	1.00	0.61	32/90	1.00	0.92		
High risk	16/46	1.15(0.66 - 2.00)		10/30	0.96(0.47 - 1.96)			
**High risk Cytogenetics**								
None	27/81	1.00	0.70	16/48	1.00	0.37		
BCR-ABL1 +	16/41	1.01(0.57 - 1.79)		10/23	0.97(0.48 - 1.94)			
Other high risk	21/60	1.28(0.69 - 2.38)		16/49	1.63(0.74 - 3.60)			
**MRD Phase 2**								
Negative (inc POQR)	32/106	1.00	0.040	23/81	1.00	0.016	**1.00**	**0.0054**
Positive	22/48	1.77(1.03 - 3.05)		19/39	2.12(1.15 - 3.89)		**2.41 (1.29 – 4.48)**	
**Donor**								
Sibling	33/81	1.00	0.031	20/43	1.00	0.045	**1.00**	**0.016**
MUD	43/155	0.61(0.39 - 0.96)		22/77	0.54(0.29 - 0.99)		**0.47 (0.25 – 0.87)**	
**BMI**								
Normal/Underweight	36/131	1.00	0.94	23/81	1.00	0.82		
Overweight	25/84	0.97(0.54 - 1.73)		14/47	0.81(0.37 - 1.75)			
Obese	29/85	1.07(0.61 - 1.87)		16/42	0.99(0.47 - 2.09)			

*The assumption of proportional hazards was tested using the Schoenfeld residuals and linearity of continuous variables was assessed by testing the deviance between the continuous-power polynomial model for the variable and a model which is linear for the variable.*All variables included in a backwards selection model with p=0.05 for inclusion. (Same model is chosen with forwards selection). No change in conclusions if treatment arm is included (and arm not significant)*

Supplementary Table 2: Univariable and multivariable analyses of factors affecting treatment related mortality

	All cases			Complete cases			Multivariable	
	Events/N	HR (95% CI)	p-value	Events/N	HR (95% CI)	p-value	HR (95% CI)	p-value
**Age (increase of 10 years)**	46/236	2.57(1.58 - 4.17)	0.0001	23/120	2.39(1.24 - 4.62)	0.0096	**2.39 (1.24 – 4.62)**	**0.010**
**Sex**								
Male	23/134	1.00	0.37	13/70	1.00	0.92		
Female	23/102	1.30(0.73 - 2.32)		10/50	1.04(0.46 - 2.38)			
**Cell type**								
B-cell	44/202	1.00	0.057	22/105	1.00	0.23		
T-cell	2/34	0.25(0.06 - 1.04)		1/15	0.30(0.04 -2.20)			
**HCTCI score**	43/223	1.10(0.94 - 1.29)	0.23	23/120	0.99(0.77 - 1.27)	0.92		
**Baseline WCC**								
Standard risk	33/190	1.00	0.081	15/90	1.00	0.24		
High risk	13/46	1.77(0.93 - 3.37)		8/30	1.67(0.71 - 3.93)			
**High risk Cytogenetics**								
None	14/81	1.00	0.75	9/48	1.00	0.62		
BCR-ABL1 +	7/41	1.31(0.62 - 2.79)		3/23	1.25(0.52 - 3.02)			
Other high risk	13/60	1.01(0.41 - 2.51)		11/49	0.68(0.18 - 2.51)			
**MRD Phase 2**								
Negative (inc POQR)	17/106	1.00	0.15	13/81	1.00	0.16		
Positive	12/48	1.73(0.83 - 3.62)		10/39	1.80(0.79 - 4.10)			
**Donor**								
Sibling	11/81	1.00	0.097	6/43	1.00	0.28		
MUD	35/155	1.78(0.90 - 3.50)		17/77	1.67(0.66 - 4.23)			
**BMI**								
Normal/Underweight	11/81	1.00	0.33	13/81	1.00	0.29		
Overweight	35/155	1.78(0.90 -3.50)		10/47	2.41(0.66 - 8.78)			
Obese	18/85	1.64(0.74 - 3.66)		10/42	2.68(0.74 - 9.75)			

*The assumption of proportional hazards was tested using the Schoenfeld residuals and linearity of continuous variables was assessed by testing the deviance between the continuous-power polynomial model for the variable and a model which is linear for the variable.*All variables included in a backwards selection model with p=0.05 for inclusion. (Same model is chosen with forwards selection). No change in conclusions if treatment arm is included (and arm not significant)*

Supplementary table 3: Adverse events reported during conditioning and up to 30 days post-transplant (full list)

System Organ Class\adverse events	RIC patients N=232			
	Grade 1-2 N(%)	Grade 3 N(%)	Grade 4 N(%)	Grade 5 N(%)
**Blood and Lymphatic System Disorders**	**66 (28.4)**	**135 (58.2)**	**7 (3.0)**	**0**
Anaemia	76 (32.8)	119 (51.3)	0	0
Febrile Neutropenia	-	63 (27.2)	7 (3.0)	0
Blood and Lymph Other: NOS	-	1 (0.4)	0	0
**Cardiac Disorders**	**27 (11.6)**	**5 (2.2)**	**0**	**0**
Pericardial Effusion	-	4 (1.7)	0	0
Sinus Tachycardia	-	1 (0.4)	0	0
**Eye Disorders**	**-**	**0**	**1 (0.4)**	**0**
Vitreous Haemorrhage	-	0	1 (0.4)	0
**Gastrointestinal Disorders**	**147 (63.4)**	**59 (25.4)**	**5 (2.2)**	**0**
Abdominal Pain	-	2 (0.9)	0	0
Colitis	-	1 (0.4)	0	0
Constipation	24 (10.3)	0	0	0
Diarrhoea	144 (62.1)	20 (8.6)	1 (0.4)	0
Dysphagia	-	1 (0.4)	0	0
Esophagitis	-	3 (1.3)	0	0
Mucositis Oral	69 (29.7)	29 (12.5)	4 (1.7)	0
Nausea	135 (58.2)	25 (10.8)	0	0
Vomiting	69 (29.7)	3 (1.3)	0	0
Other: Focal Active Colitis	-	1 (0.4)	0	0
**General Disorders and Administration Site Conditions**	**153 (65.9)**	**13 (5.6)**	**0**	**0**
Other: Peripheral Oedema - No Site Specified	24 (10.3)	0	0	0
Back Pain	-	0	1 (0.4)	0
Oedema Limbs	28 (12.1)	0	0	0
Fatigue	48 (20.7)	1 (0.4)	0	0
Fever	99 (42.7)	5 (2.2)	0	0
Pain	32 (13.8)	7 (3.0)	0	0
Pain in Extremity	-	1 (0.4)	0	0
**Immune System Disorders**	**42 (18.1)**	**0**	**1 (0.4)**	**0**
Allergic Reaction	37 (15.9)	0	0	0
Anaphylaxis	-	0	1 (0.4)	0
**Infections and Infestations**	**43 (18.5)**	**80 (34.5)**	**13 (5.6)**	**3 (1.3)**
Abdominal Infection	-	1 (0.4)	0	0
Catheter Related Infection	-	5 (2.2)	0	0
Device Related Infection	-	22 (9.5)	0	0
Enterocolitis Infectious	-	1 (0.4)	0	0
Gum Infection	-	1 (0.4)	0	0
Lung Infection	-	3 (1.3)	2 (0.9)	1 (0.4)
Mucosal Infection	-	11 (4.7)	0	0
Otitis Media	-	1 (0.4)	0	0
Sepsis	-	6 (2.6)	10 (4.3)	2 (0.9)
Skin Infection	-	4 (1.7)	0	0
Small Intestine Infection	-	1 (0.4)	0	0
Upper Respiratory Infection	-	6 (2.6)	0	0
Urinary Tract Infection	-	5 (2.2)	0	0
Other: Adenovirus	-	1 (0.4)	0	0
Other: Bacterial Infection NOS	-	16 (6.9)	2 (0.9)	0
Other: Blood Infection	-	1 (0.4)	0	0

System Organ Class\adverse events	RIC patients N=232			
	Grade 1-2 N(%)	Grade 3 N(%)	Grade 4 N(%)	Grade 5 N(%)
Other: Bone Marrow Infection	-	0	1 (0.4)	0
Other: C Diff	-	1 (0.4)	0	0
Other: CMV	-	3 (1.3)	0	0
Other: Disseminated Varicella Zoster	-	1 (0.4)	0	0
Other: Fungal Infection NOS	-	2 (0.9)	0	0
Other: Neutropenic Sepsis	-	0	1 (0.4)	0
Other: Unknown	-	8 (3.4)	0	0
Other: Viral Infection NOS	-	5 (2.2)	0	0
Other: VRE	-	1 (0.4)	0	0
**Investigations**	**-**	**15 (6.5)**	**192 (82.8)**	**0**
Alanine Aminotransferase Increased	27 (11.6)	4 (1.7)	0	0
Blood Bilirubin Increased	24 (10.3)	2 (0.9)	2 (0.9)	0
GGT Increased	-	1 (0.4)	1 (0.4)	0
Granulocytopenia	-	0	20 (8.6)	0
Lymphocyte Count Decreased	-	0	23 (9.9)	0
Myelosuppression	-	3 (1.3)	13 (5.6)	0
Neutrophil Count Decreased	-	19 (8.2)	181 (78.0)	0
Pancytopenia	-	6 (2.6)	24 (10.3)	0
Platelet Count Decreased	-	2 (0.9)	61 (26.3)	0
Urine Output Decreased	-	1 (0.4)	0	0
Weight Loss	29 (12.5)	2 (0.9)	0	0
White Blood Cell Decreased	-	7 (3.0)	168 (72.4)	0
**Metabolism and Nutrition Disorders**	**70 (30.2)**	**37 (15.9)**	**2 (0.9)**	**0**
Anorexia	54 (23.3)	16 (6.9)	0	0
Hyperglycaemia	-	2 (0.9)	1 (0.4)	0
Hyperkalaemia	-	1 (0.4)	0	0
Hypertriglyceridemia	-	1 (0.4)	0	0
Hypoalbuminemia	24 (10.3)	5 (2.2)	0	0
Hypocalcaemia	-	2 (0.9)	0	0
Hypoglycaemia	-	1 (0.4)	0	0
Hypokalaemia	-	16 (6.9)	1 (0.4)	0
Hypomagnesaemia	28 (12.1)	2 (0.9)	0	0
Hyponatraemia	-	1 (0.4)	0	0
Hypophosphatemia	-	3 (1.3)	0	0
**Musculoskeletal and Connective Tissue Disorders**	**42 (18.1)**	**2 (0.9)**	**1 (0.4)**	**0**
Back Pain	-	0	1 (0.4)	0
Bone Pain	-	1 (0.4)	0	0
Pain	32 (13.8)	7 (3.0)	0	0
Pain in Extremity	-	1 (0.4)	0	0
**Nervous System Disorders**	**79 (34.1)**	**9 (3.9)**	**1 (0.4)**	**1 (0.4)**
Dizziness	-	1 (0.4)	0	0
Headache	63 (27.2)	3 (1.3)	0	0
Leukoencephalopathy	-	0	0	1 (0.4)
Syncope	-	2 (0.9)	1 (0.4)	0
Tremor	-	1 (0.4)	0	0
Vasovagal Reaction	-	1 (0.4)	0	0
Other: Encephalitis	-	1 (0.4)	0	0
**Psychiatric Disorders**	**28 (12.1)**	**4 (1.7)**	**0**	**0**
Anxiety	-	1 (0.4)	0	0
Confusion	-	1 (0.4)	0	0
Depression	-	3 (1.3)	0	0
**Renal and Urinary Disorders**	**29 (12.5)**	**2 (0.9)**	**1 (0.4)**	**0**
Acute Kidney Injury	-	1 (0.4)	1 (0.4)	0

System Organ Class\adverse events	RIC patients N=232			
	**Grade 1-2 N(%)**	**Grade 3 N(%)**	**Grade 4 N(%)**	**Grade 5 N(%)**
Urine Output Decreased	-	1 (0.4)	0	0
**Respiratory, Thoracic and Mediastinal Disorders**	**56 (24.1)**	**6 (2.6)**	**3 (1.3)**	**0**
Dyspnoea	-	1 (0.4)	0	0
Hypoxia	-	1 (0.4)	1 (0.4)	0
Pleural Effusion	-	1 (0.4)	0	0
Pulmonary Oedema	-	1 (0.4)	0	0
Respiratory Failure	-	0	2 (0.9)	0
Sore Throat	-	3 (1.3)	0	0
**Skin and Subcutaneous Tissue Disorders**	**120 (51.7)**	**7 (3.0)**	**0**	**0**
Alopecia	44 (19.0)	0	0	0
Erythema Multiforme	-	1 (0.4)	0	0
Rash Maculo-Papular	-	1 (0.4)	0	0
Urticaria	-	1 (0.4)	0	0
Other: Rash (NOS)	30 (12.9)	3 (1.3)	0	0
Skin Other: NOS	-	1 (0.4)	0	0
**Vascular Disorders**	**-**	**0**	**0**	**1 (0.4)**
Hypertension	-	8 (3.4)	0	0
Hypotension	-	1 (0.4)	0	0
Thromboembolic Event	-	1 (0.4)	0	1 (0.4)
**Any AE**	**-**	**21 (9.1)**	**193 (83.2)**	**5 (2.2)**

Events presented: all grade 3,4 and 5 events and grade 1-2 events occurring in 10% or more patients. N=17 patients did not return AE forms or have SAEs reported and are not included in the denominator.

Supplementary figure 1 A-B

A. Event Free survival by BCR-ABL 1 positivity

B. Overall survival by BCR-ABL 1 positivity

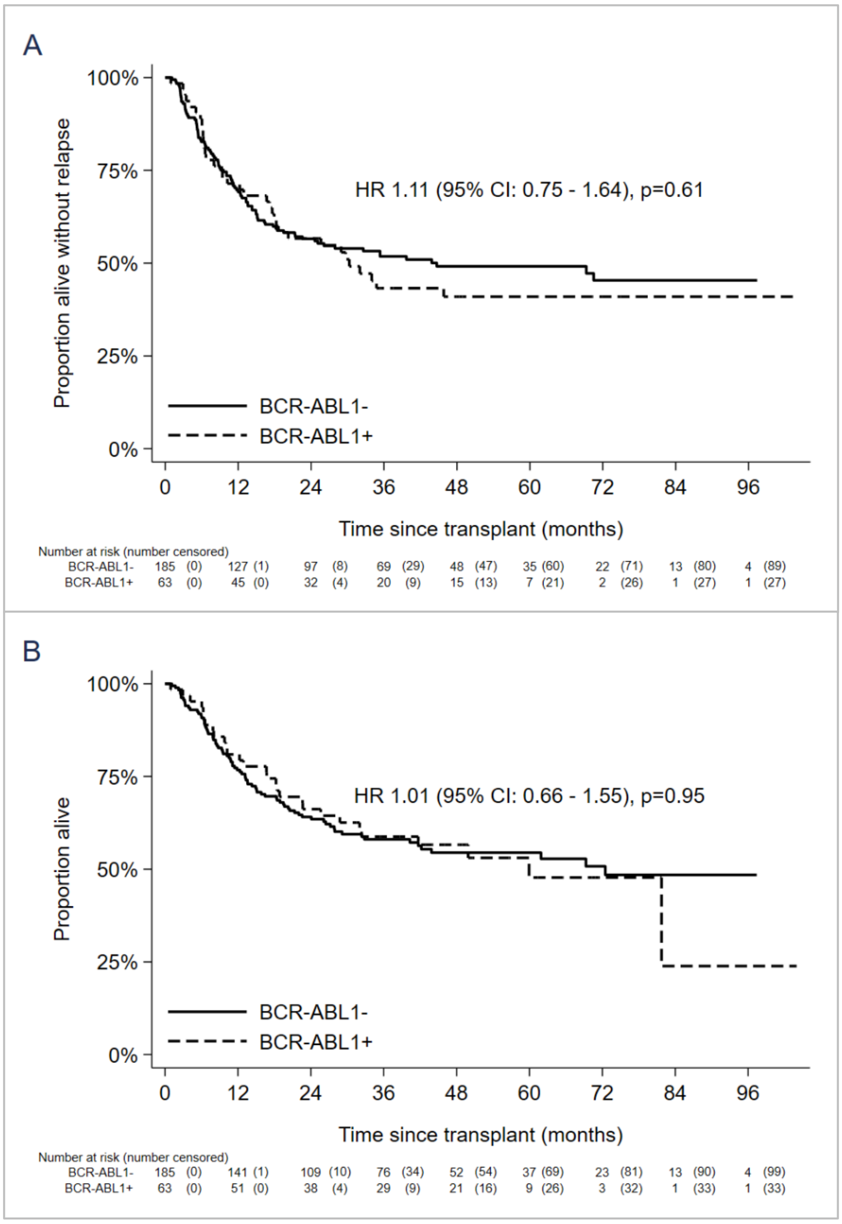

Supplementary figure 2 A-B

A. Event Free survival by pre-transplant minimum residual disease status

B. Overall survival by pre-transplant minimum residual disease status

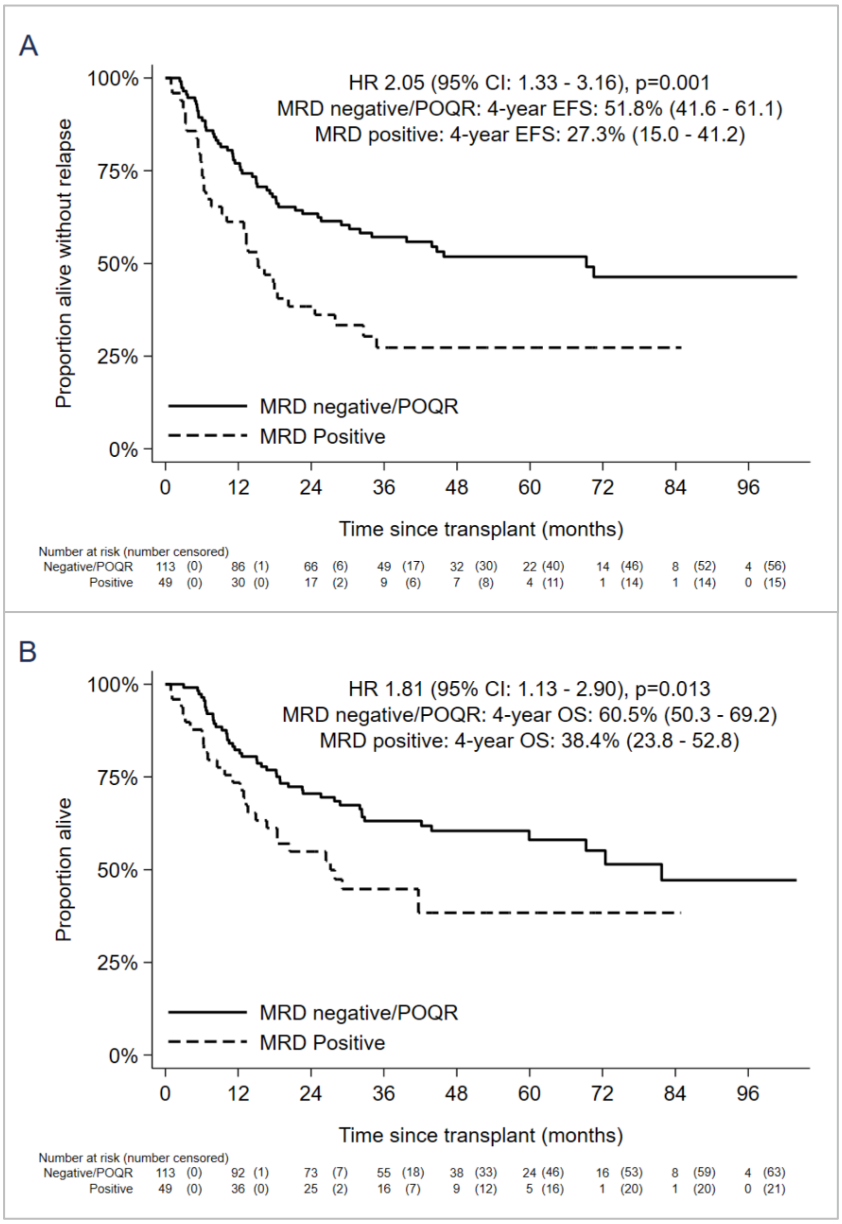

Supplementary figure 3: Overall survival post relapse by BCR-ABL1 and cell type

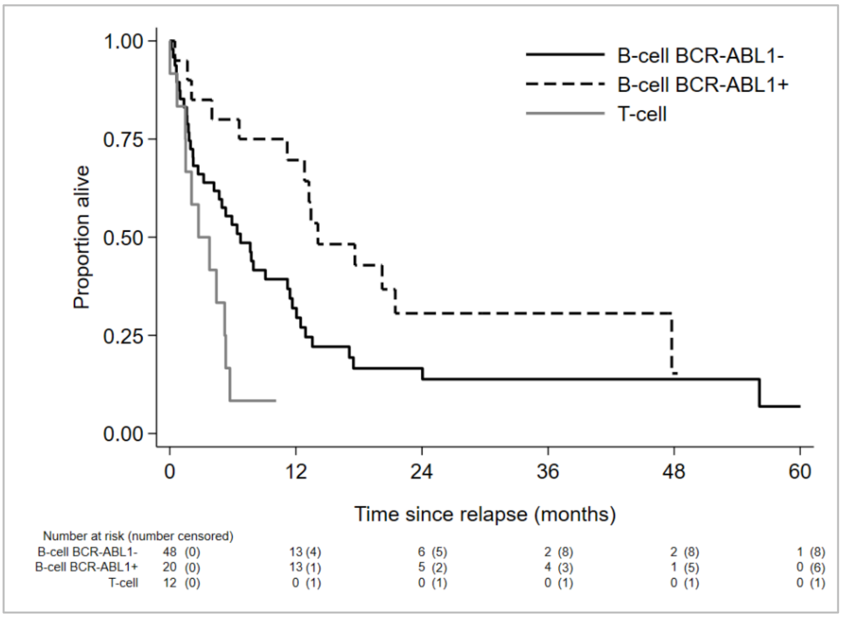

Acknowledgements: the authors wish to thank the following investigators who recruited RIC alloHSCT patients to this study

Centre	Principle investigator(s)	Number of patients
**Beatson West of Scotland Cancer Centre**	Mhairi Copland	20
**Royal Marsden Hospital London**	David Taussig and Michael Potter	17
**Bristol Haematology Oncology Centre**	David Marks	15
**Royal Free Hospital/UCLH**	Adele Fielding and Asim Khwaja	15
**Royal Hallamshire Hospital**	Nicholas Morley	15
**Southampton General Hospital**	Deborah Richardson and Kim Orchard	15
**St James University Hospital**	Richard Kelly and David Bowen	12
**Nottingham City Hospital**	Andrew McMillan	11
**Queen Elizabeth Hospital Birmingham**	Sridhar Chaganti and David Bowen	11
**Blackpool Victoria Hospital**	Paul Cahalin	8
**Freeman Hospital Newcastle**	Tobias Menne and Matthew Collin	8
**Royal Liverpool University Hospital**	Arpad Toth, Richard Clark, Usira Vithanarachchi,R ahuman Salim and Amit Patel	7
**St Bartholomew's Hospital**	Matthew Smith and Heather Oakervee	7
**Christie Hospital**	Anna Castleton and Samar Kulkarni	6
**Derriford Hospital**	Hannah Hunter	6
**Kings College Hospital**	Deborah Yallop and Robert Marcus	6
**University Hospital Wales**	Clare Rowntree	6
**Leicester Royal Infirmary**	Murray Martin and Ann Hunter	5
**Addenbrookes Hospital**	Ram Malladi, Kiran Tawana and Jenny Craig	4
**Basingstoke and North Hampshire Hospital**	Isabella Lo and Sylwia Simpson	4
**St George's Hospital London, London**	Matthisa Klammer	4
**Belfast City Hospital**	Damian Finnegan and Mary-Frances McMullin	3
**John Radcliffe Hospital**	Andy Peniket	3
**Musgrove Park Hospital**	Simon Bolam	3
**New Cross Hospital**	Supratik Basu and Sunil Handa	3
**University Hospital Coventry**	Beth Harrison	3
**Aberdeen Royal Infirmary**	Dominic Culligan	2
**Arrowe Park Hospital**	Ranjit Dasgupta	2
**Hammersmith Hospital**	Jiri Pavlu	2
**Northwick Park Hospital**	Vaitsa Katsomitrou and Nicki Panoskaltsis	2
**Poole General Hospital**	Darshayani Furby and Fergus Jack	2
**Queen Alexandra Hospital**	Mary Ganczakowski	2
**Royal Devon and Exeter Hospital**	David Veale and Jackie Ruell	2
**Russell's Hall Hospital**	Craig Taylor	2
**Singleton Hospital, Swansea**	Unmesh Mohite	2
**Victoria Infirmary Glasgow**	Ian MacDonald	2
**Western General Hospital Edinburgh**	Huw Roddie	2
**Whiston Hospital**	Toby Nicholson	2
**Birmingham Heartlands Hospital**	Joanne Ewing and Don Milligan	1
**Bradford Royal Infirmary**	Anshu Garg, Lisa Newton, Samuel Ackroyd and Nandini Sadasivam	1
**Cheltenham General Hospital**	Adam Rye	1
**Glan Clwyd Hospital**	Earnest Heartin and Christine Hoyle	1
**Manchester Royal Infirmary**	Eleni Tholouli, John Ahman Liu Yin	1
**Royal United Hospital Bath**	Christopher Knecthli	1
**Torbay District General Hospital**	Deborah Turner	1
**University Hospital Aintree**	Jeffery Smith and Walid Sadik	1

UKALL14

A randomized trial for adults with newly diagnosed acute lymphoblastic leukaemia

Trial Sponsor:	**University College London**
Trial Sponsor no:	**UCL/08/0167**
Trial funder:	**Cancer Research UK**
Funder reference:	**C27995/A9609**
Clinicaltrials.gov no:	**NCT01085617**
EUDRACT no:	**2009-012717-22**
CTA ref:	**20363/0273/001**
REC ref:	**09/H0711/90**

Protocol version & date: Version 14.0, 13/10/2021

Protocol v14.0 13/10/2021 authorisation signatures

Name & Role:	Signature:	Date authorised:
Chief Investigator:<br>Professor Adele Fielding<br>Professor of Haematology	------------------------------	------------------------
For the Sponsor:<br><br>Laura Clifton-Hadley<br>Trials Group Lead	------------------------------	------------------------

Please note: This trial protocol must not be applied to patients treated outside the UKALL14 trial. UCL CTC can only ensure that approved trial investigators are provided with amendments to the protocol.

Coordinating Centre:

For general queries, supply of trial documentation and collection of data:

UKALL14 Trial Coordinator
Cancer Research UK & UCL Cancer Trials Centre
90 Tottenham Court Road, London, W1T 4TJ
Tel: +44 (0) 20 7679 9860
Fax: +44 (0) 20 7679 9861
Email: ctc.ukall14@ucl.ac.uk

CTC website: http://www.ctc.ucl.ac.uk/

09:00 to 17:00 Monday to Friday, excluding Bank Holidays (UK time)

Other Trial contacts:

Chief Investigator:	Professor Adele Fielding<br>UCL Cancer Institute<br>Room 418
Address:	Paul O'Gorman Building<br>University College London<br>72 Huntley Street<br>London UK<br>WC1E 6DD
Phone	020 3447 7179
Fax	n/a
Email	a.fielding@ucl.ac.uk

Trial Management Group (TMG):

Member	Role	Institution
Adele Fielding	Professor of Haematology; Scientific Correlative studies and Minimal residual disease	UCLH/ UCL Cancer Institute
Amy Kirkwood	Statistician	UCL CTC
David Marks	Professor of Haematology (UKALL14 Transplant Coordinator)	Bristol Haematology & Oncology Centre
Andrew McMillan	Consultant Haematologist	Nottingham Univeristy Hospital
Anthony Moorman	Scientific Correlative studies and Cytogenetics	Newcastle University
Clare Rowntree	Consultant Haematologist	University Hospital, Wales
Tobias Menne	Consultant Haematologist	The Freeman Hospital
Laura Clifton-Hadley	Trials Group Lead	UCL CTC
Hayley Cartwright	Senior Trial Coordinator	UCL CTC
Kalam Hussain	Trial Coordinator	UCL CTC
Bela Wrench	Scientific Correlative studies and Minimal residual disease	St Bartholomew's Hospital
Krisztina Zuborne Alapi	Scientific Correlative studies and Minimal residual disease	UCL Cancer Institute

Additional Clinical Coordinators:

In addition to the Chief Investigator, clinical advice may be obtained from:

Prof David Marks	Bristol (UKALL14 Transplant Co-ordinator; main contact for transplant-related queries) Email: david.marks@UHBristol.nhs.uk Tel: 0117 342 1117
Dr Andrew McMillan	Nottingham Email: andrew.mcmillan@nhs.net or andrew.mcmillan@nuh.nhs.uk Tel: 0797 373 3729
Dr Clare Rowntree	Cardiff Email: clare.rowntree@wales.nhs.uk Tel: 0779 661 4977

Table of Contents

Coordinating Centre:....3
Other Trial contacts:....3
Trial Management Group (TMG):....3
Table of Contents....5
1.0 Protocol Summary....10
1.1 Summary of Trial Design....10
1.2 Trial schema:....13
2.0 Background....14
2.1 Introduction....14
2.2 Induction therapy for ALL....14
2.3 Steroid for induction of ALL....14
2.4 Monoclonal antibodies in induction of precursor B-lineage ALL....15
2.4.1 Anti CD20 antibody: Rituximab....15
2.5 Nelarabine for induction of T-cell ALL....15
2.6 Role of L-asparaginase....16
2.7 Prophylaxis against Central Nervous System Disease in ALL....17
2.8 Role of Haematopoietic Stem Cell Transplantation (HSCT) in adult ALL....18
2.8.1 Myeloablative conditioning regimen....18
2.8.2 Prevention of oral mucositis – the role of Palifermin....18
2.8.3 Non-myeloablative conditioning regimens in ALL....19
2.8.4 The use of unrelated donors....20
2.8.5 The source of stem cells....20
2.9 Measurement of Minimal Residual Disease (MRD)....20
2.10 'Registration only' sub-study....21
2.11 Summary of study aims....21
3.0 Selection of Trial Sites/Site Investigators....23
3.1 Trial Site selection....23
3.2 Selection of Principal Investigator and other investigators at sites....23
3.2.1 Training requirements for site staff....23
3.3 Site set up....23
3.3.1 Site initiation....24
3.3.2 Site activation (UCL CTC responsibility)....24
4.0 Informed consent....25
5.0 Selection of Patients....26
5.1 Pre-registration & pre-randomisation evaluation (study entry)....26
5.2 Patient Eligibility for Study Entry....27
5.2.1 Patient Inclusion Criteria (study entry)....27
5.2.2 Patient Exclusion Criteria (study entry)....27
5.2.3 Management of patients with poor organ function at study entry....28

5.2.4 Pregnancy, Lactation & Birth Control....28
5.3 Registration & Randomisation Procedures....30
5.3.1 Registration & Randomisations (B & T) at study entry....30
5.3.2 Pre-transplant evaluation....31
5.3.3 Patient eligibility for transplant....32
5.3.3.1 Inclusion criteria (transplant)....32
5.3.4 Procedure for Registration to either consolidation/maintenance or transplant....33
5.3.5 Transfer to a new site for Transplant....33
6.0 Trial drug supply....34
7.0 Trial Treatments....34
7.1 Study drugs....34
7.1.1 Summary of IMPs to be evaluated....34
7.1.2 Summary of non-IMPs....34
7.2 Treatment Schedule....34
7.2.1 Recommended supportive care:....35
7.2.2 Steroid pre-phase....38
7.2.3 Phase 1 induction....38
7.2.4 End of phase 1 induction....41
7.2.5 Phase 2 induction....42
7.2.6 End of phase 2 induction....44
7.2.7 Intensification/Central nervous system prophylaxis....45
7.2.8 Consolidation therapy....47
7.2.9 Maintenance therapy (non-transplant patients only)....53
7.2.10 Transplant conditioning regimens....54
7.2.11 Post-transplant intrathecal therapy....61
7.3 Management after treatment withdrawal....61
7.4 Out-of-hours medical care....61
8.0 Assessments....62
8.1 Assessment time points....62
8.1.1 Routine Clinical and Laboratory Assessments during treatment....62
8.1.2 Schedule of testing for MRD and correlative science testing....63
8.1.3 Assessments during follow-up....64
9.0 Correlative Science....65
9.1 Aim 1. To determine the relationship between CD20 on ALL blasts and response to monoclonal antibody therapy....65
9.1.1 Background....65
9.1.2 Plan of investigation....65
9.2 Aim 2. To determine whether the administration of an anti-B-cell monoclonal antibody as part of induction therapy for ALL limits the extent of anti-asparaginase antibody formation and promotes asparagine depletion....66

9.2.1 Background 66
9.2.2 Plan of Investigation 66
9.3 Aim 3. To perform genomic profiling in order to discover and characterise novel prognostic markers and to identify known copy number alterations (CNA). 66
9.3.1 Background 66
9.3.2 Plan of Investigation 67
9.4 Aim 4. To determine whether the speed at which full donor chimerism is achieved in the T –cell compartment correlates with the level of molecularly determined minimal residual disease. 68
9.4.1 Background 68
9.4.2 Plan of investigation 68
9.5 Aim 5. Assessment of late effects 69
9.5.1 Aims 69
9.5.2 Background & Planned Investigations 69
9.6 Schedule of testing for correlative science studies 70
9.7 Constitutional DNA Samples 70
10.0 Data Collection & Management 72
10.1 Completing Forms 72
10.2 Corrections 72
10.3 Missing Data 72
10.4 Queries 72
10.5 Submission Timelines 72
10.6 Archiving of Trial Documentation 73
11.0 Pharmacy 74
11.1 Pharmacy responsibilities 74
11.2 Drug accountability 74
11.3 Temperature Excursions 74
12.0 Pharmacovigilance 76
12.1 Definitions of Adverse Events 76
12.2 Reporting Procedures 76
12.2.1 All Adverse Events (AEs) 76
12.2.2 Serious Adverse Events (SAEs) 78
12.3 Adverse event of special interest 83
12.4 SUSARs 83
12.5 Safety Monitoring 83
12.6 Pregnancy 84
12.6.1 Pregnancy Follow-Up Reports 84
12.6.2 SAEs During Pregnancy 84
12.6.3 Pregnancy Report Processing at UCL CTC 84
12.7 Development Safety Update Reports (DSURs) 84
13.0 Incident Reporting and Serious Breaches 86

13.1 Incident Reporting 86
13.2 Serious Breaches 86
14.0 Withdrawal of patients 87
15.0 Discontinuation/End of the Trial 89
15.1 Trial closure 89
15.2 Archiving of Trial Documentation 89
15.3 Early discontinuation of trial 89
15.4 Withdrawal from trial participation by sites 89
16.0 Trial Monitoring and oversight 90
16.1 Monitoring 90
16.1.1 Central monitoring 90
16.1.2 'For Cause' On-Site Monitoring 90
16.1.3 Monitoring follow up 91
16.2 Oversight Committees 91
16.2.1 Trial Management Group (TMG) 91
16.2.2 Trial Steering Committee (TSC) 91
16.2.3 Independent Data Monitoring Committee (IDMC) 91
16.2.4 Role of UCL CTC 92
17.0 Statistics 93
17.1 Population for analysis 93
17.2 Analysis of the primary objective(s) 93
17.3 Analysis of secondary objectives 94
17.3.1 Efficacy (secondary) 95
17.3.2 Safety 95
17.4 Sample size calculations 95
17.5 Power for analysis of critical secondary variables 96
17.6 Interim analysis 96
18.0 Ethical and Regulatory Approvals 97
18.1 Ethical Approval 97
18.2 Regulatory Approval 97
18.3 Site Approvals 98
18.4 Protocol Amendments 98
18.5 Patient Confidentiality & DPA 98
19.1 Sponsor Details: 99
19.2 Indemnity: 99
20.0 Publication Policy 100
21.0 References 101
Appendix 1: 'Registration only' sub-study 105
1. Background 105
2. Aims of the 'registration only' sub-study 105

3. Consent of 'registration only' patients 105
4. Pre-registration investigations for 'registration only' patients 106
5. Eligibility for the sub-study 107
5.1 Inclusion criteria 107
5.2 Exclusion criteria 107
6. Registration Procedure for 'registration only' sub-study 107
7. Treatment for 'registration only' patients 108
8. Assessments and data collection 108
8.1 Assessment time points 108
8.2 Routine Clinical and Laboratory Assessments during treatment 109
8.3 Schedule of testing for MRD and correlative science 109
8.4 Assessments during follow up 110
8.5 Transfer of 'registration only' patients to another hospital 110
9. Pharmacovigilance 110
10. Sample size 110
Appendix 2: Abbreviations 112
Appendix 3: IMP Information 115
Appendix 4: Non-IMP General Drug information 119
Appendix 5: Dose modifications for toxicity 125
Appendix 6: Assessment of GVHD 128
Appendix 7: Cytogenetic Definitions and Detection Strategy 130
Appendix 8: Central laboratories and trial schedule 132
Appendix 9: Donor Peripheral Blood Stem Cell Collection & Return 135
Appendix 10: Haematopoietic Cell Transplantation-Specific Comorbidity Index 136
Appendix 11: ECOG performance status 137
Appendix 12: Schedule of Assessments (including testing for MRD & Correlative Science) 138
Appendix 13: General Health Questionnaire (GHQ-12) 140
Appendix 14: Guidance on Use of Erwinia Asparaginase (Erwinase®) in patients with systemic reactions to Pegylated-Asparaginase. 142
Appendix 15: Guideline for the administration of IV High-Dose Methotrexate 144
Appendix 16: Karnofsky Performance Status 149
Appendix 17: Protocol Version History 150

1.0 Protocol Summary

1.1 Summary of Trial Design

**Title:**	A randomized trial for adults with newly diagnosed acute lymphoblastic leukaemia
**Short Title:**	UKALL14
**EuDRACT No.:**	2009-012717-22
**MREC No:**	09/H0711/90
**Clinicaltrials.gov No.:**	NCT01085617
**UK Sponsor & No:**	University College London<br>UCL/08/0167
**UK Funder & No:**	Cancer Research UK<br>C27995/A9609
**Design:**	Multisite, randomised controlled trial
**Aims:**	Aim 1B (precursor-B-lineage) To determine if the addition of Rituximab to standard induction chemotherapy results in improved EFS in patients with precursor B-cell lineage ALL.<br>Aim 1T (T-lineage) To determine if the addition of nelarabine following standard induction therapy (arms T1 and T2) improves outcome for patients with T-cell ALL.<br>Aim 2 To determine the tolerability of pegylated asparaginase in induction (for Philadelphia negative patients) and to compare anti-asparaginase antibody levels between patients in the 2 randomisation groups from aim 1B.<br>Aim 3 To determine whether risk-adapted introduction of unrelated donor HSCT (myeloablative conditioning in patients aged ≤40 years at time of study entry and non-myeloablative conditioning in patients aged ≥41 years at study entry) result in greater EFS for patients at highest risk of relapse.<br>Aim 4 To compare 2 schedules of administration (standard P1 vs. 'collapsed' P2) of keratinocyte growth factor (palifermin) for efficacy in preventing the severe mucosal toxicity of etoposide/TBI HSCT conditioning regimen. (Randomisation closed in April 2016 as no longer clinically relevant)
**Primary endpoint:**	Event free survival (applies to all interventions)<br>Toxicity related to pegylated asparginase (applies to asparaginase evaluation only)
**Secondary endpoints:**	Anti-asparaginase antibodies (induction randomisation only)<br>Overall Survival<br>Complete remission rate<br>Minimal residual disease quantification after 1st phase of induction (applies to all patients) and MRD quantification post-transplant (non-myeloablative conditioned transplant patients only)<br>Grade 3/4 graft versus host diseases (applies to transplant patients only)<br>Death in complete remission<br>Rates of mucositis as reported by Oral Daily Mucositis Score (ODMQ), post-transplant AE data and compliance with delivery of post-transplant methotrexate (applies to palifermin randomisation only)

**Patients:**	826 patients (664 B-cell and 162 T-cell)<br>The initial target overall recruitment was 720 patients (576 B-cell and 144 T-cell) with newly diagnosed, untreated acute lymphoblastic leukaemia with or without evidence of Philadelphia chromosome.<br>Recruitment was extended in December 2016 to replace the first 91 patients (78 B-cell and 13 T-cell) treated prior to an urgent safety measure amending the backbone chemotherapy regimen.<br>Thirteen patients who had misdiagnoses (10 B-cell and 3 T-cell) and two T-cell patients who were Philadelphia positive will not be included in the main analysis population and therefore these patients are also replaced.
**Planned number of sites:**	65
**Target countries:**	United Kingdom
**Treatment Summary:**<br>**(see also trial schema)**	**Randomised patients:**<br>**PHASE 1 & 2**<br>Treatment for all randomised patients will consist of a steroid pre-phase followed by 2 phases of induction therapy during which novel agents will be tested (precursor-B-lineage: 2 randomisation arms termed B1 and B2 and T-lineage ALL 2 randomisation arms termed T1 and T2), as indicated in study aims, above.<br>**After PHASE 2**<br>**A risk assessment will be carried out for each patient based on presenting characteristics, cytogenetics and minimal residual disease analysis**<br>All patients with an HLA-matched sibling donor, regardless of risk stratification, will proceed to allogeneic SCT. Patients aged ≤40 years at study entry will receive a myeloablative conditioning regimen, patients aged ≥41 years at study entry will receive a non-myeloablative conditioning regimen.<br>For all other patients further therapy depends on risk:<br>Standard Risk: Continuing chemotherapy based on a modified UKALL12/ECOG2993 schedule (4 cycles of combination chemotherapy and then standard maintenance therapy).<br>High Risk and aged ≤40 years at time of study entry will be allocated to allogeneic SCT with a matched unrelated donor and receive a myeloablative conditioning regimen (Etoposide-TBI is preferred but Cyclophosphamide-TBI can be used.)<br>High risk and aged ≥41 years at time of study entry will be allocated to allogeneic SCT with a matched unrelated donor and receive a non-myeloablative conditioning regimen (fludarabine-melphalan + alemtuzumab). Intensification with high-dose methotrexate and pegylated asparaginase similar to the UKALL12/ECOG2993 trial will be given in the non-myeloablative conditioned setting.
**Anticipated duration of recruitment (randomised study):**	7.5 years
**Duration of patient follow up (randomised study):**	All patients to be followed up for a minimum of 4.5 years.
**Definition of end of trial:**	Recruitment: 7.5 years<br>Active treatment: 2.5 years when maintenance chemotherapy is given<br>Follow-up: 2 years after the last randomised patient has completed maintenance therapy<br>The end of the follow-up phase signifies the end of the trial.

**Correlative Science:**	1. To determine the relationship between CD20 expression on ALL blasts and response to monoclonal antibody therapy<br>2. To determine whether the administration of an anti-B-cell monoclonal antibody as part of induction therapy for ALL limits the extent anti-asparaginase antibodies formation and promotes asparagine depletion.<br>3. To perform genomic profiling in order to discover and characterise novel prognostic markers and to identify known sub-microscopic copy number alterations (CNA) beyond the resolution of standard diagnostic testing (i.e. cytogenetics and FISH).<br>4. To determine whether the speed at which full donor chimerism is achieved in the T–cell compartment correlates with the level of molecularly determined minimal residual disease.<br>5. To formally assess the late effects of ALL therapy for all patients on the trial, whether they have received chemotherapy alone or an allograft.<br>6. Extraction of constitutional DNA to establish potential risk factors for adult ALL (participation in this analysis is optional)
**Drug Supply:**	1. Rituximab (Mabthera®) – Supplied free of charge by Roche.<br>2. Nelarabine (Atriance®) – Supplied free of charge by Novartis AG<br>3. Pegylated Asparaginase (Oncaspar®) – Supplied to sites at standard list price by Baxalta, now part of Shire<br>All other drugs specified in the protocol must be provided from pharmacy stock at participating sites, see UKALL14 drug supply guidelines for more information.<br><br>NB Palifermin was supplied free of charge until April 2016, when the randomisation was closed.

1.2 Trial schema:

UKALL14 TRIAL SCHEMA for randomised patients – following closure of palifermin randomisation April 2016

***Phase 1 Induction (4 weeks)*: precursor B-cell & T-cell patients (Philadelphia –ve patients only) to receive Pegylated-Asparaginase plus standard phase 1 induction therapy**

Pre B-cell ALL

- B1: Standard Phase 1 Therapy Alone
- B2: Standard Phase 1 Therapy + CD20

T-cell ALL

- Standard Phase 1 Therapy

***Phase 2 Induction (4 weeks)*: precursor B-cell & T-cell patients to receive standard phase 2 induction therapy**

Pre B-cell ALL

- Standard Phase 2 Therapy

T-cell ALL

- T1: Standard Phase 2 Therapy
- T2: Standard Phase 2 Therapy + Nelarabine

PATIENTS IN COMPLETE REMISSION (CR) – Risk Assessment performed on all patients at this time point

SIBLING DONOR

Aged 41 years and over*

Intensification with HD-Methotrexate + pegylated asparaginase ***(Philadelphia –ve patients only to receive pegylated asparaginase)***

To receive allo SCT (sibling), non-myeloablative conditioning with fludarabine-melphalan and

Aged 40 years and under*

To receive allo SCT (sibling),

myeloablative conditioning with Etopside-TBI or

NO SIBLING DONOR

High Risk

Aged 41 years and over*

Intensification with HD-Methotrexate + pegylated asparaginase ***(Philadelphia –ve patients only to receive pegylated asparaginase)***

To receive allo SCT (MUD), non-myeloablative conditioning with

Aged 40 years and under*

To receive allo SCT (MUD),

myeloablative conditioning with

Standard Risk

Continue methotrexate intensification, consolidation and maintenance

***Age at study entry**

2.0 Background

2.1 Introduction

The treatment of children with acute lymphoblastic leukaemia (ALL) is a shining example of the success of combination chemotherapy in curing malignancy. Results of recent trials suggest that up to 90% of children may be cured of their disease. Adult patients with ALL now have a 90% chance of entering first complete remission (CR) with modern chemotherapy. However, most patients still relapse, and leukaemia-free survival with three to seven years of follow-up is only 30-40%. The poor outcome of chemotherapy in adults with ALL as compared to children relates to multiple factors, some of which are known (e.g. adults have a higher incidence of poor prognostic subtypes such as Ph+ /t(9;22)/BCR-ABL ALL and a lower incidence of favourable subtypes such as t(12;21)(p13;q22)/ETV6-RUNX1 and high hyperdipliody) but others, are unknown.

2.2 Induction therapy for ALL

The primary goal of induction therapy is a complete eradication of ALL cells from blood, bone marrow and CNS or other extramedullary sites (when initially involved). This should be achieved in as many patients and as early as possible, and with as few toxic side effects as possible, in order to start rapidly the post-remission consolidation. For Ph- ALL, induction therapy involve three sequential, connected steps, a pre-phase, induction I and induction II with the latter applied regardless of CR after induction I. Many examples of highly effective induction protocols have been reported with CR rates of 90% or more. Because of the complexity of existing induction regimens and the fact that it is already possible to obtain a CR in 90% or more of unselected patients, the evaluation of any new treatment element will also have to evaluate alternative endpoints as early surrogate marker of long-term response. The induction regimen used in this protocol is based on that used in UKALL12/ECOG2993[3] with some modifications, some of which are rooted in changes in practice since the design of UKALL12 and some of which comprise the questions to be asked.

2.3 Steroid for induction of ALL

Corticosteroids are one of the most important drugs in the treatment of ALL, and recent trials in paediatric ALL patients have suggested that the use of dexamethasone, as opposed to prednisolone may improve outcome. This is based on data suggesting that dexamethasone has greater in vitro anti-leukaemia activity than prednisolone, better penetration of the CNS and causes fewer thromboembolic events.[64] Randomised trials have demonstrated improved survival in children receiving dexamethasone as opposed to prednisolone[4], although this has not been shown in every study.[65] Based on these data this trial will substitute a discontinuous schedule of dexamethasone for prednisolone during induction.

2.4 Monoclonal antibodies in induction of precursor B-lineage ALL

2.4.1 Anti CD20 antibody: Rituximab

Rituximab, a chimeric IgG-1 anti-CD20 monoclonal antibody, has found ever expanding uses since its approval by the FDA in 1997. When combined with CHOP chemotherapy in the treatment of diffuse large B-cell lymphoma, the combination of chemotherapy with rituximab was shown to have an overall survival advantage.[66]
Rituximab has been combined with chemotherapy in a wide range of schedules of Lymphoma therapy without any evidence of increased toxicity.

CD20 is expressed on nearly 70% of pre-B ALL cells, although at lower intensity than in non-Hodgkin lymphoma or chronic lymphocytic leukaemia (E. Paietta, personal communication). The ability to combine rituximab with chemotherapy in the treatment of lymphoma and the expression of CD20 in ALL of B-cell precursor-cell type has led to the use of rituximab in the treatment of B-cell precursor ALL in case reports and smaller case series[5,6].

A recent report of 28 patients with adult-Burkitt-type lymphoma and ALL (B-ALL) suggested an advantage with a Hyper-CVAD plus rituximab compared to historical controls treated with hyper CVAD alone. Hyper-CVAD and rituximab have also been combined for treatment of B-cell precursor-ALL patients. The addition of rituximab to Hyper-CVAD appeared to improve disease-free survival in CD20-positive patients compared to historical controls treated with Hyper-CVAD alone[7]. These data are encouraging enough to warrant a randomised trial comparing chemotherapy with or without rituximab in induction for newly diagnosed patients with bcr-abl negative B-cell precursor-ALL. This data has recently been updated with longer follow up at the American Society of Haematology meeting December 2008.

The expression of CD20 and CD22 on ALL blasts varies widely (E. Paietta, personal communication) CD20 is less commonly or less highly expressed on B-ALL blasts than some other B-cell antigens. However, there is accumulating evidence that it is of prognostic significance. No data in ALL - or other tumour types - are available to define the relationship between antigen expression and response and suggest a threshold level for response. Particularly intriguing in this regard is a recent demonstration that the relatively modest expression level of CD20 at diagnosis was often dramatically up-regulated, both in numbers of cells expressing the antigen and levels of expression per cell, following induction chemotherapy. This was shown in vitro to occur on exposure to glucocorticoids, and correlated well with in-vitro rituximab-induced killing[9].
We propose to compare the efficacy and toxicity of induction chemotherapy in ALL alone or in combination with Rituximab.

2.5 Nelarabine for induction of T-cell ALL

Guanine arabinoside (ara-G) was first synthesised in 1964 and subsequently shown to have pre-clinical activity against human T-cell lymphoid malignancies. A pro-drug with increased solubility, known as compound 506U78, was developed. A phase I study was carried out in 93 patients with refractory haematologic malignancies utilizing a daily one-hour intravenous infusion schedule of administration for five days. Doses ranging from 5 to 75 mg/kg/day were given with dose-limiting neurotoxicity encountered at the dose of 75 mg/kg/day. One patient each had grade 3 neurotoxicity at doses of 20 and 60 mg/kg/day. Maximum tolerated doses were defined as 40 mg/kg/day in adults and 60 mg/kg/day in children. To facilitate future phase II studies, dosing was converted to

doses given per m^2 of body surface area during the study and it was projected that 1.2 grams/m^2/day (approximately 30 mg/kg/day in adults and 40 mg/kg/day in children) would be a reasonable phase II dose. Both central and peripheral neurotoxicity were the main toxicities seen and were largely reversible[10] A subsequent phase II study of nelarabine conducted by the Children's Oncology Group (COG) in patients with refractory T-cell malignancies treated four groups of patients: first bone marrow (BM) relapse, second or greater BM relapse, central nervous system (CNS) relapse and extramedullary (EM) relapse. An initial dose of 1.2 grams/m^2/day for five days was chosen, but was de-escalated to 900 mg/m^2/day and then to 650 mg/m2/day because of severe neurotoxicity. The third and fourth groups with CNS and EM relapse were treated with 400 mg/m^2/day to obtain pilot response data with the anticipation that this dose might be utilized in combination with chemotherapy in future trials. Complete plus partial response rates in the four groups were 55% (first BM relapse), 27% (second BM relapse), 33% (CNS relapse) and 14% (EM relapse). Neurological events of grade 3 or greater were seen in 18% of patients [11] The COG has recently reported the safety and feasibility of combining nelarabine at a doses of both 650 and 400 mg/m^2/day for five days with an intensive modified BFM regimen in children with T-cell precursor ALL. A cohort of patients on the study was treated without nelarabine. Neurological and non-neurological toxicities were similar between the two dose levels of nelarabine and those treated without nelarabine. A phase III trial of nelarabine in combination with augmented BFM chemotherapy in consolidation therapy in newly diagnosed intermediate and high-risk children and young adults with T-ALL has recently opened in COG (AALL0434). A study in adults showed approximately 30% response in relapsed and resistant ALL with neurotoxicity being minimal and haematological toxicity being the most common. In the present trial we propose to add a course of nelarabine to induction in a randomised fashion following phase II of induction therapy in patients with T-ALL.

2.6 Role of L-asparaginase

L-asparaginase is arguably one of the most valuable drugs in the treatment of ALL. However, it is associated with numerous toxicities including hepatic dysfunction, pancreatitis and thrombohaemorrhagic complications related to depletion of coagulation factors. An additional complicating feature of the use of L-asparaginase clinically is the development of antibodies to the enzyme that can either result in hypersensitivity reactions (IgE) or via neutralising antibodies (IgG) decrease in enzyme activity with loss of therapeutic efficacy. Neutralizing antibodies developing in the absence of a clinical reaction is known as silent inactivation. When toxicity occurs early in treatment, therapeutic delays are often generated which can compromise the aims of therapy. The appropriate dose, preparation and formulation of L-asparaginase remain unresolved. In paediatric practice, pegylated L-asparaginase (pegylated asparaginase) is less immunogenic and gives the most appropriate pharmacokinetic and pharmacodynamics but evidence that this agent could be properly used in adults was lacking until a recent CALGB phase II study used pegylated asparaginaseas part of a multi-agent regimen. Effective asparaginase depletion was achieved in some adults [15]although increasing age was associated with significantly decreased pegylated asparaginasedoses and less asparagine depletion. Furthermore, there was still a significant number of patients who developed of anti-asparaginase antibodies; this correlated with a less successful asparagine depletion. Within the associated clinical study UKALL14, pegylated asparaginase will be administered to adults for the first time in a large phase 3 setting.

L-asparaginase is prepared from bacterial sources of either Escherichia coli or Erwinia chrysanthemi. E Coli asparagnase is available either as a native asparaginase or conjugated to polyethylene glycol (pegylated asparaginase). Pegylation extends the half-life and lessens the frequency of injections. The use of pegylated asparaginase has been compared to the Ecolab native asparaginase preparation in two randomised trials. In the DFCI 91-01 study, patients were randomised to receive pegylated asparaginase 2500 IU/m^2 every other week for 15 doses or E. coli asparaginase 25,000 IU/m^2 every week for 30 doses in intensification. Event-free survival and toxicities were similar between the two asparaginase arms[17]. A CCG trial compared pegylated asparaginase 2500 IU/m^2 on day 3 of induction to nine doses of native E. coli asparaginase, 6000 IU/m^2 every 3 days in induction and found a lower rate of high titre antibody formation with pegylated asparaginase, but similar rates of adverse events and similar EFS[18]. Recent data from the CALGB has shown that effective asparagine depletion with pegylated asparaginase results in improved outcomes compared with patients not achieving effective asparagine depletion[15].

Based on these and other data, we propose to evaluate the treatment of patients in this study with pegylated asparaginase and ask specific questions about the toxicity and some scientific questions about asparagine depletion and anti-asparaginase antibody formation.

In the case of hypersensitivity to pegylated asparaginase, Erwina asparaginase should be substituted at a dose of 20,000units/m^2 IM (x6 doses) as a replacement for each scheduled dose of pegylated asparaginase. Please see appendix 14 for further guidance.

2.7 Prophylaxis against Central Nervous System Disease in ALL

The presence of leukaemic blasts in the central nervous system (CNS) is a more frequent occurrence in patients with ALL than AML and the importance of prophylactic treatment to prevent the development of progression to the CNS has long been recognised[19]. The results of therapy in patients with CNS involvement at diagnosis in the current MRC UK ALL XII/ECOG E2993 trial have recently been published. Seventy-seven of 1508 (5%) patients had CNS involvement at diagnosis. The incidence is higher in patients with T-cell ALL. Of these 77 patients, 69 (90%) achieved complete remission. This study has demonstrated that, while long-term disease-free survival is attainable in patients who present with CNS involvement, overall survival at five years was inferior at 29% compared to 38% for patients without CNS involvement (p=0.03)[20].

The role of prophylactic cranial irradiation in the era of combined intrathecal and high-dose systemic therapy has been questioned in recent studies with the stated intent of reducing the risk of late sequelae. This has been of particular interest to paediatricians and trials in children have demonstrated that CNS irradiation can be eliminated without worsening overall outcome[22]. In adults several trials have reported CNS recurrence rates less than 10% with the combined use of high dose systemic and intrathecal chemotherapy without the use of cranial irradiation[24-26]. In the UKALL12/E2993 the risk of CNS recurrence was 4%. We propose to discontinue the use of cranial irradiation in the trial proposed here, but will plan to perform interim analyses to detect any increase in CNS recurrence rate.

2.8 Role of Haematopoietic Stem Cell Transplantation (HSCT) in adult ALL

Numerous trials have been conducted incorporating autologous and allogeneic SCT into the treatment algorithm. These trials, while varying in design, have generally demonstrated that allogeneic transplant is more effective than autologous transplant or chemotherapy in either high-risk patients or in all patients treated.

One of the most important analyses was the comparison of the outcome of Ph- patients assigned to HLA-matched sibling SCT compared to patients randomised to autologous SCT or chemotherapy (so-called donor vs. no donor analysis) in UKALL12/E 2993. In a comparison of 389 patients with a donor to 530 patients without a donor, the donor groups had superior EFS (50% versus 41%, p=0.009) and OS (53% versus 45%, p=0.02). A similar statistically significant benefit was seen when the no donor group was restricted to those who were randomised to the chemotherapy arm only. Of importance, this benefit was primarily seen in the standard risk patients (OS 63% for donor versus 51% for no donor patients, p=0.01), but not in high-risk patients (OS 40% versus 36%, p=0.6). The lack of difference in outcomes between donor and no donor patients in the high-risk group were related to a high non-relapse mortality of 39% at two years (20% at two years for the standard risk patients)[27] which in large part was seen in older patients. An additional justification for considering allogeneic SCT in first CR is the dismal outcome of patients who relapse from first CR in the current UKALL12/E2993 trial with an overall survival at 5 years of 7%[28].

Hence allogeneic transplant is currently the treatment of choice for eligible adults in first complete remission (CR1). At present, a group of adults with ALL in whom the risk of relapse is less than the risk of sibling allogeneic SCT cannot be defined. Accordingly, this study continues to propose sibling allogeneic SCT for every eligible patient where a sibling donor is available.

2.8.1 Myeloablative conditioning regimen

The "Stanford" conditioning regimen of high dose etoposide and total body irradiation (TBI) was originally chosen for UKALL12/E2993 as it offered the best published results in SCT for ALL. There are no randomised controlled studies comparing conditioning regimens. Best available evidence is a retrospective analysis of IBMTR data. This suggests no difference in relapse risk or OS between conditioning regimens containing cyclophosphamide and etoposide when patients are transplanted in CR1, while there was an advantage to etoposide and higher doses of TBI in patients transplanted in CR2. There is now considerable experience with the use of the etoposide/TBI regimen among centres carrying out allogeneic SCT for ALL, the appropriate course of action is to continue to use the etoposide-TBI conditioning regimen for this study in patients aged 40 years and under at the time of study entry.

2.8.2 Prevention of oral mucositis – the role of Palifermin

The development of palifermin and demonstration of its efficacy in reducing the incidence and severity of oral mucositis following a TBI-containing conditioning regimen in the autologous setting has generated considerable interest in the BMT community[32]. However, the schedule of administration of palifermin is cumbersome in that it mandates administration of three doses prior to initiation of the conditioning regimen. Palifermin has been tested in the setting of allogeneic BMT in a phase I/II study and shown to be safe. The etoposide-TBI regimen to be

utilised in this study has considerable anti-leukaemic efficacy, but is associated with a 100% incidence of grade 3-4 mucositis. This toxicity has an important consequence for GVHD prophylaxis as most patients cannot receive all 4 planned methotrexate (MTX) doses required for the standard cyclosporin and short course MTX GVHD prophylaxis regimen. Two major studies confirm the importance of MTX dose delivery[34,35] in preventing severe acute GVHD, which is an important cause of mortality that can affect transplant outcome. Preliminary data suggest that a 'collapsed dose' of Palifermin is as effective as the standard administration schedule. There are some phase 2 data and data from murine models, which also suggest the hypothesis that a collapsed schedule may be more effective. A randomised comparison of standard (6 doses) versus 'collapsed' (4 doses) was initially incorporated into the UKALL14 trial in combination with the etoposide-TBI-containing regimen in an attempt to reduce the morbidity and mortality of this intensive regimen. The palifermin randomisation was closed in April 2016 when the surrender of the drug's EU marketing authorisation rendered the question no longer clinically relevant.

2.8.3 Non-myeloablative conditioning regimens in ALL

The current UKALL12/E2993 trial confirmed the benefit of allogeneic SCT in inducing a potent GVL effect, but was associated with unacceptable toxicity in older patients. Non-myeloablative conditioning SCT (RIC-SCT) has allowed the extension of the use of allogeneic SCT to older and infirm patients. There is a paucity of data on the use of RIC-SCT in the treatment of patients with ALL[37,38]. These small studies have, however, demonstrated the feasibility of this approach with preservation of a GVL effect.

Most RIC-SCT conditioning regimens combine a purine nucleoside analogue (most commonly fludarabine) to induce potent T-cell immunosuppression with low dose TBI or an alkylating agent such as cyclophosphamide or melphalan with additional immunosuppression with antithymocyte globulin or alemtuzumab[39]. The fludarabine-melphalan regimen has been extensively used for RIC-SCT for both myeloid and lymphoid malignancies and shown antitumor activity with acceptable toxicity[40-42]. Recent data from the City of Hope National Medical Centre reported on 21 patients primarily consisting of patients with high risk ALL in first or more advanced stages of remission who were not eligible for conventional BMT and received a conditioning regimen of fludarabine and melphalan combined with a matched related (33%) or unrelated (67%) donor allograft. With 17 months of follow-up, the one year cumulative probability of overall or disease-free survival and relapse were 77%, 71% and 8%, respectively. The incidence of acute GVHD, grades II-IV and III-IV, were 50% and 15%, respectively. The 100-day non-relapse mortality was 10%. In the United Kingdom, alemtuzumab has been combined with fludarabine and melphalan to help ensure engraftment of donor cells and reduce the risk of GVHD. These studies have shown low rates of 100 day non-relapse mortality (<15%) with low rates of acute GVHD, grades III-IV, of <10%), but a significant incidence of viral and fungal infections[40-42]. This trial will document the use of a fludarabine-melphalan-alemtuzumab regimen for RIC-SCT with either related or unrelated donors for patients aged 41 years and over at the time of study entry to determine if we can retain a GVL effect in older adults while reducing non-relapse mortality. The cut-off age of 40 years was based on a combination of data from UKALL12/ECOG2993 and informed opinion from the transplant communities in the UK and the USA.

Please note that as patients receiving non-myeloablative conditioning regimens DO NOT receive TBI, there is a need to maintain appropriate CNS directed therapy. These patients will therefore receive 8 x 3 monthly intrathecal

methotexate injections at a dose of 12.5 mg (total dose) for 2 years post-transplant starting at 3 months post-transplant - see section 7.2.11.

2.8.4 The use of unrelated donors

Evidence has accumulated to suggest that risk of MUD allogeneic SCT for patients with ALL is now not much greater than risk of sibling allogeneic SCT[45, 38]). The outcome for patients at high risk of relapse is sufficiently poor that an evaluation of MUD allogeneic SCT is proposed for those patients deemed to be at the highest risk of relapse. In the UKALL12/E2993 trial, only those patients with Ph- ALL were deemed to be at high enough risk for relapse to warrant the risk of MUD allogeneic SCT. In the current study, other high-risk factors confirmed both by data from UKALL12/E2993[46,47] and from other published studies have been introduced. Patients aged ≥41, those with precursor B-cell disease with WBC >30 X 10^9/L or T-cell precursor disease with WBC>100 x 10^9/L) or the presence of high-risk cytogenetic abnormalities; Philidelphia chromosome/t(9;22)(q34;q11)/BCR-ABL1, t(4;11), low hypodiploidy/near triploidy and complex karyotype, will each confer "high-risk".

2.8.5 The source of stem cells

Peripheral blood stem cells have a number of advantages compared to bone marrow in the setting of related donor allogeneic SCT. They result in faster haematopoietic recovery and shorter length of hospitalisation but may also result in a higher incidence of chronic graft versus host disease[48,49] Larger patients (>80 kg) should certainly receive peripheral blood stem cells as first preference. There is no evidence that for patients in first remission that peripheral blood stem cells result in superior survival to bone marrow and therefore no recommendation can be made with regard to stem cell source in the sibling donor setting. When peripheral blood stem cells are used a more prolonged period of immunosuppression for GVHD prophylaxis should be considered and careful monitoring for chronic graft versus host disease should occur.

Controversy exists over the preferred source of stem cells for matched unrelated donor (MUD) allogeneic SCT for ALL due to a retrospective analysis of European registry data, which suggested that survival after MUD allogeneic SCT for ALL may be reduced by the use of PBSC as compared to BM[50]. The survival advantage for BM in this study related to both procedure related mortality and relapse risk. The preferred source of stem cell for MUD allogeneic SCT may be BM, but in the absence of confirmatory prospective data, PBSC remain acceptable.

The UKALL14 TMG has agreed to allow cord blood transplantation (CBT) in patients with MRD positivity after phase 2 or high risk cytogenetics. This is based on a CIBMTR study showing equivalent outcomes between CBT and unrelated donor SCT[67].

CBT should only be performed by centres experienced in this procedure and advice should be sought regarding cord selection where appropriate from the Anthonly Nolan Trust or experienced H&I laboratories.

2.9 Measurement of Minimal Residual Disease (MRD)

Despite the identification of the risk factors described above, many patients with ALL with or without high-risk features still relapse. These relapses presumably occur as a result of minimal residual disease (MRD) not detected

by conventional methods of assessing remission status. The introduction of more sensitive molecular techniques to detect MRD in ALL patients and predict their risk of relapse utilizing clonal immunoglobulin or T-cell receptor gene rearrangements have been very effective. In paediatric ALL, the presence of residual disease at the end of induction chemotherapy or at later time points was a powerful predictor of relapse independent of other risk factors[52]. In adult ALL, the use of these molecular markers has been introduced more recently and also shown to be of prognostic significance. The German Multi-Centre Study Group for adult ALL has recently published data indicating that patients who have a rapid decline in their minimal residual disease within the first month of therapy had a three-year relapse rate of 0%. Another subset of patients who had MRD detectable until week 16 of therapy had a three-year relapse rate of 94%. Patients in between these two groups had an intermediate risk of 47% Measurement of MRD in a subset of patients in the on-going MRC/ECOG UKALL12/E2993 trial has also shown evidence of prognostic significance, especially in the pre-B ALL group. The detection of MRD at the end of the second month of induction therapy appeared to be the most sensitive time point[62]. Measurement of MRD will be incorporated into the trial presented here to help stratify patients with low risk clinical prognostic features into those who are MRD positive at the end of induction therapy and therefore, re-categorised as high risk and now eligible for allogeneic SCT, and those who are MRD negative who will be treated with consolidation and maintenance chemotherapy and not be allocated to allogeneic SCT. Measurement of MRD will be performed in all patients and correlated with outcome.

2.10 'Registration only' sub-study

The UKALL14 B-cell randomisation (standard induction ± Rituximab) closed in July 2017, having reached its recruitment target of 664 patients. From implementation of protocol v11.0 onwards, newly-diagnosed B-cell patients will be given the opportunity to enter an observational 'registration only' sub-study, whereby they will receive standard ALL treatment as per their local clinician's choice, while contributing minimal data and samples (MRD and optional constitutional DNA) to address laboratory aims. 'Registration only' patients will not be required to follow a protocol-defined treatment regimen, and any medication given as part of standard treatment will not be considered as an IMP.
The UKALL14 T-cell randomisation (standard induction ± Nelarabine) is due to close mid 2018 when randomisation reaches its target of 162 patients. Therefore from implementation of protocol v12.0, or completion of the T-cell randomisation (whichever is later), newly-diagnosed T-cell patients will also be given the opportunity to enter the 'registration only' sub-study, to receive standard ALL treatment as per their local clinician's choice while contributing minimal data and samples, as described above.

Please refer to Appendix 1 for full details.

2.11 Summary of study aims

Primary

- To determine if the addition of monoclonal antibody to standard induction chemotherapy results in improved EFS in patients with precursor B-cell ALL (aim 1B).
- To determine if the addition of nelarabine improves outcome for patients with T-cell ALL (aim 1T).

Secondary

- To determine the tolerability of pegylated asparaginase in induction treatment of all patients (aim 2) and to compare anti-asparaginase antibody levels between patients in the 2 randomisation groups from aim 1B (patients with B-lineage ALL only).
- To determine whether risk-adapted introduction of unrelated donor HSCT (myeloablative conditioning in patients ≤40 years old and non-myeloablative conditioning in patients ≥41 years old) results in greater EFS for patients at highest risk of relapse (aim 3).
- To compare 2 schedules of administration (standard vs. 'collapsed') of keratinocyte growth factor (palifermin) for efficacy in preventing the severe mucosal toxicity of etoposide/TBI HSCT conditioning regimen (aim 4 – randomisation closed April 2016 as no longer clinically relevant).
- To formally assess the late effects of ALL therapy for all patients on the trial, whether they have received chemotherapy alone or an allograft. To identify and describe some of the adverse physical and psychosocial consequences of the disease and its treatment.

3.0 Selection of Trial Sites/Site Investigators

3.1 Trial Site selection

In this protocol, trial 'site' refers to the hospital where trial-related activities are conducted.

Sites should be able to comply with:

- Trial treatments, sample collection procedures and follow up schedules
- Research Governance Framework and the Medicines for Human Use (clinical trials) Act (SI 2004/1031 and all amendments)
- The trial protocol
- Data collection requirements.

Each site should also have:

- Haematology Multidisciplinary Team (MDT), which must include a leukaemia specialist
- Transplant centre or a relationship with a referring transplant centre to whom they are prepared to refer every patient for a donor search at diagnosis.
- Transplant centres must be JACIE accredited or working towards JACIE accreditation.

3.2 Selection of Principal Investigator and other investigators at sites

Each site must have an appropriate Principal Investigator (PI) i.e. a health care professional authorised by the site, ethics committee and regulatory authority to lead and coordinate the work of the trial on behalf of the site. Other investigators at site wishing to participate in the trial must be trained and approved by the PI. All investigators must be medical doctors and have experience of treating acute lymphoblastic leukaemia.

3.2.1 Training requirements for site staff

All site staff must be appropriately qualified by education, training and experience to perform the trial related duties allocated to them, which must be recorded on the site delegation log.

CVs for all staff must be kept up-to-date, signed and dated and copies held in the Investigator Site File (ISF). An up-to-date, signed copy of the CV for the PI must be forwarded to UCL CTC upon request.

GCP training is required for all staff responsible for trial activities. The frequency of repeat training may be dictated by the requirements of their employing institution, or 2 yearly where the institution has no policy, and more frequently when there have been updates to the legal or regulatory requirements for the conduct of clinical trials.

3.3 Site set up

The following documentation must be submitted by the site to UCL CTC prior to a site being activated by the UCL CTC trial team:

- All relevant institution approvals (including local NHS permission)
- UKALL14 Site Registration Form and Investigator Registration Forms

- A completed Site Delegation Log that is signed and dated by the PI
- A copy of the PI's current CV that is signed and dated

Please note: Non-transplant sites will not be activated by UCL CTC until their referring transplant centre has approval.

The UCL CTC trial team will ensure that, for UK sites:

- If the site was not included in the original CSP application, the Part C is updated and the R&D form is resubmitted to CSP (who will notify the lead CLRN of the new site)
- An SSI form is transferred to the site via IRAS
- If the site was not included on the original REC application, a substantial amendment is submitted to and approved by the REC
- If the site was not included on the original CTA application, the CTA is updated and the MHRA notified at the next substantial amendment to the MHRA.

In addition, the following agreements must be in place:

- A signed Clinical Trial Site Agreement (CTSA) between the Sponsor and the relevant institution (usually a NHS Trust).

3.3.1 Site initiation

All sites will be required to have a site initiation session before recruitment can begin. Site initiation will take the form of either a site visit or a teleconference involving the trial coordinator and site staff involved in the trial. The exact nature of the site initiation will be agreed between the CTC and site staff.

3.3.2 Site activation (UCL CTC responsibility)

Once the UCL CTC trial team has received all required documentation and the site has been initiated, a site activation letter will be issued to the PI, at which point the site may start to approach patients.

Once the site has been activated by UCL CTC, the PI is responsible for ensuring:

- adherence to the most recent version of the protocol;
- all relevant site staff are trained in the protocol requirements;
- appropriate recruitment and medical care of patients in the trial;
- timely completion and return of CRFs (including assessment of all adverse events);
- prompt notification and assessment of all serious adverse events;
- that the site has facilities to provide **24 hour medical advice** for trial patients.

Please note: Non-transplant sites will not be activated by UCL CTC until their referring transplant centre has approval.

4.0 Informed consent

Sites are responsible for assessing a patient's capacity to give informed consent.

Sites must ensure that all patients have been given the current approved version of the patient information sheet, are fully informed about the trial and have confirmed their willingness to take part in the trial by signing the current approved consent form.

Sites must assess a patient's ability to understand verbal and written information in English and whether or not an interpreter would be required to ensure fully informed consent. If a patient requires an interpreter and none is available, the patient should not be considered for the trial.

The PI, or, where delegated by the PI, other appropriately trained site staff, are required to provide a full explanation of the trial and all relevant treatment options to each patient prior to trial entry. During these discussions the current approved patient information sheet for the trial should be discussed with the patient. A minimum of twenty four hours should be allowed for the patient to consider and discuss participation in the trial. However if this is not possible (eg. patient needs to start treatment urgently), patients may consent on the same day as being given the patient information sheet, provided the member of staff taking consent is satisfied that the patient understands the trial and its implications. A member of the research team at the hospital should then have a further discussion with the patient in the following days to confirm their ongoing willingness to participate in the trial. This should be recorded in the source data. Written informed consent on the current approved version of the consent form for the trial must be obtained before any trial-specific procedures are conducted. The discussion and consent process must be documented in the patient notes.
Non-UK sites will need to consent patients to the trial according to local practice and regulatory and/or ethical requirements.

Site staff are responsible for:

- checking that the current approved version of the patient information sheet and consent form are used
- checking information on the consent form is complete and legible
- checking that the patient has completed/initialled all relevant sections and signed and dated the form
- checking that an appropriate member of staff has countersigned and dated the consent form to confirm that they provided information to the patient
- checking that an appropriate member of staff has made dated entries in the patient's medical notes relating to the informed consent process (i.e. information given, consent signed, etc.)
- giving the patient a copy of their signed consent form, patient information sheet and patient contact card
- documenting and providing details of the consent process to UCL CTC
- following registration/randomisation: adding the patient trial number to all copies of the consent forms, which should be filed in the patient's medical notes and investigator site file.

The right of the patient to refuse to participate in the trial without giving reasons must be respected. All patients are free to withdraw at any time from the protocol treatment without giving reasons and without prejudicing

further treatment. Any patient's withdrawal of consent from the trial must be explicitly documented in the source documents and UCL CTC informed. Also refer to section 14 (Withdrawal of Patients).

5.0 Selection of Patients

5.1 Pre-registration & pre-randomisation evaluation (study entry)

The following assessments and procedures are required prior to registration (please note that there are further assessments and procedures required prior to transplant – see section 5.4):

- Full medical history (including family history of cancer) & physical examination
  - Including cardiac history, any history of mental health issues and employment status pre-ALL diagnosis (baseline measurements for the late effects assessment at 2 years follow up in section 8.3)
- Height, Weight & BSA
- Assessment of performance status (ECOG)
- Full blood counts & biochemistry
- Hepatitis B serology
- Hepatitis C serology
- HIV testing is advised as standard of care, but is not mandated
- Bone marrow aspirate & trephine
- Pregnancy test for all women of childbearing age
- Confirmation of disease diagnosis from bone marrow aspirate (or peripheral blood where there is high presenting WCC) using immunophenotyping/flow cytometry
- Cytogenetic, FISH and molecular genetic analysis on a pre-treatment bone marrow is essential to
  - (a) confirm the presence or absence of the Philadephia (Ph) chromosome \ t(9;22)(q34;q11) \ *BCR-ABL1*. Where patient is Ph pos, imatinib will be administered with the therapy.
  - (b) identify the following high risk abnormalities (refer to Appendix 7 for cytogenetic definitions and detection strategy;
    - Philadephia chromosome \ t(9;22)(q34;q11) \ *BCR-ABL1*.
      - These patients will need imatinib added to therapy
    - t(4;11)(q21;q23) / *MLL-AF4*
      - NB *AFF1* was previously known as *AF4* and *MLLT2*
    - low hypodiploidy / near-triploidy (Ho-Tr)
    - complex karyotype (five or more chromosomal abnormalities)
  - These tests are to be performed by the local or regional cytogenetic/genetic laboratory and a copy of the report of all diagnostic and relapse cytogenetic, FISH and genetic testing must be sent to the cytogenetic coordinating centre - the Leukaemia Research Cytogentics Group (LRCG). See Appendix 7 for contact details.
- The cytogenetic co-ordinating centre (LRCG) may request left-over fixed cell suspension, DNA, RNA or other material from the local genetics laboratory or the treating clinician to undertake further cytogenetic, molecular cytogenetic and genetic testing (including but not restricted to FISH, array CGH and RT-PCR) to refine the definition of known abnormalities and characterise novel subgroups. All these additional tests will be performed with the full knowledge of the CI and clinical coordinators.

- Lumbar puncture is not required at diagnosis except in the case of suspected central nervous system involvement. Otherwise, it should be avoided (in case of traumatic puncture and CNS seeding) until the first dose of intrathecal methotrexate is due, at which time blasts should have been cleared from the peripheral blood. The first lumbar puncture should always be given by the most experienced operator available, to reduce the incidence of traumatic taps.
- A buccal swab will be taken for constitutional DNA extraction (if patient has consented).

Please note, as detailed in section 8.2.1 and Appendix 8:

	**Specimens for local assessment**	**Specimens for correlative studies to be sent to central laboratory (see appx. 8 for address)**
**At Diagnosis**	Cytogenetics/molecular assessment of BCR-ABL and MLL on bone marrow. A copy of the results should be sent to the Leukaemia Research Cytogenetics Group (LRCG) at Newcastle University. (Please refer to Appendix 7 for details of the LRCG)<br>Tissue typing of patient and any siblings to be carried out. Donor search to be initiated if no matched sibling donor available.	Bone marrow 3-5ml in EDTA (OR peripheral blood 30-50ml in EDTA if bone marrow not available).<br>BCR-ABL status will also be checked.<br>Buccal swab (if patient has consented; see section 9.7)

5.2 Patient Eligibility for Study Entry

For eligibility for transplantation, see section 5.5.

All patients must be eligible for the trial in accordance with the inclusion and exclusion criteria.

5.2.1 Patient Inclusion Criteria (study entry)

a) Subjects must be aged ≥ 25 and ≤ 65 years old with acute lymphoblastic leukaemia OR ≥ 19 and ≤ 65 years old with Philadelphia Chromosome positive acute lymphoblastic leukaemia.
b) Newly diagnosed, previously untreated ALL (a steroid pre-phase of 5-7 days is required and can be started prior to registration)
c) Written informed consent

5.2.2 Patient Exclusion Criteria (study entry)

a) Known HIV infection
b) Hepatitis B infection (defined as positive HBsAg and/or HBcAb). Antibodies to Hep B surface antigen only is acceptable.*
c) Hepatitis C infection (antibodies against hepatitis C or a PCR evaluation which is positive for hepatitis C DNA)*
d) Pregnant or lactating women
e) Blast transformation of CML
f) Mature B-cell leukemia i.e. Burkitt's lymphoma t(8;14)(q24;q32) and variant c-myc translocations e.g. t(2;8)(p12;q24), t(8;22)(q24;q11)

*Patients with evidence of past infection with HBV or HCV are eligible for trial registration/randomisation at the discretion of the treating clinician and should be managed in accordance with local guidelines.

The table below contains a brief outline for interpretation of HBV serology:

HBsAg	HBsAb	HBcAb	HBV DNA	Interpretation	Eligible Y/N
-	+	-	-	Vaccinated	Y
-	+	+	-	Past infection;<br>low risk reactivation	Y<br>at discretion of PI
-	-<br>(<10IU/L)	+	-	Past infection;<br>higher risk reactivation[1]	Y<br>at discretion of PI
-	+/-	+	+	Occult infection	N
+	Any results			Chronic carrier or infection	N

HBsAg = Hepatitis B surface antigen; HBsAb = Hepatitis B surface antibody; HBcAb = Hepatitis B core antibody

1. Law JK, Ho JK, Hoskins PJ, Erb SR, Steinbrecher UP, Yoshida EM. Fatal reactivation of hepatitis B post-chemotherapy for lymphoma in a hepatitis B surface antigen-negative, hepatitis B core antibody-positive patient: potential implications for future prophylaxis recommendations. Leuk Lymphoma 2005;46:1085-1089

5.2.3 Management of patients with poor organ function at study entry

In order for the protocol to reflect 'real-life' management of ALL, patients with poor organ function at diagnosis are not excluded, but extra care should be taken with their management. If patients present with poor renal or hepatic function, relationship to the presenting condition should be sought; imaging should be performed and liver biopsy could be considered, to seek involvement by ALL. A steroid pre-phase is particularly necessary in such patients to gain clinical improvement and normalisation of renal and/or liver function prior to starting study chemotherapy. In the case of persistently abnormal renal or hepatic function at start of protocol therapy, dose adjustments to the non-IMP chemotherapy drugs should be made according to appendix 5 and in conjunction with local network guidelines. Dose modifications should be recorded on the CRF. Dose modification for IMPs, where necessary, are also indicated in appendix 5. If a patient presents with prior history of poor cardiac function, clinically indicated tests should be performed at site and UCL CTC should be contacted to discuss treatment options within the trial.

5.2.4 Pregnancy, Lactation & Birth Control

Pegylated Asparaginase

The risks to an embryo or foetus from exposure to Pegylated Asparaginase are currently unknown.

Rituximab

The risks to the human embryo or foetus from exposure to Rituximab are currently unknown. Developmental toxicity studies performed in cynomolgus monkeys revealed no evidence of embryotoxicity in utero. New born offspring of maternal animals exposed to Rituximab were noted to have depleted B-Cell populations during the post natal phase.

Nelarabine

The risks to the human embryo or foetus from exposure to Nelarabine are currently unknown.
Studies in animals have shown reproductive toxicity including malformations. The potential risk in humans is unknown, however, exposure during pregnancy will likely lead to anomalies and malformations of the

foetus.

Pregnancy Testing

All women of childbearing potential who are at risk of becoming pregnant must undergo a pregnancy test before study entry.

A woman of childbearing potential is a sexually mature woman (i.e. any female who has experienced menstrual bleeding) who has not:

- undergone a hysterectomy or bilateral oophorectomy/salpingectomy
- been postmenopausal for 24 consecutive months (i.e. who has had menses at any time in the preceding 24 consecutive months without an alternative medical cause)

Contraceptive Advice

Due to insufficient data on the effects of trial treatment during pregnancy and lactation, randomised patients must agree to use one of the following acceptable methods of contraception whilst on treatment and for 12 months following completion of treatment.

Acceptable methods of effective contraception for this trial are:

- Established use of oral, injected or implanted hormonal methods of contraception
    - Since an indirect interaction between oral contraceptives and pegylated asparaginase cannot be ruled out, the use of oral contraception is not considered an acceptable method of contraception whilst on pegylated asparaginase treatment.
- Placement of an intrauterine device (IUD) or intrauterine system (IUS).
- Barrier methods of contraception: condom or occlusive cap (diaphragm or cervical/vault caps) with spermicidal foam/gel/film/cream/suppository). The use of barrier contraceptives should always be supplemented with the use of a spermicide. The following should be noted:
    - Failure rates indicate that, when used alone, the diaphragm or condom are **not** highly effective forms of contraception. Therefore the use of additional spermicides does confer additional theoretical contraceptive protection.
    - However, spermicides alone are inefficient at preventing pregnancy when the whole ejaculate is spilled. Therefore, spermicides are not a barrier method of contraception and must not be used alone.
- Male sterilisation (with appropriate post-vasectomy documentation of the absence of sperm in the ejaculate). For female patients, the vasectomised male partners must be the sole partner for that patient. Please note that sterilisation is not usually regarded as completely reliable enough on its own to ensure that pregnancy can never occur.
- Absolute and continuous abstinence: When this is in line with the preferred and usual lifestyle of the patient. Please note that periodic abstinence (e.g. calendar, ovulation, symptothermal, post-ovulation methods) and withdrawal are not acceptable methods of contraception.

The method(s) of contraception used must be stated in the patient medical notes.

If a randomised female patient or the female partner of a randomised male trial patient becomes pregnant between the start of trial treatment and 12 months post completion of trial treatment, UCL CTC must be informed immediately (See section 12.6 for details on the reporting procedure).

5.3 Registration & Randomisation Procedures

Patients will be registered to the trial at study entry, and subsequently registered to either consolidation/ maintenance or transplant following completion of induction therapy.

There are two randomisations in the trial – one for pre-B-cell patients and the other for T-cell patients. **Each patient will undergo one randomisation only, at study entry.** All randomisations will be performed at UCL CTC upon submission of the relevant form by sites.

Sites will be informed by UCL CTC of the randomisation group as follows:

Table 5.3 – UKALL14 Randomisations

Randomisation	Randomisation Timepoint (protocol section)	Patient Group	Randomisation group	Treatment involved	Treatment timepoint	Treatment Protocol section
**B**	Study entry (5.4)	Precursor B-lineage	**B1**	Standard Phase 1 induction therapy alone		7.2.3b
			**B2**	+ Rituximab	to be given alongside phase 1 induction therapy	
**T**	Study entry (5.4)	T-lineage	**T1**	Standard Phase 2 induction therapy alone		7.2.5b
			**T2**	+ Nelarabine	to be given once counts have recovered following Phase 2 induction therapy* (withhold randomised drug if current evidence of grade 2 or higher CNS toxicity)	

*Neurological adverse events with the use of NELARABINE

Severe neurological events have been reported with the use of Nelarabine. These events have included altered mental states including severe somnolence, central nervous system effects including convulsions, and peripheral neuropathy ranging from numbness and paresthesias to motor weakness and paralysis. There have also been reports of events associated with demyelination, and ascending peripheral neuropathies similar in appearance to Guillain-Barré Syndrome.

Full recovery from these events has not always occurred with cessation of Nelarabine. Therefore, close monitoring for neurological events is strongly recommended, and Nelarabine must be discontinued at the first sign of neurological events of NCI CTCAE Grade 2 or greater.

5.3.1 Registration & Randomisations (B & T) at study entry

a. Please see section 5.3.4 for details of registration to transplant.

b. Patient registration & randomisation will be performed centrally at the UCL CTC and must be performed prior to commencement of any trial treatment (a steroid pre-phase of 5-7 days is required and can be started prior to study entry).

c. Pre-treatment evaluations should be carried out at sites as detailed in section 5.1.

d. A registration form must be fully completed and faxed to UCL CTC and will be used to confirm patient eligibility at UCL CTC. N.B. If the site is unable to fax, registration forms may be sent by email. If emailing forms, patient identifiable information from the form (e.g. NHS number, day and month of birth) must be redacted before it is emailed to ctc.ukall14@ucl.ac.uk. The identifiable information must be provided to UCL CTC via telephone so that UCL CTC can transcribe this information on to the form. The un-redacted form must then be posted to UCL CTC, and a copy kept in the patient file at site.

e. Initial randomisations (performed via minimisation) (B and T) will be stratified on sex, age (≤40, ≥41 years old) and WBC ≥30 x 10^9/L (precursor-B), WBC ≥100 x 10^9/L (T-lineage)

f. A trial number and treatment allocation will be assigned and details added to the form. UCL CTC will fax/email confirmation of the patient's inclusion in the trial, their trial number and treatment allocation (either B1 or B2, T1 or T2, see table 5.3) to the main contact. Trial numbers will take the following format:

    i. B-cell randomised patients: 14-1-xxx
    ii. T-cell randomised patients: 14-2-xxx.

Once a patient has been registered onto the trial they must be provided with the following:

- A copy of their signed consent form (if not given at time of consent).
- A patient contact card. Site on call contact details for out of hours medical care must be added to this card and patients informed to carry this with them at all times while on the trial.

Registration & Randomisation fax number:	+44 (0)20 7679 9861
Office hours:	09:00 to 17:00 (UK Time)<br>Monday to Friday (excluding public holidays)

5.3.2 Pre-transplant evaluation

Please contact the Transplant co-ordinator (see front of protocol) if you have any queries about the eligibility of a patient for transplant or any other transplant queries.

1. Medical history including allergies, previous chemotherapy, prior radiotherapy, hormonal or immunotherapy and response to treatment, end-organ toxicity and infections.
2. Physical examination.
3. ECOG performance status (Appendix 11).
4. Karnofsky performance status (Appendix 16).
5. Comorbidity Index (Appendix 10).
6. Full blood count with differential
7. Biochemistry (to include LDH). Liver and renal function should be assessed as per local practice.
8. Urinalysis.
9. Clotting screen including PT/APTT.
10. Pregnancy test (urine) as clinically indicated.
11. Microbial titres (CMV, HIV I+2, HBsAg, HBcAb, HC, HTLV 1+2, EBV, VZV, TPHA, toxoplasma +/- HSv). Syphilis serology also required.
12. Bone marrow aspirate, and trephine biopsy if local practice (The bone marrow sample taken at the end of Phase II will be acceptable here as long as there are no significant delays. Please discuss with the transplant co-ordinator for advice if you anticipate a delay.)

13. Cardiac function (to be assessed as per local practice).
14. Pulmonary function (to be assessed as per local practice).
15. DNA specimen from patient and donor should be screened for informative markers for subsequent lineage-specific chimerism studies. Residual DNA should be stored in accordance with the local chimerism laboratory's routine practice.

Please note – as detailed in section 8.2.1:

	**Specimens for local assessment**
**Pre-transplant (non-myeloablative transplant patients only)**	As per local practice.<br><br>Peripheral blood or buccal swab from <u>donor and recipient</u> to be sent to the local chimerism lab for assessment of mini-satellite regions.<br>An anonymised copy of the results must be sent promptly to UCL CTC.

5.3.3 **Patient eligibility for transplant**

5.3.3.1 **Inclusion criteria (transplant)**

a. Completion of Phase 1 and Phase 2 treatment within the trial

b. The following subgroups of patients will proceed to transplant:
   i. Any patient with an HLA compatible sibling donor
   ii. High Risk patients with a 8/8 molecularly matched donor at HLA-A,B,C and DR (see 5.6.2)
   iii. Patients with high risk disease in whom no 8/8 donor match is available can proceed to transplant with a 7/8 matched unrelated donor OR umbilical cord unit(s) in the following circumstances:
      - high risk cytogenetics
      - positive minimal residual disease (MRD) after phase 2 induction

Cord blood units selected for transplant should meet the selection criteria of the UK national cord trials. There is a UK national cord selection committee who can be consulted for advice (ctc.cordselection@ucl.ac.uk). These patients can also be discussed with the trial transplant coordinator, Professor David Marks (07798 732090 or David.Marks@uhbristol.nhs.uk).

5.3.3.2 Eligibility for high-risk arm – unrelated donor stem cell transplantation:

Any one of the factors below makes the patient high-risk:

a. Age ≥41 years
b. WBC ≥30 x 10^9/L (precursor-B), ≥100 x 10^9/L (T-lineage)
c. Cytogenetics – any one or more of the abnormalities below
   i. t(4;11)(q21;q23)/*MLL-AF4*
   ii. Low hypodiploidy/near triploidy (30-39 chromosomes / 60-78 chromosomes
   iii. Complex karyotype (five or more chromosomal abnormalities) Philadelphia chromosome t(9;22)
   iv. (q34;q11)/BCR-ABL1 (detected by cytogenetic or molecular methods)
d. High Risk Minimal Residual Disease (MRD) post phase 2 of induction.

NB. Patients aged ≥41 at time of study entry will be given non-myeloablative conditioning and patients aged ≤40 at study entry will be given myeloablative conditioning unless agreed in advance with the UKALL14 transplant coordinator.

5.3.3.3 Exclusion criteria (transplant)

a. Relapsed disease
b. Standard risk patients without a sibling donor (these patients will continue chemotherapy consolidation and maintenance)

5.3.4 Procedure for Registration to either consolidation/maintenance or transplant

- Registration to either consolidation/maintenance or transplant will be performed centrally at the UCL CTC.
- Pre-transplant evaluations should be carried out as detailed in section 5.5.
- A post-induction treatment allocation form and *either* a consolidation/maintenance registration form *or* a transplant registration form must be fully completed and faxed (or emailed with patient identifiable information removed) to UCL CTC as soon as possible after completion of phase 2 induction. However, this should not be submitted until the site knows whether the patient is planned to proceed to transplant or not (i.e. donor status and fitness for transplant confirmed).
- UCL CTC will fax/email confirmation to the main contact at site of either:
- Patient not eligible to proceed to transplant and will be treated using consolidation and maintenance as detailed in sections 7.2.8 and 7.2.9.
- Patient may proceed to transplant as detailed in section 7.2.10.

Registration fax number:	+44 (0)20 7679 9861
Office hours:	09:00 to 17:00
	(UK Time)
	Monday to Friday (excluding public holidays)

5.3.5 Transfer to a new site for Transplant

If a patient is transferred to another hospital for their transplant, the procedures outlined in section 14.3 (Moving) must be followed in order that UCL CTC has an up to date record of which hospital is responsible for the patient's care and data submission.

- A centre transfer form must be completed and submitted to UCL CTC
- Any data pertaining to trial visits up to the point of transfer must be completed and submitted to UCL CTC
- A copy of the patient's CRFs up to the point of transfer must be provided to the transplant centre.

The same process should be followed when patients are discharged from their transplant centre back to their original hospital.

6.0 Trial drug supply

For full instructions for drug ordering, delivery and labelling for the UKALL14 trial, please consult the UKALL14 Drug Supply Guidelines.

7.0 Trial Treatments

7.1 Study drugs

7.1.1 Summary of IMPs to be evaluated

For the purpose of this protocol, the IMPs are:

1. Rituximab (Mabthera®) - supplied free of charge by Roche Products Ltd.
2. Pegylated asparaginase (Oncaspar®) - supplied to sites at standard list price by Baxalta, now part of Shire.
3. Nelarabine (Atriance®) - supplied free of charge by Novartis AG.
4. Palifermin (Kepivance®) was supplied free of charge by Swedish Orphan Biovitrum until April 2016, when the randomisation was closed.

All other drugs specified in the protocol are standard treatment for this disease and are not IMPs. These must be provided from pharmacy stock at the participating sites.

Full details of all IMPs and supporting medications are supplied in Appendix 3.
For full instructions for drug ordering, delivery and labelling for the UKALL14 trial, please consult the UKALL14 Drug Supply Guidelines.

7.1.2 Summary of non-IMPs

All backbone chemotherapy and transplant conditioning drugs administered to randomised patients are regarded as non-IMPs for the purpose of the trial. For avoidance of doubt, the non-IMPs are:

- Dexamethasone
- Vincristine
- Daunorubicin
- Methotrexate
- Cyclophosphamide
- Mercaptopurine
- Cytarabine
- Fludarabine
- Alemtuzumab
- Etoposide
- Prednisolone
- Imatinib
- Melphalan.

7.2 Treatment Schedule

Please refer to sections 7.2.2 to 7.2.11 for detailed treatment schedules for each treatment phase.
Please note the following important points regarding UKALL14 treatment:

Oncaspar

- Oncaspar must not be administered to Philadelphia positive patients.
- Patients aged ≥41 must not be administered Oncaspar on day 4 of induction phase 1

Nelarabine

- Nelarabine must not be administered to patients who have ≥ grade 2 CNS toxicity at the end of phase 2 induction

Non-IMP 'backbone' chemotherapy

- Every effort should be made to give non-IMP chemotherapy as outlined in the protocol, however it is noted that delays, reductions or substitutions may be required in the light of patients' clinical condition.
- If a scheduled intrathecal dose is missed for clinical reasons, it should be made up later in the cycle. It is anticipated that the correct number of intrathecals is given, as per the table below:

Treatment phase	Chemo only patients	Non-myeloablative allograft patients	Myeloablative allograft patients
Induction 1	1	1	1
Induction 2	4	4	4
Intensification	0	0	0
Consolidation	5	N/A	N/A
Maintenance	8	N/A	N/A
Post non-myeloablative allograft	N/A	8	N/A
TOTAL	18	13	5

- If patients are unable to tolerate intrathecal methotrexate, cytarabine may be substituted.
- If further advice is required regarding substitutions, delays and making up missed doses, the TMG are happy to discuss on a case-by-case basis.

Transplant conditioning

The transplant conditioning regimens outlined in the protocol must be used unless agreed **in advance** on a case-by-case basis with Prof David Marks, UKALL14 Transplant Coordinator.

7.2.1 Recommended supportive care:

Local policies may be followed with regard to prophylaxis, however, we recommend the following:
Allopurinol should be started 24 hours prior to induction chemotherapy and should be continued for a minimum of 5 days. Rasburicase should be considered as an alternative to allopurinol if the white cell count is high i.e >100 x 10^9/L or the patient has bulky disease eg. large mediastinal mass or elevated urate at diagnosis.
All patients need prophylaxis against HSV and VZV reactivation. It is recommended that patients are given aciclovir 200mg bd throughout therapy although local policies may be followed.
All patients need prophylaxis against PCP from day zero of induction. The recommended PCP prophylaxis is co-trimoxazole 960mg bd for 2 days each week, avoiding the day that methotrexate is given when the patient is on maintenance therapy. In the event of the patient being allergic to co-trimoxazole, local policies should be followed but alternative prophylactic agents include nebulised pentamidine or dapsone.
Antifungal prophylaxis is mandatory for all patients on ALL therapy from the time of induction. Azoles must be avoided when the patient is on vincristine. There is no clear evidence to suggest which anti-fungal prophylaxis regimen should be used in this situation but one option is to give AmBisome® 7mg/kg weekly. Local policies may be followed. Azoles can be used safely after phase 1 of induction, however they may affect plasma levels of

imatinib, in which case dose reductions can be made as necessary. This should be discussed with the local PI and/or treating clinician, and documented in the relevant CRF.
Antifungal prophylaxis is not generally required when a patient is on maintenance therapy unless that patient is deemed to be high risk for fungal disease.

The use of granulocyte colony stimulating factor (G-CSF) is strongly recommended for all patients to hasten neutrophil recovery following induction phases 1 and 2. It can be given as per local policy.

Chugai Pharma UK will provide a 25% retrospective reimbursement in stock for rHuG-CSF (Lenograstim) used within the study period.
Please contact medicalaffairs@chugai-pharm.co.uk with regards to making a claim.

Granocyte is available at NHS contract prices through AAH Hospital Service Telephone 0845 607 6699.

Management of thrombosis and haemostasis during L-asparaginase therapy
The coagulation screen will inevitably be deranged during L-asparaginase therapy, but as bleeding is rarely a clinical problem there is no need to treat these changes. There is poor understanding of the coagulation abnormalities associated with the use of L-asparaginase. Previous studies have shown that fresh frozen plasma administration does not improve abnormal coagulation parameters.

Fibrinogen levels are often low. Emerging (as yet unpublished) evidence suggests that administration of fibrinogen concentrates to correct the low fibrinogen can enhance the risk of thrombosis in non-bleeding states. In summary, as per any other abnormal coagulation screen in a patient without bleeding, there is no indication to give any plasma products to correct the changes.

The main clinical problem in patients on L-asparaginase is the high risk of venous thromboembolism (VTE). As per NICE thromboprophylaxis guidelines[92], we recommend the use of thromboprophylaxis – usually low molecular weight heparin – in unwell hospitalised adults who have reduced mobility, provided the platelet count is > 50 x 10^9/l.

Prophylactic LMWH should be stopped at least 12 hours prior to an invasive procedure.

There is some evidence that maintaining AT levels above 60% is associated with fewer venous thromboses related to L-asparaginase therapy and possibly a better clinical outcome[54]. Antithrombin supplementation is usually achieved by giving antithrombin (AT) concentrates, which are expensive. Currently there is insufficient evidence to make it a requirement within the protocol for thromboprophylaxis, but evidence is stronger for administration in the presence of acute VTE (see below).

Thrombosis and central venous catheters:

- The greatest risk of thrombosis (CNS and central line related) is in early induction. The presence of a central catheter considerably increases this risk and there is some evidence that survival is affected. The risk is reduced when dexamethasone is used compared to prednisolone.
- Centres should consider delaying indwelling central venous catheterization until the end of phase I induction.

Management of thrombosis

VTE occurs in 10-15% of patients receiving L-asparaginase treatment. Acute VTE should be treated with full dose low molecular weight heparin (LMWH) (or unfractionated heparin in those with renal failure or where fast reversal is required e.g. high risk of bleeding or pre-procedure) and the platelet count can be maintained over 50 x 10^9/l with platelet infusions.

Treatment doses of LMWH should be stopped 24 hours prior to a procedure (4 hours for un-fractionated). Unfractionated heparin requires regular APTT monitoring to maintain APTT of 2-2.5. LMWH does not require monitoring as it has predictable pharmacokinetics.

Since heparin works as an anticoagulant by potentiating AT 10,000 fold, some patients will be relatively resistant to heparin due to reduced plasma levels of AT caused by L-asparaginase. Ideally AT levels should be monitored and supplemented in those being treated for acute VTE, to obtain maximal effect of the heparin in this critical situation.

We do not recommend that future L-asparaginase must be with held in those with acute VTE as long as appropriate anticoagulation and AT supplementation can be given.

If expert haemostatic advice is required for an individual patient on protocol, it can be obtained from Professor Beverley Hunt or Dr Charlotte Bradbury who can be contacted via the trials office or by emailing beverley.hunt@gstt.nhs.uk or c.bradbury@bristol.ac.uk. Please copy any correspondence to ctc.ukall14@ucl.ac.uk.

VTEs are regarded as an AE of Special Interest for the trial (see section 12.3 'AE of Special Interest').

Methotrexate encephalopathy management:

- Methotrexate encephalopathy presents with fits, focal neurological deficit or impaired consciousness and occurs within one day to about 3 weeks of exposure to intrathecal methotrexate. Full recovery is usual.
- Other causes of CNS events should be considered such as sagittal sinus thrombosis or central nervous system involvement with ALL.
- Intrathecal methotrexate should be discontinued whilst the patient is also receiving cytarabine systemically.
- Re-challenge is possible without recurrence but if recurrence happens, the intrathecal regimen should be changed to cytarabine 50mg with steroid cover as per local policy.

7.2.2 Steroid pre-phase

All patients should be treated with a steroid pre-phase of 5-7 days. Study entry is still permissible if the steroid pre-phase has started prior to study registration. The steroid pre-phase consists of Dexamethasone 6mg/m^2/d, PO for 5 to 7 days.

7.2.3 Phase 1 induction

<u>Phase 1 induction, weeks 1-4</u>

To be given to all patients regardless of phenotype. Patients with B precursor lineage disease will be randomised to receive either Rituximab or no antibody (randomisation groups B1 and B2, see table 5.3).

Patients with Philadelphia positive disease should also receive continuous daily Imatinib, PO, starting at 400mg, aiming to escalate to 600mg within 2 weeks, if tolerated.

Please note: Patients with Philadelphia Positive ALL should NOT be given pegylated asparaginase.

<u>For schedules see Tables 7.2.3a & 7.2.3b below</u>

Table 7.2.3a – Phase I induction

Drug	Dose	Route of administration	Day 1	Day 2	Day 3	Day 4	Day 5	Day 6	Day 7	Day 8	Day 9	Day 10	Day 11	Day 12	Day 13	Day 14	Day 15	Day 16	Day 17	Day 18	Day 19	Day 20	Day 21	Day 22	Day 23	Day 24	Day 25	Day 26	Day 27	Day 28
Daunorubicin	**30 mg/m²**	IV	1							8							15							22						
Vincristine***	**1.4mg/m²**<br>**Max 2mg**	IV	1							8							15							22						
Dexamethasone**	**10mg/m²**	PO	1	2	3	4				8	9	10	11				15	16	17	18										
Pegylated Asparaginase*.<br>**Philadelphia Positive patients** should NOT be given Pegylated Asparaginase	**1000IU/m²**	IV				4<br>*														18										
Methotrexate****	**12.5mg**	Intrathecal														14														
Imatinib - Patients with **Philadelphia positive disease** should also receive continuous daily Imatinib, PO, starting at 400mg, aiming to escalate to 600mg within 2 weeks, if tolerated. This should be continued until transplant wherever possible			1	2	3	4	5	6	7	8	9	10	11	12	13	14	15	16	17	18	19	20	21	22	23	24	25	26	27	28

****Omit Day 4 Pegylated Asparaginase for Philadelphia Negative patients aged ≥41.** These patients should only receive Day 18 Pegylated Asparaginase.*

Please note: Patients with Philadelphia positive ALL should NOT be given pegylated asparaginase.

Because of the risk of severe liver toxicity with pegylated asparaginase, liver function tests should be performed regularly while patients are being treated with this drug.

***Dexamethasone should be capped at 20mg for larger patients*

****Do not give azoles as antifungal prophylaxis within 72 hours before or after vincristine.*

Notes on lumbar puncture and treatment of established CNS disease:

***** Timing of Intrathecal therapy can be moved +/- 3 days to allow administration on specified lists as per local and national guidance.*

In the case of traumatic lumbar puncture (>10 red blood cells per microlitre), patients should be treated as having CNS disease IF they still have blasts within the peripheral blood at the time of occurrence or have blasts in the CSF. In this case and in the case where there is existing evidence of established CNS disease, intrathecal therapy with methotrexate should be escalated to twice per week and given at this frequency until the cytospin is clear of blasts. Such patients should also receive cranial irradiation, prior to consolidation, if they are not going to receive myeloablative allogeneic transplant.

Table 7.2.3b – B-lineage antibody randomisation during Phase I induction

**ANTIBODIES SCHEDULED BELOW ONLY TO BE GIVEN TO PRECURSOR-B-LINEAGE PATIENTS DEPENDING ON RANDOMISATION**																														
Drug	Dose	Route of administration	Day 1	Day 2	Day 3	Day 4	Day 5	Day 6	Day 7	Day 8	Day 9	Day 10	Day 11	Day 12	Day 13	Day 14	Day 15	Day 16	Day 17	Day 18	Day 19	Day 20	Day 21	Day 22	Day 23	Day 24	Day 25	Day 26	Day 27	Day 28
Rituximab plus phase 1; Arm B2																														
Rituximab*	**375mg/m^2**	IV			3							10							17							24				

**Rituximab to be given to patients with precursor-B-lineage ALL according to randomisation after chemotherapy.*

Please note as detailed in section 8.2.1 and Appendix 8:

	**Specimens for local assessment**	**Specimens for correlative studies to be sent to central laboratory (see appx. 8 for address)**
**During Phase 1 therapy**	As per local practice	5ml peripheral blood in a serum tube (for Asparaginase activity assay and anti-Asparaginase antibodies) taken on d3 or d4 and on d18 (immediately prior to doses 1 and 2 of pegylated asparaginase for patients aged ≤40), or taken on d18 (prior to pegylated asparaginase administration) and d32 in patients aged ≥41. **Not applicable to Philadelphia positive disease**<br><br>On the same day as the pre-asparaginase sample is taken, a 4.5ml sodium citrate tube should be filled (to the line) to collect plasma. Spin the tube @ 2000G for 5 mins, aliquot serum into 5 eppendorfs and store locally at –80°C if possible or if not, -20°C. (This is an optional sample, to be done if resources allow).

7.2.4 End of phase 1 induction

Following recovery from phase 1 therapy (neutrophils >0.75 x 10^9/l and platelets >75 x 10^9/l), remission should be confirmed by morphological bone marrow examination (a specimen must be sent centrally for MRD examination - see section 8.2.1 and Appendix 8 for full details). This is the absolute minimum count recovery needed for adequate assessment of response and of MRD. Hence the bone marrow aspirate to assess MRD may be postponed for a few more days after reaching this level and can be carried out once the patient has been admitted to begin the next course of therapy, to make sure there is adequate cellularity for the end of phase assessment in order to confirm CR with count recovery. **The bone marrow aspirate must be done by day 35* at the latest.** Please indicate the peripheral blood counts on the form when you submit specimens for central analysis.

However, progression to phase 2 should not be delayed more than a few days once haematopoietic recovery has occurred.

If the patient is not in CR at the end of phase 1, swift progression to phase 2 treatment is indicated.

Please note as detailed in section 8.2.1 and Appendix 8:

	**Specimens for local assessment**	**Specimens for correlative studies to be sent to central laboratory (see appx. 8 for address)**
**At recovery post Phase 1 ***	Bone marrow aspirate for remission assessment locally. Same day local FBC to determine peripheral count.	Bone marrow aspirate : 3-5ml in EDTA for MRD assessment<br>(IgH/TCR rearrangements for Ph-ALL, BCR-ABL for Ph+ALL)<br>To be sent to UCL Cancer Institute

* If count recovery has not been achieved by day 35, the bone marrow aspirate should still be done to check whether non-recovery is due to residual disease. If the day 35 marrow is simply hypocellular with no recovering haematopoiesis or signs of relapsed or residual ALL, it is appropriate to wait a week and repeat the marrow as clinically indicated and to send any subsequent, more cellular specimens for MRD analysis.

7.2.5 Phase 2 induction

Phase 2 induction, weeks 5-8 (please see section 7.2.4 for recovery pre-phase 2)

To be given to all patients regardless of phenotype. Patients with T-lineage ALL will be randomised to receive either Nelarabine as an additional course, following phase 2 or no additional treatment.

Patients with Philadelphia positive ALL should also receive continuous daily Imatinib, PO, starting at 400mg, aiming to escalate to 600mg within 2 weeks, if tolerated.

Table 7.2.5a – Phase 2 Induction

Drug	Dose	Route of administration	Day 1	Day 2	Day 3	Day 4	Day 5	Day 6	Day 7	Day 8	Day 9	Day 10	Day 11	Day 12	Day 13	Day 14	Day 15	Day 16	Day 17	Day 18	Day 19	Day 20	Day 21	Day 22	Day 23	Day 24	Day 25	Day 26	Day 27	Day 28
Cyclophosphamide*	1000 mg/m^2	IV	1														15													
Cytarabine#	75mg/m^2	IV		2	3	4	5				9	10	11	12				16	17	18	19				23	24	25	26		
Mercaptopurine	60mg/m^2	PO	1	2	3	4	5	6	7	8	9	10	11	12	13	14	15	16	17	18	19	20	21	22	23	24	25	26	27	28
Methotrexate#	12.5mg	Intrathecal	1							8							15							22						
Imatinib - Patients with **Philadelphia positive disease** should also receive continuous daily Imatinib, PO, starting at 400mg, aiming to escalate to 600mg within 2 weeks, if tolerated. This should be continued until transplant wherever possible			1	2	3	4	5	6	7	8	9	10	11	12	13	14	15	16	17	18	19	20	21	22	23	24	25	26	27	28

# Timing of Intrathecal therapy can be moved +/- 3 days to allow administration on specified lists as per local and national guidance. Likewise, timing of the cytarabine blocks can be scheduled so that they can take place during the week as long as the full doses are given.

** Cyclophosphamide 1000mg/m^2 IV over 20-30 minutes on days 1 and 15. Give 125 mls/m^2/hour of Dextrose/ Saline for 30 minutes before cyclophosphamide and for 3.5 hours afterwards ie 4 hours in total. Do not add potassium. Mesna is not needed.'*

Table 7.2.5b – Nelarabine schedule for T-lineage patients following phase 2 induction

- Only for patients with T-lineage disease randomised to Nelarabine
- If a patient has grade 2 or greater Central Nervous System (CNS) toxicity at the end of phase 2 induction, nelarabine **must not be given**, and the patient should proceed to the next scheduled phase of treatment.
- Bone marrow sample to assess MRD at the end of phase 2 should be taken prior to administration of nelarabine
- Day 1 of Nelarabine treatment should be given immediately after count recovery following phase 2 induction (neutrophils >0.75 x 10^9/l and platelets >75 x 10^9/l), and must be no earlier than day 29.

Drug	Dose	Route of administration	Day 1	Day 2	Day 3	Day 4	Day 5	Day 6	Day 7	Day 8	Day 9	Day 10	Day 11	Day 12	Day 13	Day 14	Day 15	Day 16	Day 17	Day 18	Day 19	Day 20	Day 21
Phase 2 plus Nelarabine: Arm T2																							
Nelarabine	1.5g/m^2	IV	1		3		5																

Neurological adverse events

Severe neurological events have been reported with the use of Nelarabine. These events have included altered mental states including severe somnolence, central nervous system effects including convulsions, and peripheral neuropathy ranging from numbness and paresthesias to motor weakness and paralysis. There have also been reports of events associated with demyelination, and ascending peripheral neuropathies similar in appearance to Guillain-Barré Syndrome.

Full recovery from these events has not always occurred with cessation of Nelarabine. Therefore, close monitoring for neurological events is stongly recommended, and Nelarabine must be discontinued at the first sign of neurological events of CTCAE Grade 2 or greater.

7.2.6 End of phase 2 induction

Following recovery from phase 2 therapy (neutrophils >0.75 x 10^9/l and platelets >75 x 10^9/l), remission should be confirmed by morphological bone marrow examination (please don't forget to send a specimen centrally for MRD examination - see section 8.2.1 and Appendix 8 for full details). This is the absolute minimum count recovery needed for adequate assessment of response and of MRD. BM to assess MRD at the end of phase 2 should be carried out as soon as counts have recovered sufficiently to give an adequate specimen. The sample should be taken **before** administration of nelarabine. If the patient has no high risk factors at diagnosis, the MRD result is the only risk-stratifying factor, so due consideration must be given to making an extremely timely evaluation as soon as bone marrow has recovered. Please indicate the peripheral blood counts on the form when you submit specimens for central analysis.

However, progression to intensification or bone marrow transplant should be as swift as possible.

<u>If the patient is not in CR at the end of phase 2, protocol therapy ceases. Please refer to Section 14.0. Advice on non-protocol therapy can be obtained from the CI or one of the clinical coordinators (contact details at front of protocol).</u>

Please note as detailed in section 8.2.1 and Appendix 8:

	**Specimens for local assessment**	**Specimens for correlative studies to be sent to central laboratory (see appx. 8 for address)**
**At recovery post Phase 2**	Bone marrow aspirate for remission assessment locally. Same day local FBC to determine peripheral count.	Bone marrow aspirate: 3-5ml in EDTA for MRD assessment<br>(IgH/TCR rearrangements for Ph-ALL, BCR-ABL for Ph+ALL)<br>To be sent to UCL Cancer Institute

7.2.7 Intensification/Central nervous system prophylaxis

N.B This may be omitted if a myeloablative transplant is to be carried out but if there are delays in transplant start (>3 weeks following recovery from phase 2 induction), the patient should continue with per-protocol intensification. If there are still delays in donor procurement following intensification, the patient *should not be left without any anti-leukaemia therapy*. Depending on the projected duration of delay, either 2 monthly cycles of interim maintenance therapy should be given (as per maintenance phase of this protocol, with vincristine and steroid and an intrathecal MTX given each month) OR if anticipated delay is longer than 2 months, patients should instead receive the first cycle of consolidation therapy.

The initial creatinine clearance before starting methotrexate should ideally be >100 mls/minute. Dose reductions must be made if the Cr Cl is <80mls/min.

Treatment to begin upon recovery from induction phase 2.

Patients with Philadelphia positive disease should also receive continuous daily Imatinib, PO, starting at 400mg, aiming to escalate to 600mg within 2 weeks, if tolerated.

Please note: Patients with Philadelphia positive ALL should NOT be given pegylated asparaginase.

<u>For schedule see Tables 7.2.7 below</u>

Table 7.2.7 – Intensification/CNS prophylaxis

Drug	Dose	Route of administration	Day 1	Day 2	Day 3	Day 4	Day 5	Day 6	Day 7	Day 8	Day 9	Day 10	Day 11	Day 12	Day 13	Day 14	Day 15	Day 16	Day 17	Day 18	Day 19	Day 20	Day 21	Day 22	Day 23	Day 24	Day 25	Day 26	Day 27	Day 28
Methotrexate*	3g/m^2	IV	1														15													
Pegylated Asparaginase** **Philadelphia Positive ALL should NOT receive Pegylated Asaparaginase**	1000 IU/m^2	IV		2														16												
Imatinib - Patients with **Philadelphia positive disease** should also receive continuous daily Imatinib, PO, starting at 400mg, aiming to escalate to 600mg within 2 weeks, if tolerated. This should be continued until transplant wherever possible			1	2	3	4	5	6	7	8	9	10	11	12	13	14	15	16	17	18	19	20	21	22	23	24	25	26	27	28

Please consult* ***appendix 15* *for full guidance on administration of High Dose MTX – (please note: after treatment with methotrexate, folinic acid rescue must be given. Please see Appendix 15 for details).*

** Patients with **Philadelphia positive ALL should NOT be given pegylated asparaginase.** *Because of the risk of severe liver toxicity with pegylated asparaginase, liver function tests should be performed regularly while patients are being treated with this drug.*

Please note as detailed in section 8.2.1 and Appendix 8:

	**Specimens for local assessment**	**Specimens for correlative studies to be sent to central laboratory (see appx. 8 for address)**
**During intensification:**	As per local practice	5ml peripheral blood in a serum tube (for Asparaginase activity assay and anti-Asparaginase antibodies) taken on d2 and d16 of intensification (immediately prior to doses 3 and 4 of pegylated asparaginase)<br>On the same day as the pre-asparaginase sample is taken, a 4.5 ml sodium citrate tube should be filled (to the line) to collect plasma. Spin the tube @ 2000G for 5 mins, aliquot serum into 5 eppendorfs and store locally at –80°C if possible or if not, -20°C. (This is an optional sample, to be done if resources allow).

7.2.8 Consolidation therapy

To be given to patients not eligible for transplantation. The first cycle of consolidation therapy should begin after intensification, when neutrophils >0.75 x 10^9/L and platelet >75 x 10^9/L (Please see tables 7.2.8 a-d for details). For patients with central nervous system involvement, cranial irradition will be given before consolidation begins. Maintenance therapy with 6-Mercaptopurine should be given throughout the period of CNS irradiation. In the event of cytopenias, 6-Mercaptopurine therapy should be reduced or omitted rather than radiotherapy being delayed. The dose of thiopurine should not be increased as per the maintenance protocol but should be continued at 75mg/m^2 in the absence of cytopenias.

Table 7.2.8a - Cycle 1 Consolidation – to begin after intensification , when neutrophils >0.75 x 10^9/L and platelets >75 x 10^9/L.

Drug	**Dose**	Route of administration	Day 1	Day 2	Day 3	Day 4	Day 5	Day 6	Day 7
Cytarabine	**75mg/m^2**	IV	1	2	3	4	5		
Etoposide	**100mg/m^2**	IV	1	2	3	4	5		
Pegylated-Asparaginase. **Patients with Philadelphia Positive ALL** should NOT be given Pegylated Asparaginase	**1000 IU/m^2**	IV					5		
Methotrexate#	**12.5 mg**	Intrathecal	1						
Imatinib - Patients with **Philadelphia positive disease** should also receive continuous daily Imatinib, PO, starting at 400mg, aiming to escalate to 600mg within 2 weeks, if tolerated.			1	2	3	4	5	6	7

# Timing of Intrathecal therapy can be moved +/- 3 days to allow administration on specified lists as per local and national guidance. Likewise, timing of the cytarabine blocks can be scheduled so that they can take place during the week as long as the full doses are given.

Please note: Patients with Philadelphia positive ALL should NOT be given pegylated asparaginase.

Because of the risk of severe liver toxicity with pegylated asparaginase, liver function tests should be performed regularly while patients are being treated with this drug.

Table 7.2.8b - Cycle 2 Consolidation – to commence 3 weeks from day one cycle 1 or when neturophils are >0.75 x 10^9/l and platelets >75 x 10^9/l

Drug	Dose	Route of administration	Day 1	Day 2	Day 3	Day 4	Day 5	Day 6	Day 7
Cytarabine	**75mg/m^2**	IV	1	2	3	4	5		
Etoposide	**100mg/m^2**	IV	1	2	3	4	5		
Methotrexate	**12.5 mg**	Intrathecal	1						
Imatinib - Patients with **Philadelphia positive disease** should also receive continuous daily Imatinib, PO, starting at 400mg, aiming to escalate to 600mg within 2 weeks, if tolerated.			1	2	3	4	5	6	7

Table 7.2.8ci – Cycle 3 (DAYS 1-28) - Consolidation/Delayed intensification - to commence 3 weeks from day one cycle 2 or when neutrophil count >0.75 x 10^9/L and platelets >75 x 10^9/l

DAYS 1-28

Drug	Dose	Route of administration	Day 1	Day 2	Day 3	Day 4	Day 5	Day 6	Day 7	Day 8	Day 9	Day 10	Day 11	Day 12	Day 13	Day 14	Day 15	Day 16	Day 17	Day 18	Day 19	Day 20	Day 21	Day 22	Day 23	Day 24	Day 25	Day 26	Day 27	Day 28
Daunorubicin	25mg/m^2	IV	1							8							15							22						
Vincristine	1.4mg/m^2 (Max 2mg)	IV	1							8							15							22						
Pegylated Asparaginase** **Philadelphia Positive ALL should NOT receive Pegylated Asaparaginase**	1000 IU/m^2	IV				4																								
Dexamethasone*	10 mg/m^2	PO	1	2	3	4				8	9	10	11				15	16	17	18				22	23	24	25			
Methotrexate	12.5 mg	Intrathecal		2															17											
Imatinib - Patients with **Philadelphia positive disease** should also receive continuous daily Imatinib, PO, starting at 400mg, aiming to escalate to 600mg within 2 weeks, if tolerated.			1	2	3	4	5	6	7	8	9	10	11	12	13	14	15	16	17	18	19	20	21	22	23	24	25	26	27	28

**Dexamethasone should be capped at 20mg for larger patients.*

** **Please note: Patients with Philadelphia positive ALL should NOT be given pegylated asparaginase.**

Because of the risk of severe liver toxicity with pegylated asparaginase, liver function tests should be performed regularly while patients are being treated with this drug.

This cycle runs from day 1 to day 42 inclusive (i.e. 6 weeks). Patients should have ANC >0.75x10^9/L and platelets of >75x10^9/L to start and have recovered again to this level from before the 29th day of therapy is started. Once begun, therapy is not interrupted for myelosuppression alone. Any serious infection, such as Varicella, pneumocystis pneumonia, or neutropenia with fever, and presumed or proven infection, warrants chemotherapy interruption at any time during this block. Before the patient completes day 29-42 (table 7.2.8cii), i.e. before the d29 cyclophosphamide, the counts should be ANC >0.75x10^9/L and platelets >75x10^9/L.

Table 7.2.8cii – Cycle 3 (DAYS 29-42) – Consolidation/Delayed intensification - to commence 3 weeks and 29 days from day one cycle 2 or when neutrophil count >0.75 x 10^9/L and platelets >75 x 10^9/l

DAYS 29-42

Drug	Dose	Route of administration	Day 29*	Day 30	Day 31	Day 32	Day 33	Day 34	Day 35	Day 36	Day 37	Day 38	Day 39	Day 40	Day 41	Day 42
Cyclophosphamide	**1000 mg/m²**	IV	29													
Cytarabine	**75mg/m²**	IV		30	31	32	33				37	38	39	40		
Mercaptopurine	**60mg/m²**	PO	29	30	31	32	33	34	35	36	37	38	39	40	41	42
Imatinib - Patients with **Philadelphia positive disease** should also receive continuous daily Imatinib, PO, starting at 400mg, aiming to escalate to 600mg within 2 weeks, if tolerated.			29	30	31	32	33	34	35	36	37	38	39	40	41	42

*Before the patient completes day 29-42 (table 7.2.8cii), i.e. before the d29 cyclophosphamide, the counts should be ANC >0.75x10^9/L and platelets >75x10^9/L.

Table 7.2.8d - Cycle 4 – Consolidation: Identical to Cycle 2, and will begin when neutrophils >0.75 x 10^9/L and platelets >75 x 10^9/l

Drug	Dose	Route of administration	Day -1	Day 1	Day 2	Day 3	Day 4	Day 5	Day 6	Day 7
Cytarabine	**75mg/m^2**	IV		1	2	3	4	5		
Etoposide	**100mg/m^2**	IV		1	2	3	4	5		
Methotrexate	**12.5mg**	Intrathecal		1						
Imatinib - Patients with **Philadelphia positive disease** should also receive continuous daily Imatinib, PO, starting at 400mg, aiming to escalate to 600mg within 2 weeks, if tolerated.				1	2	3	4	5	6	7

7.2.9 Maintenance therapy (non-transplant patients only)

To start as soon as neutrophils are >0.75 x 10^9/l and platelets are >75 x 10^9/l following consolidation 4 and to continue for 2 full years.

<u>Patients with Philadelphia positive disease should continue with daily Imatinib throughout maintenance.</u>

Table 7.2.9 – Maintenance therapy

Drug	Dose	Route of administration	Frequency
Vincristine	1.4 mg/m^2 (max 2 mg/dose)	IV	every 3 months
Prednisolone	60 mg/m^2	PO	5 days every 3 months
Mercaptopurine*	75 mg/m^2	PO	Daily
Methotrexate*	20 mg/m^2	PO or IV	once per week (not to be given on the same day as co-trimoxazole)
Intrathecal therapy during maintenance (to be given once neutrophils reach 0.75 x 10^9/l and platelets reach 75 x 10^9/l)			
Methotrexate	12.5mg	Intrathecal	Every 3 months

Dosing of maintenance therapy should be adjusted to maintain the neutrophil count between 0.75 and 1.5 x 10^9/l and platelet count between 75 and 150 x 10^9/l.

*Dose of MP and MTX should be alterered in 25% increments or decrements to achieve the above counts. eg if neutrophils > 1.5 x 10^9/l, increase 6-MP dose by 25%. If neutrophils remain > 1.5 x 10^9/l after 4 weeks, increase MTX by 25% etc. There are no maximum doses of MP and MTX. If neutrophils fall below 0.75 x 10^9/l, reduce both drugs by 50%, if neutrophils fall below 0.5 x 10^9/l, stop maintenance and restart at 100% when neutrophils > 0.75 x 10^9/l. Similar adjustments need to be made for the platelet count to maintain above counts.

Maintenance should not be interrupted unnecessarily but, with the exception of IT methotrexate, if doses are omitted for cytopaenias or infectious complications, they do not need to be made up with additional doses later.

Co-trimoxazole (960mg bd, twice per week, not on the same day as the weekly oral MTX) and aciclovir (200mg bd) prophylaxis against PCP and HSV/VZV reactivation should be given throughout maintenance. Local practice may be followed regarding the aciclovir dose if necessary.

If cytopaenias occur and maintenance is halted, consideration should be given to stopping the co-trimoxazole if blood counts do not recover within 2-3 weeks. Doses of mercaptopurine and MTX should not be compromised in order to permit continuation of co-trimoxazole.

Alternative prophylaxis against PCP should be given, for example monthly nebulised pentamindine, or oral dapsone.

7.2.10 Transplant conditioning regimens

Please see appendix 9 for guidance on donor peripheral blood stem cell collection & return.

In accordance with the trial schema (section 1.2), all randomised patients aged 40 years and under at study entry with a sibling donor or high risk disease should be considered for a myeloablative transplant. The risks and benefits of transplant should be discussed with the patient by a transplant clinician. If a patient aged 40 or under with a sibling donor but no high risk features elects not to proceed to transplant, they will be permitted to proceed to intensification and consolidation and will remain on trial. The decision should be clearly documented in the patient notes and on the post-induction treatment allocation form. If, for any other reason, the protocol defined transplant conditioning regimen is not considered appropriate for a particular patient, the treatment plan should be discussed and agreed with Professor David Marks, UKALL14 Transplant Coordinator. Any deviations from the trial protocol should be recorded on the case report forms and documented in the patient's notes.

Agreed variations to transplant procedure will not constitute withdrawal from trial treatment and a full set of transplant case report forms must be completed and submitted. Follow up will be carried out as for patients who received per-protocol transplants.

Please note – as detailed in section 8.2.1:

	**Specimens for local assessment**
**Pre-transplant (non-myeloablative transplant patients only)**	As per local practice.<br><br>Peripheral blood or buccal swab from <u>donor and recipient</u> to be sent to the local chimerism lab for assessment of mini-satellite regions.<br>An anonymised copy of the results must be sent promptly to UCL CTC.

7.2.10.1 Myeloablative conditioning regimen

- The TBI-etoposide regimen outlined in table 7.2.10a below is strongly recommended for patients aged ≤40 years at study entry who are eligible for myeloablative transplantation.
- At the discretion of the transplant centres, cyclophosphamide 60mg/kg on days -3 and -2 is acceptable as an alternative to etoposide (see table 7.2.10b).
- Local practice for the *scheduling* of the conditioning regimen may be followed.
- The protocol defined transplant conditioning regimens should be used wherever possible, and the use of any other regimens must be discussed with, and agreed by, the UKALL14 Transplant Coordinator, Professor David Marks, before proceeding.
- TBI dose should not be less than 13.2Gy (1320 cGy).
- T-cell depletion is not recommended for unrelated donor SCT. Where T-cell depletion is deemed necessary by individual centres, '*in-vivo*' Alemtuzumab is recommended at 30mg IV days -2 and -1. The dose given should be documented on the relevant CRF.

Table 7.2.10a – Myeloablative conditioning regimen – preferred option: Etoposide

Drug	Dose	Route of administration	Day –7	Day –6	Day –5	Day –4	Day –3	Day –2	Day –1	Day 0
Fractionated TBI	**Total dose 1320 cGY in 8 fractions**		-7	-6	-5	-4				
Etoposide	**60 mg/kg**	IV					-3			
Haematopoetic stem cell infusion										0

Suggested GvHD prophylaxis:
Ciclosporin, initially at a total dose of 3 mg/kg/day IV from d-1 to +28 (see section 7.2.10.5 for details of switching to oral and weaning) – or as per local protocol
IV Methotrexate 15mg/m^2 d+1 & 10mg/m^2 d+3, +6 & +11 (see section 7.2.10.5 for dose adjustments)

Table 7.2.10b – Myeloablative conditioning regimen – alternative option: Cyclophosphamide

Drug	Dose	Route of administration	Day –7	Day –6	Day –5	Day –4	Day –3	Day –2	Day –1	Day 0
Fractionated TBI	**Total dose 1320 cGY in 8 fractions**		-7	-6	-5	-4				
Cyclophosphamide	**60 mg/kg**	IV					-3	-2		
Haematopoetic stem cell infusion										0

Suggested GvHD prophylaxis:
Ciclosporin, initially at a total dose of 3 mg/kg/day IV from d-1 to +28 (see section 7.2.10.5 for details of switching to oral and weaning) – or as per local protocol
IV Methotrexate 15mg/m^2 d+1 & 10mg/m^2 d+3, +6 & +11 (see section 7.2.10.5 for dose adjustments)

7.2.10.2 Non-myeloablative conditioning therapy

The regimen outlined in table 7.2.10e below should be used for patients aged ≥41 at study entry who are eligible for non-myeloablative transplantation. Any variations to the regimen must be agreed with the study Transplant Coordinator, Professor David Marks, before proceeding.

Table 7.2.10e – Non-myeloablative conditioning regimen schedule

Drug	Dose	Route of administration	Day -7	Day -6	Day -5	Day-4	Day-3	Day-2	Day-1	Day 0	3-monthly post Tx
Fludarabine	**30 mg/m²**	IV over 1 hour	-7	-6	-5	-4	-3				
Melphalan	**140 mg/m²**	IV							-1		
For recipients of unrelated donor allografts:								-2	-1		
Alemtuzumab*	**30 mg**	IV									
For recipients of sibling allografts:									-1		
Alemtuzumab*	**30 mg**	IV									
Haematopoetic stem cell infusion		IV								0	
Methotrexate	**12.5mg**	IT									X

**Pre-medication with steroids prior to Alemtuzumab is strongly recommended, in addition to antihistamines and paracetamol*

Suggested GvHD prophylaxis:
Ciclosporin, initially at a total dose of 3 mg/kg/day IV from d-1 to +28 (see section 7.2.10.5 for details of switching to oral and weaning) – or as per local protocol

Please note that as patients receiving non-myeloblative conditioning regimens DO NOT receive TBI there is a need to maintain appropriate CNS directed therapy. These patients will therefore receive 8 x 3-monthly intrathecal methotrexate injections at a dose of 12.5 mg, for 2 years post-transplant starting at 3 months post-transplant - see section 7.2.11.

Please note, as detailed in section 8.2.1 and appendix 8:

	**Specimens for local assessment**	**Specimens for correlative studies to be sent to central laboratories (see appx. 8 for addresses)**
**Post-transplant (non-myeloablative only):**	As per local practice.<br><br>Peripheral blood in EDTA to be sent to the local chimerism lab. An anonymised copy of the results must be sent promptly to UCL CTC.<br>First sample to be sent 3 months post-transplant. To be repeated every 3 months until 2 years post-transplant or until relapse.	3-5 ml bone marrow aspirate in EDTA for MRD assessment.<br>(to be sent to UCL Cancer Institute)<br>First sample to be sent 3 months post-transplant. To be repeated every 3 months until 2 years post-transplant or until relapse.

7.2.10.3 Supportive care during and after transplant

Investigating transplant centres should use local protocols with regard to anti-emetic and anti-infective prophylaxis.

7.2.10.4 Imatinib after transplant

There is no concrete evidence about whether, when, for how long and under what circumstances to give imatinib post allograft. Published data show that when given within the first 2-3 months it is toxic and poorly tolerated and most patients cannot continue with it. Unpublished data from a small randomised study from GMALL in which patients were randomised to receive imatinib either 'prophylactically' (i.e. all patients given the drug beginning at 3 month, regardless of bcr-abl status) or 'pre-emptively' (i.e. only if bcr-abl positive) showed no difference in outcome between the two arms. Some of the patients who were bcr-abl post allograft became negative after starting imatinib. Unsurprisingly, those with persistent bcr-abl from an early time point post allograft did poorly, regardless of whether they received imatinib or not. Those who became positive at later time-points had a better outcome and some had a long term good outcome when starting imatinib at the time of re-appearance of bcr-abl. In summary, it would not be a good idea to start imatinib until at least 3 months post allograft. Thereafter, physicians can pursue a policy of giving it to all of their patients or giving it only to those who are (or become) bcr-abl positive. Both strategies appear to produce the same long term outcome. Both require adherence to regular bcr-abl quantification for best results. There are no data whatsoever to indicate how long to best continue the drug. In UKALL12, it was given within the trial for 2 years, but we don’t have data on how many continued at physicians discretion after the two year period nor do we have data on the benefit of continuing in the long term. Hence it might make more sense pragmatically to only start the drug in the presence of a positive bcr-abl signal because a) there are possible side effects and drug interactions and b) once started in a bcr-abl negative patient, stopping decisions will be potentially problematic.

7.2.10.5 Graft versus host disease prophylaxis

Ciclosporin

Ciclosporin will be given as follows: total dose of 3 mg/kg/day I.V. from day -1 to +28. (Local practices for GVHD prophylaxis may be followed). The dose will be adjusted to maintain a therapeutic blood level and the patient will be switched to oral ciclosporin once clinically indicated. Ciclosporin will tapered from 2 months and discontinued at 3 months in the absence of GVHD.

Methotrexate

Short course methotrexate is given as per the original Seattle protocol to patients receiving a myeloablative transplant. Please note; MTX is NOT needed for the non-myeloablative regimen

The dosing is as follows:

15 mg/m^2 day 1 and 10 mg/m^2 days 3, 6 and 11

We strongly advise that 4 doses of methotrexate should be given as lower doses of methotrexate may result in increased incidences of acute and chronic GVHD.

Moderate mucositis should not prevent full methotrexate dosing.

Dose reductions should occur in renal impairment:

CrCl 61-80 mL/minute: reduce dose to 75% of usual dose

CrCl 51-60 mL/minute: reduce dose to 50% of usual dose

CrCl < 50 mL/minute: no methotrexate

Investigators are permitted to give omitted doses of methotrexate when the renal impairment has resolved and can discuss this with the transplant coordinator of the trial.

Dosage adjustment in hepatic impairment:

Bilirubin 50-75 umol/l or AST >180 units: administer 50% of usual dose

Bilirubin >75 umol/l : no methotrexate

Patients at particular risk of progressive renal or hepatic impairment may require greater reductions in dosage. Discussion with the transplant coordinator of the trial is advised.

Folinic acid rescue to be given as per local policy.

7.2.10.6 Prophylaxis against infectious diseases

All patients should receive prophylaxis against infection with HSV, VZV, fungal organisms and PCP according to local guidelines. CMV monitoring by PCR should be undertaken.

7.2.10.7 Donor lymphocyte infusions (DLI)

Disease and chimerism status will be assessed in all patients as per schedule in correlative studies section post non-myeloablative allogeneic transplant. If needed, DLI will be given in escalating doses at 3-monthly intervals.

Indications for DLI:

1. mixed chimerism
2. continued or progressive minimal residual disease

DLI is to be given at the following doses, starting at 6 months post-transplant:

6 months: 1 x 10^6 T-cells/kg
9 months: 3 x 10^6 T-cells/kg
12 months: 1 x 10^7 T-cells/kg
15 months: 3 x 10^7 T-cells/kg
18 months: 1 x 10^8 T-cells/kg

The chimerism status will be assessed 2-3 months after each infusion as per the 'Chimerism-triggered DLI' section below and section 8.2.1. No further infusions will be given if both remission of the immune phenomena and full donor chimerism is achieved. However, if the patient fails to achieve this after the final dose of donor lymphocytes (1 x 10^8 T-cells/kg), this is considered a treatment failure.

Patients **in clinical remission with full donor chimerism** at 6 months post-transplant will **not** receive DLI. If, at future assessment, there is evidence of either disease relapse OR mixed chimerism, patients may commence DLI as per the above dose schedule.

Chimerism, Minimal Residual Disease and donor leukocyte infusions in patients receiving non-myleoblative conditioned alloHSCT

Prior to transplant, peripheral blood or a buccal swab will be collected from **both** the donor and recipient and sent to the site's local chimerism laboratory to allow the selection of informative PCR primers.

Whenever unrelated donors are used, centres are encouraged, wherever possible, to store aliquots of donor leukocytes for subsequent donor leukocyte infusions (DLI).

In order to determine how and when these should be used, quantitative, lineage-specific (myeloid + T-cell) chimerism should be performed by microsatellite PCR on peripheral blood and minimal residual disease (MRD) will be quantified by Ig/TCR on bone marrow following transplant. An anonymised copy of the local chimerism reports from each timepoint MUST be forwarded promptly to UCL CTC.

Chimerism-triggered DLI

In the absence of GVHD, immunosuppression should be tapered and discontinued by 3

months and chimerism should be tested at 3 months post-transplant. The presence of mixed chimerism (≤95% donor cells in T or myeloid lineages) in the absence of GVHD should lead to initiation of escalated DLI (dose escalation schedule above) from 6 months post-transplant. It is the local investigator's responsibility to check chimerism reports and initiate DLI as necessary. DLI should not be given earlier than 6 months post-transplant unless agreed with the UKALL14 Transplant Coordinator, Prof David Marks.

Two to three months following the first DLI, chimerism testing should be repeated and the presence of persistent mixed chimerism in the absence of GVHD will trigger the next dose of donor leukocytes. If there is a greater than 50% reduction in the percentage of recipient cells at 3 months post DLI, then further chimerism testing should be repeated 2-3 weeks later to allow further time for the establishment of full donor chimerism. Any alteration in the DLI schedule must be discussed with the Transplant Coordinator (Prof David Marks). Investigators are encouraged to contact the Trial Management Group as DLI decisions may be affected by patient characteristics or risk factors.

Repeat chimerism testing ± DLI should be performed every 3 months until the establishment of full donor chimerism or the development of GVHD.

MRD-triggered DLI

MRD will be quantified in bone marrow at 3-monthly intervals post-transplant, beginning at 3 months post-transplant - around the time that the immune suppression has been stopped. If MRD at any quantifiable level is detected, any residual immune suppression on-going should be tapered as rapidly as possible followed by, in the absence of GVHD, commencement of the DLI schedule below, with escalating doses being dependent upon the result of repeat MRD testing. If MRD is detected outside the quantifiable range, repeat testing is recommended in 2 months, if acceptable to the patient and clinician.

7.2.10.8 Multilineage Chimerism monitoring:

Peripheral blood samples for determination of the donor or recipient origin of T-cells and myeloid cells should be performed locally, as per correlative science studies section.

All patients should be followed very carefully until they achieve a high level of donor chimerism (>95%). Subsequent falls in donor chimerism should be confirmed and these patients should be followed carefully, for

evidence of relapse especially if peripheral blood counts have fallen or there are symptoms suggestive of leukaemia.

Regular monitoring of chimerism by XY FISH in sex-mismatched allografts locally is also encouraged as supporting evidence.

Patients with persistent mixed chimerism or a fall in chimerism are candidates for immunotherapy at the protocolised doses. Investigators are encouraged to contact the transplant co-ordinator (Professor David Marks) to discuss these patients and immunotherapy decisions.

7.2.10.9 T-cell depletion in the unrelated donor myeloablative HSCT setting an alternative protocol for centres committed to T-cell depletion.

T-cell depletion is not permitted for sibling allografting and T-cell depletion is not recommended during full intensity unrelated donor allografting. For those centres committed to T-cell depletion, in-vivo alemtuzumab (60mg pre-transplant as 2 x 30mg doses) is permissible.

7.2.11 Post-transplant intrathecal therapy

For those patients having non-myeloablative conditioned transplant, it will be necessary to complete further intrathecal therapy post-transplant. This consists of 8 doses of intrathecal methotrexate (12.5mg) given every 3 months for 2 years following non-myeloablative transplant.

7.3 Management after treatment withdrawal

If a patient withdraws consent or stops trial treatment for any reason e.g. toxicity or lack of efficacy then subsequent treatment will be at the discretion of the treating clinician.

In such cases, patients remain within the trial for the purposes of follow-up and data analysis according to the treatment option to which they have been allocated.

7.4 Out-of-hours medical care

Medical care, including out-of-hours medical care is the responsibility of the site. Sites must ensure that all patients registered onto the trial receive a copy of the UKALL14 patient card and that the site on-call contact details have been added.

8.0 Assessments

8.1 Assessment time points

Information is required for patients at the following time points:

	Chemo-only patients	Non-myeloablative transplant patients	Myeloablative transplant patients
Initial diagnosis & pre treatment (see section 5.1)	X	X	X
Post induction phase 1 (at count recovery)	X	X	X
Post induction phase 2 (at count recovery)	X	X	X
After intensification	X	X	X
After each cycle of consolidation	X	If given	If given
Every 3 months during maintenance therapy	X	-	-
Pre-transplant	-	X	X
Day 100 post-transplant	-	X	X
Every 3 months until 2 years post-transplant	-	X	X
Annual follow up	X<br>From anniversary of completion of maintenance or early withdrawal from treatment	X<br>From anniversary of completion of 2 years' follow up post-transplant	X<br>From anniversary of completion of 2 years' follow up post-transplant

PLEASE SEE STUDY VISIT SCHEDULE (APPENDIX 12) FOR FURTHER DETAILS

8.1.1 Routine Clinical and Laboratory Assessments during treatment

a) Clinical examination will be carried out daily or more often, if the patient is acutely unwell, as part of routine clinical care.
b) Assessment of performance status ECOG will be carried out before each discrete block of therapy.
c) Height/Weight & BSA will be assessed as needed to prescribe each block of therapy.
d) Full blood count and other laboratory tests e.g. LFTs, U&Es will be carried out at the clinician's discretion as part of the routine management of acute leukaemia. These tests are usually carried out a minimum of three times weekly during inpatient stays.
e) Remission status (% blasts) to be assessed at the end of each treatment block
f) Number of inpatient days to be assessed at the end of each treatment block
g) Adverse event assessment

8.1.2 Schedule of testing for MRD and correlative science testing

	**Specimens for local assessment**	**Specimens for correlative studies to be sent to central laboratory** (Samples to be sent to UCL Cancer Institute unless otherwise stated. See appx. 8 for lab addresses)*
**At Diagnosis**	Cytogenetics/molecular assessment of BCR-ABL and MLL on bone marrow. A copy of the results should be sent to the Leukaemia Research Cytogenetics Group (LRCG) at Newcastle University. (Please refer to appx. 7 for details of the LRCG). Tissue typing of patient and any siblings to be carried out. Donor search to be initiated if no matched sibling donor available.	Bone marrow 3-5ml in EDTA (OR peripheral blood 30-50ml in EDTA if bone marrow not available)<br>BCR-ABL status will also be checked.<br><br>If patient has consented: buccal swab for constitutional DNA analysis (for patients registered from implementation of protocol v7.0)
**During Phase 1 therapy**	As per local practice	5ml peripheral blood in a serum tube (for Asparaginase activity assay and anti-Asparaginase antibodies) taken on d3 or d4 and on d18 (immediately prior to doses 1 and 2 of pegylated asparaginase for patients aged ≤40), or taken on d18 (prior to pegylated asparaginase administration) and d32 in patients aged ≥41. **Not applicable to Philadelphia positive patients**. On the same day as the pre-asparaginase sample is taken, a 4.5ml sodium citrate tube should be filled (to the line) to collect plasma. Spin the tube @ 2000G for 5 mins, aliquot serum into 5 eppendorfs and store locally at –80°C if possible or if not, -20°C.***
**At recovery** post Phase 1**	Bone marrow aspirate for remission assessment locally. Same day local FBC to determine peripheral count.	Bone marrow aspirate: 3-5ml in EDTA for MRD assessment - <u>**Result not reported to sites**</u> (Appx. 8)<br>(IgH/TCR rearrangements for Ph-ALL, BCR-ABL for Ph+ALL)
**At recovery** post Phase 2**	Bone marrow aspirate for remission assessment locally. Same day local FBC to determine peripheral count.	Bone marrow aspirate: 3-5ml in EDTA for MRD assessment - <u>**Result reported to site within 10 working days of receipt of sample**</u> (Appx. 8)<br>(IgH/TCR rearrangements for Ph-ALL, BCR-ABL for Ph+ALL)
**During intensification:**	As per local practice	5ml peripheral blood in a serum tube (for Asparaginase activity assay and anti-Asparaginase antibodies) taken on d2 and d16 of intensification (immediately prior to doses 3 and 4 of pegylated asparaginase). On the same day as the pre-asparaginase sample is taken, a 4.5 ml sodium citrate tube should be filled (to the line) to collect plasma. Spin the tube @ 2000G for 5 mins, aliquot serum into 5 eppendorfs and store locally at –80°C if possible or if not, -20°C.***
**Pre-transplant** **(non-myeloablative only):**	As per local practice.<br><br>Peripheral blood or buccal swab from <u>donor and recipient to be sent to local chimerism lab</u> for assessment of mini-satellite regions. An anonymised copy of the results must be sent promptly to UCL CTC.	N/A
**Post-transplant** **(non-myeloablative only):**	As per local practice.<br><br>Peripheral blood in EDTA to be sent to local chimerism lab. An anonymised copy of the results must	3-5 ml bone marrow aspirate in EDTA for MRD assessment.<br>First sample to be sent 3 months post-transplant. To be repeated every 3 months until 2 years post-transplant or until relapse. <u>**Result reported to site within 10 working days of receipt of sample**</u> (Appx. 8)

	be sent promptly to UCL CTC.<br><br>First sample to be sent 3 months post-transplant. To be repeated every 3 months until 2 years post-transplant or until relapse.	
**At Relapse**	As per local practice	3-5ml bone marrow aspirate in EDTA (OR peripheral blood 30-50ml in EDTA if WCC > 30x 10^9/l).

***Please refer to Appendix 8 for guidelines for sending specimens to the central laboratory**
** "Recovery" is defined as neutrophils >0.75 x 10^9/l, platelets >75 x 10^9/l.
*** Sites to collect, process and store these samples if possible depending on resources, however this is optional.

8.1.3 Assessments during follow-up

All patients will be followed up annually until the end of the study, or until death.

Follow-up begins when trial treatment is completed, i.e. after the last dose of maintenance therapy has been given. In the case of patients treated by transplantation, precise definition of when treatment becomes follow-up is difficult due to the wide variation in the clinical scenarios. Hence follow-up will be arbitrarily defined to commence 2 years after the date of stem cell infusion, to coincide with the length of maintenance therapy.

a) Clinical examination as necessary (often there are no expected abnormal findings)
b) Full blood count
c) Any other tests as dictated by the patient's clinical condition
d) Assessment of disease status
e) Record of any occurrence of AVN or serious cardiac problems
f) Employment history post treatment of ALL
g) GVHD

At the 2 year follow up appointment (2 years after stopping maintenance/4 years post stem cell infusion), as well as the above annual assessments, patients will also be assessed for late effects of treatment:

a. General Health Questionnaire (GHQ-12) Appendix 13
b. Symptoms of heart failure
c. Echocardiogram
d. Avascular necrosis
e. GVHD
f. Mental health
g. Fertility
h. Any other cancer diagnosis since ALL diagnosis.
i. Employment history post treatment of ALL

If a patient fails to attend clinic for any visit then the site must make every effort to gain follow up information as requested. If a patient no longer attends clinic (e.g. moves away or is discharged from clinic), it is the duty of the site to inform UCL CTC of where follow up information may be obtained (e.g. GP, alternate transplant centre). Patients will also be consented to follow up through the NHS Information Centre.

9.0 Correlative Science

PLEASE ALSO SEE SECTION 8.2.1 and APPENDICES 7, 8 and 12 FOR FURTHER DETAILS INCLUDING CONTACT NAMES AND ADDRESSES.

Four distinct but inter-related scientific correlative studies are planned, closely integrated with the clinical questions being asked. Minimal residual disease (MRD) testing will be carried out as a matter of course, as an integral part of the risk-stratified treatment allocation. The Adult ALL MRD laboratory at UCL Cancer Institute will provide specimen reception, processing and storage facilities and carry out the MRD testing, providing the infrastructure for the duration of the study.

Tests and analyses will be performed in 3 places (a) UCL Cancer Institute, London (b) Northern Institute of Cancer Research (NICR), Newcastle University & CRUK-PIMS, (c) Medac, Germany. Data will ultimately be collated with clinical outcome data held by UCL Cancer Trials Centre.

Other ethically approved analyses may be performed on stored samples subject to patient consent.

9.1 Aim 1. To determine the relationship between CD20 on ALL blasts and response to monoclonal antibody therapy

9.1.1 Background

The ability to combine rituximab (antiCD20) with chemotherapy (resulting in considerable improvement in outcome) in the treatment of lymphoma coupled with the expression of CD20 in ALL of B-cell precursor-cell type has lead to the introduction of rituximab in the treatment of B-cell precursor (BCP) ALL. The expression of CD20 on ALL blasts varies widely (E. Paietta, personal communication) CD20 is less commonly or less highly expressed on B-ALL blasts than some other B-cell antigens. However, there is accumulating evidence that it is of prognostic significance.Upon instituting an investigation into the use of anti-B-cell monoclonal antibodies in ALL, the investigators realised that there was no answer to the recurrent question asked in review 'why do you not limit anti-CD20 antibody administration only to those with CD20 positive tumours'. No data in ALL - or other tumour - were available to define the relationship between antigen expression and response and suggest a threshold level for response. Particularly intriguing in this regard is a recent demonstration that the relatively modest expression level of CD20 at diagnosis was often dramatically up-regulated, both in numbers of cells expressing the antigen and levels of expression per cell, following induction chemotherapy. This was shown in vitro to occur on exposure to glucocorticoids, and correlated well with in-vitro rituximab-induced killing[9]. We think this kind of study offers an ideal opportunity to investigate this important issue further.

9.1.2 Plan of investigation

In order to investigate the hypothesis that response to monoclonal antibody therapy may have no clear relation to the density of relevant antigen expression at diagnosis, we plan to investigate the relationship between density of CD20 antigen expression at diagnosis and response to monoclonal antibody therapy in all

patients with B-lineage ALL who are participating in the induction randomisation. Flow cytometry will be used to quantify CD20 antigen expression (percentage expression and mean fluorescence intensity MFI) at diagnosis. Correlation between percentage CD20 expression and response to initial therapy (CR rate and quantitative MRD level after both phases of induction) will be determined for both randomisation groups, the group receiving no monoclonal antibodies serving as a negative control group.

9.2 Aim 2. To determine whether the administration of an anti-B-cell monoclonal antibody as part of induction therapy for ALL limits the extent of anti-asparaginase antibody formation and promotes asparagine depletion.

9.2.1 Background

L-asparaginase is arguably one of the most valuable drugs in the treatment of ALL. However, it is associated with numerous toxicities. When these occur early in treatment, therapeutic delays are often generated which can result in compromise of overall therapy. The appropriate dose, preparation and formulation of L-asparaginase remain unresolved. In paediatric practice, pegylated L-asparaginase (pegylated asparaginase) is less immunogenic and gives the most appropriate pharmacokinetic and pharmacodynamics but evidence that this agent could be properly used in adults was lacking until a recent CALGB phase II study used pegylated asparaginase as part of a multi-agent regimen. Effective asparaginase depletion was achieved in some adults [15]although increasing age was associated with significantly decreased pegylated asparaginase doses and less asparagine depletion, furthermore, there was still a significant number of patients who developed of anti-asparaginase antibodies; this correlated with a less successful asparagine depletion. Within the associated clinical study UKALL14, pegylated asparaginase will be administered to adults for the first time in a large phase 3 setting.

9.2.2 Plan of Investigation

We hypothesise that the rate of anti-asparaginase antibody formation will be reduced by the co-administration of anti-CD20 and that this can result in higher levels of asparaginase activity. We will test this hypothesis by quantifying anti-asparaginase antibody levels and asparaginase activity prior to pegylated asparaginase infusion and at time-points specified in section 8.2.2 post pegylated asparaginase infusion. The 2-way randomisation provides the control groups (anti-CD20, or neither) necessary to adequately address this question.

9.3 Aim 3. To perform genomic profiling in order to discover and characterise novel prognostic markers and to identify known copy number alterations (CNA).

9.3.1 Background

ALL is a heterogeneous disease at the genetic level and numerous genetic abnormalities have been described which correlate with demographic, clinical and outcome parameters. Karyotype is one of the most important risk factors in adult ALL[46]. Genomic copy number arrays have now revolutionised our understanding of the genetic basis of childhood ALL and have identified novel subgroups which correlate with clinical parameters, genetic subtypes and outcome[55],[56]. Currently, there is very limited genomic

information available for adult ALL. In one study, SNP arrays revealed a series of cryptic genomic abnormalities but it was too small to establish any novel subgroups or correlate the findings with clinical parameters or outcome[57]. UKALL14 provides a valuable opportunity to collect diagnostic and remission samples to identify novel CNAs within a large cohort. We will focus on Philadelphia chromosome (Ph) negative BCP-ALL, since T-ALL is better understood at the genetic level and shares a greater homology with paediatric ALL[58].

9.3.2 Plan of Investigation

We plan to analyse DNA from 400 diagnostic samples using the Affymetrix SNP6.0 array (or later version). We will generate a normal cohort for comparison by analysing DNA from 50 MRD-negative post phase II remission samples. Data from this cohort will be used alongside the publicly available HapMap control dataset to remove copy number variants (CNV), using paired and unpaired normalisation. All labelling and hybridisation procedures will be outsourced to CRUK-PIMS. The raw data files will be returned to the LRCG where it will be processed, stored and analysed using a combination of in-house tools, freeware and commercially available software (e.g. Affymetrix Genotyping Console, dChip, Partek and Nexus). Where necessary, CNAs identified by the SNP arrays will be confirmed using home-grown and commercially available FISH probes. The LRCG has experience analysing large complex datasets and integrating data from different technologies e.g. genomic, gene expression and micro-RNA data [59-61]

Using cytogenetic and FISH data generated during the patients diagnostic work-up we will determine whether each novel CNA co-exists with known chromosomal abnormalities. This will allow us to assess whether these novel lesions are likely to represent primary genetic aberrations which might define new biological subgroups or secondary abnormalities which are likely to be cooperating mutations. Running high resolution SNP arrays will reveal novel CNAs and also identify known CNAs which are beyond the detection limits of standard diagnostic technologies (e.g. Ikaros deletions, which have shown to be associated with a poor outcome in childhood ALL and are frequently <85kb[56]. Running this array-based project in conjunction with a clinical trial will allow the clinical relevance of these novel and existing genetic lesions to be assessed in a reasonable timeframe. We will investigate the relationship of each CNA with the age, sex, white cell count, immunophenotype and other clinical parameters. Most importantly, we will be able assess their prognostic relevance in the context of a modern treatment protocol and be able to consider a variety of clinically relevant endpoints (e.g. CR status, MRD status, BM and CNS relapse and death). Assuming an overall event rate of 50%, the analysis of 400 patients will give us 80% power to detect a 20% difference in outcome for an abnormality found in 20% patients. This magnitude of effect was observed for cytogenetic subgroups in the previous trial[46] and novel CNAs of this frequency have been discovered in childhood ALL using the same platform[55]. Standard statistical tests will be used and all analyses will be conducted in collaboration with the trial statistician as in previous studies.

This project will identify a number of genomic abnormalities whose functional consequence will need to be fully evaluated in order to elucidate their contribution to leukaemogenesis and assess their potential as therapeutic targets. Utilising total RNA extracted from the same diagnostic samples used for the SNP arrays

we plan to further investigate different genetic lesions using gene expression arrays (Affymetrix Exon 1.0 ST array) and micro-RNA arrays (Agilent miRNA microarray Rel12.0). These additional scientific studies will require additional funding; however, the LRCG is an excellent position to attract such funding. We have ongoing collaborations within NICR with experts in functional analysis (Dr Olaf Heidenreich), molecular pharmacology (Dr Julie Irving) and molecular carcinogenesis (Dr James Allan). In addition, we are well positioned to take forward any identified therapeutic targets through the CRUK Drug Discovery programme in collaboration with Herbie Newell, Professor of Cancer Therapeutics, NICR and Roger Griffin, Professor of Medicinal Chemistry.

9.4 Aim 4. To determine whether the speed at which full donor chimerism is achieved in the T –cell compartment correlates with the level of molecularly determined minimal residual disease.

9.4.1 Background

Since the age threshold at which TRM exceeds reduction in relapse risk may be as low as 35 to 40 years old[27], it is very reasonable to examine non-myeloablative HSCT (previously reported only in retrospective studies) as a way to provide a graft versus leukaemia effect with reduced toxicity in adult ALL and this will be investigated in UKALL14. The success of this approach is likely to be disease burden dependent - absence of MRD at the time of transplant and the speed at which full donor chimerism[42] can be achieved may be of crucial importance. This has never previously been studied. Hence, the main scientific question to be addressed in this correlative study is whether the graft versus leukaemia effect is of sufficient magnitude and appropriate in timescale to deliver an effective anti-leukaemia therapy.

9.4.2 Plan of investigation

In order to investigate this question thoroughly, mixed-lineage chimerism analysis should be performed on peripheral blood - fractionated into myeloid, T-cell and B-cell populations using immunomagnetic beads - at 3, 6, 9, 12, 15, 18, 21 and 24 months post-transplant. The degree of donor/recipient chimerism will be assessed with PCR analysis of informative mini-satellite regions. Peripheral blood or a buccal swab must be collected from both the donor and patient prior to the transplant for assessment of mini-satellite regions. Chimerism testing will be carried out at local chimerism laboratories and a report from each timepoint sent promptly to UCL CTC.

For patients receiving DLI as defined in the protocol, the chimerism status should be assessed 2 months after each DLI infusion, until full donor chimerism is obtained.

Minimal residual disease will be assessed at the same time-points, using immunoglobulin gene or T-cell receptor gene re-arrangements as identified at diagnosis.

9.5 Aim 5. Assessment of late effects

9.5.1 Aims

To formally assess the late effects of ALL therapy for all patients on the trial, whether they have received chemotherapy alone or an allograft.

To identify and describe some of the adverse physical and psychosocial consequences of the disease and its treatment.

9.5.2 Background & Planned Investigations

A great deal of research has been carried out on the late effects caused by chemotherapy for acute lymphoblastic leukaemia in children. It is well documented that approximately 2 out of every 3 survivors from childhood cancers, including ALL, will suffer at least one late effect. One in four survivors will experience a late effect that is life threatening[1,2].

There are data on late effects of myeloablative transplantation in adults with ALL, but less research has been done in adult ALL sufferers treated with chemotherapy alone and there are minimal data on the effects of non-myeloablative allografting for ALL to date. We presume that the late effect rate in adults will be at least equivalent to that seen in children, if not worse, but we do not know that this is the case. Part of our role as clinicians is to inform patient choice on the best therapeutic choice available to them. Survival rates are obviously a key part of any such discussions, but increasingly we need to look at the quality of survival offered by available therapies when helping patients consider their options. The aim of this part of the trial is to collect some basic information regarding late effects of diagnosis and treatment of ALL in adults that may inform such discussions with our patients in the future.

Late effects that are known to occur as a consequence of chemotherapy include neurocognitive problems, premature menopause, cardiorespiratory dysfunction, sexual impairment, infertility, chronic fatigue and pain syndromes, and second malignancies. Research shows that many survivors also experience significant negative psychosocial outcomes, including fear of recurrence, poor self-esteem, anxiety and depression, employment and insurance discrimination, and relationship difficulties[5].

Historically, survivorship research has sought to identify and address the adverse physical and psychosocial consequences of the disease. An emerging body of evidence suggests that cancer survivors, similar to survivors of other traumatic life events, also experience positive life changes following a cancer diagnosis, and that these positive effects of cancer frequently coexist with the negative[6, 7]. Studies in adult cancer survivors have shown that many survivors identify their illness experience as an event that has allowed them to make positive lifestyle changes resulting in higher quality of life scores than the general population[8, 9]. We therefore want to use the general health questionnaire-12 (GHQ-12) as part of our screening for treatment related late effects. This is a reliable screening instrument for psychological distress in all clinical groups and can be used for assessing both positive and negative aspects of mental health.

Whilst some late effects may occur after many years e.g. second malignancies, other effects such as infertility can be assessed at much earlier time points. The aim of this part of the study is to initially assess each patient for late effects 2 years following the end of therapy. This will be 4 years from diagnosis for those having received chemotherapy only whereas it will be less than 3 years from diagnosis in some cases where allografting has been used. This part of the study is in addition to the routine data collected for all transplant patients.

9.6 Schedule of testing for correlative science studies

Please see section 8.1.2 - Schedule of testing for MRD and correlative science (please also see appendix 8).

9.7 Constitutional DNA Samples

DNA from non-leukaemic samples will be analysed at the UCL Cancer Institute for genetic changes that may be associated and/or linked with ALL in adults. This DNA will be obtained in one of two ways depending on when the patient was registered and their MRD status:

- For patients registered on the study prior to protocol v7.0, constitutional DNA can be obtained from bone marrow remission samples (provided a MRD negative status was achieved), which have been collected for MRD studies and then stored at UCL Cancer Institute. If these patients did not achieve a MRD negative status then a buccal swab will be taken to collect DNA.
- For patients who consent for constitutional DNA studies and are registered into the study from the implementation of protocol v7.0 onwards, a buccal swab will be taken around the time of study registration.

<u>Consent</u>

Information about the samples required for translational research/constitutional DNA analysis will be presented in one of two additional patient information sheets; one for consent to use previously collected bone marrow samples and one for the collection of a buccal swab.

- The patient information sheet for consent to extract constitutional DNA from stored bone marrow samples should only be used for patients entered prior to the implementation of protocol v7.0 for whom there is a stored remission sample at the MRD laboratory. UCL CTC will provide sites with a list of patients for whom this patient information sheet should be used.
- For the remainder of patients, the buccal swab patient information sheet should be used.

<u>Samples required</u>

<u>***Bone marrow*** – This sample will have already been collected and stored, no additional sample is required.</u>

<u>***Buccal swab*** – Cheek cells to be swabbed at baseline (or later in pre-protocol v7.0 patients who have never achieved CR) as follows:</u>

1. Verify that the patient's mouth is empty
2. Wash or sanitise hands then put on gloves and mask
3. Carefully remove swab from package
4. Avoid touching swab tip with gloves or against any surface
5. Have the patient open his or her mouth and immediately bring swab tip to inside of cheek
6. Gently rub and rotate swab along the inside of the cheek for 5-10 seconds, ensuring that the entire swab-tip has made contact with the cheek
7. Immediately remove swab, being careful not to touch swab tip against teeth, lips, or other surface

8. Place swab directly into dry transport tube or collection envelope
9. Label the tube or envelope with the patient's trial number and initials
10. Complete the Buccal Swab Sample Request Form and include with the swab
11. Send to the following address by first class post:

Minimal Residual Disease Laboratory
URGENT UKALL14 STUDY SAMPLE (***FAO Adele Fielding or Krisztina Alapi***)
UCL Cancer Institute
Paul O'Gorman Building
72 Huntley Street
London WC1E 6DD

MRD Lab email: ALLMRDlab@ucl.ac.uk

Sample processing & analysis

The laboratory will extract DNA and appropriate genomic analysis will be applied. Results will not be fed back to sites or patients.

10.0 Data Collection & Management

All documents must be available for inspection by the appropriate authorities upon request.

Data will be collected from sites on version controlled case report forms (CRFs) designed for the trial and supplied by UCL CTC. Data must be accurately transcribed onto CRFs and must be verifiable from source data at site. Examples of source documents are hospital records which include laboratory and other clinical reports etc.

Where copies of supporting source documentation (e.g. autopsy reports, pathology reports, CT scan images etc.) are being submitted to UCL CTC, the patient's trial number must be clearly indicated on all material and any patient identifiers removed/blacked out prior to sending to maintain confidentiality.

10.1 Completing Forms

The original CRFs do not need to be sent to the UCL CTC however a copy must be kept at site. All entries must be clear and legible. The use of abbreviations and acronyms must be avoided. The treating clinician is responsible for the accuracy of all data reported in the CRF. All CRFs must be signed off by staff who are listed on the site staff delegation log as performing this duty.

10.2 Corrections

Any corrections must be made by drawing a single line through the incorrect item whilst ensuring that the previous entry is not obscured. Each correction must be dated and initialled. Tippex must not be used.

10.3 Missing Data

To avoid the need for unnecessary data queries CRFs must be checked at site for blank fields before sending to the UCL CTC. When data is unavailable because a measure has not been taken or test not performed, enter "ND" for not done. If a measure was not required at the particular time the form relates to, enter "N/A" for not applicable. When data is unknown enter the value "NK" (only use if every effort has been made).

10.4 Queries

Data arriving at UCL CTC will be checked for legibility, completeness, accuracy and consistency, including checks for missing or unusual values. Query Reports will be sent to the data contact at site. When responding to a query, site staff must print the query report, annotate their response on the report and ensure each page has been signed and dated by a member of staff delegated to data management duties on the Delegation Log and return the original copy to UCL CTC. There is no need to send updated CRFs unless specifically requested.

10.5 Submission Timelines

UK sites must complete and return CRFs to the UCL CTC within one month of the patient being seen.
Sites outside the UK must complete and submit all CRFs to UCL CTC within one month of the patient being assessed.

10.6 Archiving of Trial Documentation

At the end of the trial, the UCL CTC will archive securely all trial related documentation for 5 years. Arrangements for confidential destruction will then be made. It is the responsibility of PIs to keep all essential documents relating to the trial for a minimum of 5 years after the end of the trial and in accordance with national legislation and for the maximum period of time permitted by the site. Essential documents are those which enable both the conduct of the trial and the quality of the data produced to be evaluated and show whether the site complied with the principles of Good Clinical Practice and all applicable regulatory requirements.

11.0 Pharmacy

Please see separate UKALL14 drug supply document for initial drug supply guidelines, section 7.0 for treatment schedule and appendices 2-4 for full details of IMPs and supporting medications.

11.1 Pharmacy responsibilities

All pharmacy aspects of the trial at participating sites are the responsibility of the PI, who may delegate this responsibility to the local pharmacist or other appropriately qualified personnel, who will be the Pharmacy Lead. The delegation of duties must be recorded on the site staff delegation log.

Rituximab (Mabthera®), pegylated asparaginase (Oncaspar®), nelarabine (Atriance®) and palifermin (Kepivance®) supplied for the UKALL14 trial are for randomised UKALL14 patients only and must not be used outside the context of this protocol.

Please see separate UKALL14 drug supply guidelines and Clinical Trial Site Agreement.

11.2 Drug accountability

Accountability for Rituximab, Nelarabine and Pegylated Asparaginase at participating sites is the responsibility of the Principal Investigator, who may delegate this responsibility to the local pharmacist, or other appropriately qualified personnel. The responsible person will ensure that the Rituximab, Nelarabine and Pegylated Asparaginase are used only in accordance with this protocol and that appropriate drug accountability records are maintained.

The trial drugs must not be used outside the context of this protocol. Under no circumstances should the site investigator or other site personnel supply trial drug to other investigators, patients, or clinics, or allow supplies to be used other than directed by this protocol without prior authorisation from the Supplier and notification to the Sponsor.

The site pharmacy must maintain drug accountability records for the three drugs listed above including receipt dispensing, returned medication, storage conditions and destruction of returned/unused medication. Sites are permitted to use their own drug accountability systems as long as the required information above is recorded and available to the Sponsor. Copies of complete drug accountability logs must be submitted to UCL CTC for all trial patients upon request. Also refer to section 16.1.1 (Central Monitoring).

Accountability is not required for non-IMPs.
The site pharmacy should follow local procedures for traceability of non-IMPs.

11.3 Temperature Excursions

All temperature excursions outside the storage conditions specified in the SPC(s) and Drug Supply Guidelines must be reported to UCL CTC as per the 'Pharmacy Procedure for Reporting Temperature Excursions' (see Pharmacy Site File).

Upon identifying an excursion:

- all affected trial stock must be quarantined IMMEDIATELY
- the 'Notification of Temperature Excursion' form must be completed and e-mailed to ctc.excursions@ucl.ac.uk or faxed to 020 7679 9861.

Please note that UCL CTC must be informed immediately if a patient has been administered drug affected by a temperature excursion.

12.0 Pharmacovigilance

12.1 Definitions of Adverse Events

The following definitions have been adapted from Directive 2001/20/EC, ICH E2A "Clinical Safety Data Management: Definitions and Standards for Expedited Reporting" and ICH GCP E6:

Adverse Event (AE)

Any untoward medical occurrence or effect in a patient treated on a trial protocol, which does not necessarily have a causal relationship with a trial treatment. An AE can therefore be any unfavourable and unintended sign (including an abnormal laboratory finding), symptom or disease temporally associated with the use of a trial treatment, whether or not related to that trial treatment.

Adverse Reaction (AR)

All untoward and unintended responses to a trial treatment related to any dose administered. A causal relationship between a trial treatment and an adverse event is at least a reasonable possibility, i.e. the relationship cannot be ruled out.

Serious Adverse Event (SAE) or Serious Adverse Reaction (SAR)

An adverse event or adverse reaction that at any dose:

- Results in death
- Is life threatening (The term "life-threatening" refers to an event in which the subject was at risk of death at the time of the event. It does not refer to an event that hypothetically might have caused death if it were more severe.)
- Requires in-patient hospitalisation or prolongs existing hospitalisation
- Results in persistent or significant or disability/incapacity
- Is a congenital anomaly or birth defect
- Is otherwise medically significant (i.e. important medical events that may not be immediately life-threatening or result in death or hospitalisation but may jeopardise the patient or may require intervention to prevent one of the other outcomes listed above).

Suspected Unexpected Serious Adverse Reaction (SUSAR)

A serious adverse reaction, the nature or severity of which **is not consistent** with the applicable trial treatment information.

12.2 Reporting Procedures

12.2.1 All Adverse Events (AEs)

All adverse events must be recorded in the patient notes. The maximum severity grade of all adverse events that occur between informed consent and 30 days post consolidation (for non transplant patients) or 30 days post-transplant (for transplant patients) must be recorded on the trial CRFs. Those meeting the

definition of a Serious Adverse Event (SAE) must also be reported to the UCL CTC using the trial specific SAE Report. Also refer to section 12.2.2 (Serious Adverse Events (SAEs)).

Pre-existing conditions do not qualify as adverse events unless they worsen.

12.2.1.1 Overdoses

All accidental or intentional overdoses, whether or not they result in adverse events, must be recorded in the patient notes and CRFs. Overdoses resulting in an adverse event are classified as SAEs and must also be reported to UCL CTC according to SAE reporting procedures. The fact that an overdose has occurred must be clearly stated on the SAE Report. Also refer to section 12.2.2 (**Serious Adverse Events (SAEs)).**

Sites must inform UCL CTC immediately when an overdose has been identified. Also refer to section 13 (Incident Reporting and Serious Breaches).

12.2.1.2 Adverse Event Term

An adverse event term must be provided for each adverse event, preferably using the term listed in the Common Terminology Criteria for Adverse Events (CTCAE) v4.0 available online at: http://evs.nci.nih.gov/ftp1/CTCAE

12.2.1.3 Severity

Severity for each adverse event must be determined by using the Common Terminology Criteria for Adverse Events (CTCAE) v4.0 as a guideline, wherever possible. The criteria are available online at: http://evs.nci.nih.gov/ftp1/CTCAE

In those cases where the CTCAE criteria do not apply, severity should be coded according to the following criteria:

1 = Mild (awareness of sign or symptom, but easily tolerated)
2 = Moderate (discomfort enough to cause interference with normal daily activities)
3 = Severe (inability to perform normal daily activities)
4 = Life threatening (immediate risk of death from the reaction as it occurred)
5 = Fatal (the event resulted in death)

12.2.1.4 Causality

The PI, or other delegated site investigator must perform an evaluation of causality for each adverse event. Causal relationship to the trial treatment must be determined as follows:

- **None**

There is no evidence of any causal relationship.

- **Unlikely**

There is little evidence to suggest a causal relationship (e.g. because the event did not occur within a reasonable time after administration of the trial treatment). There is another reasonable explanation of the event (e.g. the patient's clinical condition, other concomitant treatments).

- **Possibly**

There is some evidence to suggest a causal relationship (e.g. because the event occurs within a reasonable time after administration of the trial treatment). However, the influence of other factors may have contributed to the event (e.g. the patient's clinical condition, other concomitant treatments).

- **Probably**

There is evidence to suggest a causal relationship and the influence of other factors is unlikely.

- **Definitely**

There is clear evidence to suggest a causal relationship and other possible contributing factors can be ruled out.

UCL CTC will consider events evaluated as possibly, probably or definitely related to be adverse reactions. On adverse event case report forms, a causality assessment is required only for treatment administered within the 30 days prior to onset.

On SAE reports, the PI or co-investigator must assign a causality assessment for each of the IMPs to which the patient has been exposed, regardless of the time has elapsed since administration, in order to demonstrate consideration of whether the SAE is a late effect of a previously administered IMP.

12.2.2 Serious Adverse Events (SAEs)

The table in section 12.2.2.1 below summarises the SAE reporting windows for the UKALL14 trial. All SAEs occurring during these timepoints (or after these dates if the site investigator feels the event is related to one of the IMPs) must be submitted to UCL CTC by fax within **24 hours** of observing or learning of the event, using the trial specific SAE Report. All sections on the SAE Report must be completed. If the event is <u>not reported within 24 hours</u> to UCL CTC, the circumstances that led to this in the SAE report must be detailed to avoid unnecessary queries.

The PI or co-investigator must assign a causality assessment for each of the IMPs to which the patient has been exposed, regardless of the time has elapsed since administration.

12.2.2.1 SAE reporting timeframes

SAE Reporting Timeframes				
		**Randomisation**	**Timeframe (When IMP given)**	**Timeframe (When no IMP given)**
Phase 1 induction		B1	From informed consent until 30 days post last Pegylated Asparaginase administration	48 days from the date of randomisation for patients who do not receive Pegylated Asparaginase
		B2	From informed consent until 30 post last Rituximab administration	54 days from the date of randomisation for patients who do not receive Rituximab.
		T1, T2	From informed consent until 30 days post last Pegylated Asparaginase administration	48 days from the date of randomisation for patients who do not receive Pegylated Asparaginase
Phase 2 induction		B1, B2	Not required unless within phase 1 induction SAE reporting window or late effect of IMP	Not required unless within phase 1 induction SAE reporting window
		T2 after Nelarabine administration	From first dose of Nelarabine until 30 days post last Nelarabine administration	From count recovery at the end of Phase 2 Induction until 35 days after count recovery at the end of Phase 2 Induction for patients not who do not receive Nelarabine
Intensification		B1, B2, T1, T2	From first administration of Pegylated Asparaginase until 30 days post last Pegylated Asparaginase administration	Not required unless within phase 2 SAE reporting window
Consolidation	1	B1, B2, T1, T2	From first administration of Pegylated Asparaginase until 30 days post last Pegylated Asparaginase administration	Not required
	2	B1, B2, T1, T2	Not required unless within consolidation 1 SAE reporting window or late effect of IMP	Not required
	3	B1, B2, T1, T2	From first administration of Pegylated Asparaginase until 30 days post last Pegylated Asparaginase administration	Not required
	4	B1, B2, T1, T2	Not required unless within consolidation 3 SAE reporting window or late effect of IMP	Not required
Non-myeloablative transplant		B1, B2, T1, T2	From start of conditioning chemotherapy until 30 days post-transplant	From start of conditioning chemotherapy until 30 days post-transplant
Myeloablative transplant		B1, B2, T1, T2	From start of conditioning until 30 days post-transplant	From start of conditioning until 30 days post-transplant
Maintenance		B1, B2, T1, T2	Not required unless late effect of IMP	Not required

12.2.2.2 Events which do not Require Immediate Reporting on an SAE Report

The following events do not require immediate reporting on an SAE Report for this trial, but must be recorded in the relevant section(s) of the CRF (please see section 12.2.1)

- Disease progression
- Disease related deaths
- GvHD
- Graft Failure
- Secondary Malignancy.

Please note that hospitalisation for elective treatment or palliative care does not qualify as an SAE.

Completed SAE Reports must be faxed
within 24 hours of becoming aware of the event, to UCL CTC
Fax: +44 (0)20 7679 9861

12.2.2.3 Adverse Event Reporting Flowchart

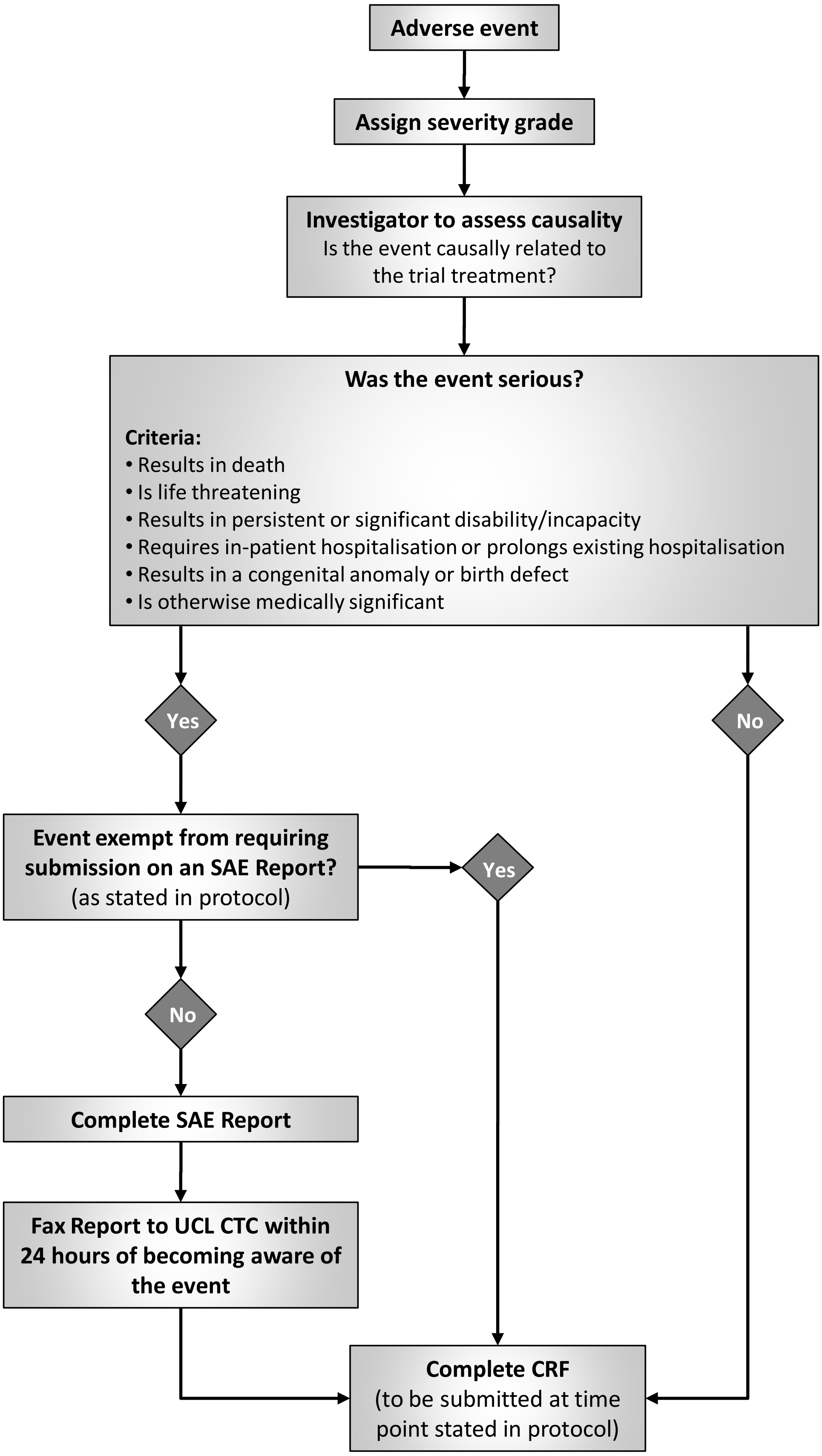

12.2.2.4 SAE Follow-up Reports

All SAEs must be followed-up until resolution and until there are no further queries. The PI, or other delegated site investigator must provide follow-up SAE Reports if the SAE had not resolved at the time the initial report was submitted.

12.2.2.5 SAE Processing at UCL CTC

On receipt of the SAE Report, UCL CTC will evaluate the event for seriousness and expectedness to determine whether or not the case qualifies for expedited reporting.

Expectedness will be evaluated using the list of expected adverse events in the approved SPCs for Rituximab, Pegylated Asparaginase and Nelarabine.

If a patient was treated on arms P1 or P2 prior to closure of palifermin randomisation and has an SAE assessed as causally related to Palifermin, the latest approved SPC prior to the surrender of the EU marketing authorisation will be used.

The CI, or their delegate (e.g. a clinical member of the TMG), may be contacted to review the SAE and to perform an evaluation of causality on behalf of UCL CTC. If UCL CTC has considered expectedness difficult to determine, the CI, or their delegate, will be consulted for their opinion at this time.

Where there are conflicting evaluations of causal relationship by the site and UCL CTC/CI, both opinions will be included in the report.

UCL CTC will submit individual case SAE Reports concerning patients who have been treated with Nelarabine to Novartis within 1 business day of receipt of an SAE report with the minimum data fields completed (correct patient trial number, IMP treatment information, valid CTCAE v4 event term, local investigator causality assessment).

Individual case SAE Reports concerning patients who have been treated with Palifermin will be submitted to Swedish Orphan Biovitrum within 1 business day of receipt of an SAE report with the minimum data fields completed.

Individual case SAE Reports for all SARs for Pegylated Asparaginase will be submitted to Baxalta within 1 business day of receipt of an SAE report with the minimum data fields completed.

Individual case SAE Reports for all SARs for Rituximab will be submitted to Roche within 15 days of receipt of an SAE report with the minimum data fields completed. In addition, UCL CTC will also provide Roche with monthly line listings of SAEs in patients exposed to Rituximab.

12.3 Adverse event of special interest

Venous thromboembolisms (VTEs) are regarded as an adverse event of special interest for this study. Any VTEs occurring during the standard SAE reporting windows (see section 12.2.2.1) should be reported on the study-specific SAE report form within 24 hours of becoming aware of the event.

VTEs occurring outside the SAE reporting windows must be reported on the study-specific Thromboembolic Event Urgent Event Form within 7 calendar days of becoming aware of the event.

12.4 SUSARs

If the event is evaluated as a Suspected Unexpected Serious Adverse Reaction (SUSAR), UCL CTC will submit a report to **the applicable regulatory authority within the EEA** within the following timelines:

- 7 calendar days after the first knowledge of the reaction being fatal/life threatening. If the initial report to the Competent Authority is incomplete, i.e. the UCL CTC has not provided all the information/assessment within 7 days, UCL CTC should submit a completed report based on the initial information within an additional 8 calendar days (the date of the report should not be changed)
- 15 calendar days for significant follow-up information received by UCL CTC on the fatal/life threatening event
- 15 calendar days for initial and follow-up reports of non-fatal/non-life threatening events.

Where possible, the evaluations of causal relationship by both the site and UCL CTC/CI will be reportedUCL CTC will also report all SUSARs the UK REC within the same timelines described above.

12.5 Safety Monitoring

UCL CTC will provide safety information to the TMG and the IDMC on a periodic basis for review.
Trial safety data will be monitored to identify:

- new adverse reactions to the trial treatment regimen or individual trial treatments;
- a higher incidence in rare adverse events than is stated in the IB/SPC for a trial treatment;
- trial related events that are not considered related to the trial treatment regimen.

Should UCL CTC identify or suspect any issues concerning patient safety at any point throughout the trial, the CI or TMG will be consulted for their opinion.

12.6 Pregnancy

If a female patient or a female partner of a male patient becomes pregnant at any point between the start of trial treatment and 12 months post completion of trial treatment, a completed trial specific Pregnancy Report must be submitted to UCL CTC by fax within **24 hours** of learning of its occurrence. The site must request Pregnancy Monitoring Consent from the pregnant patient/partner to collect information on the pregnancy. The trial-specific pregnancy monitoring information sheets and informed consent forms for trial patients and the partners of trial patients must be used for this purpose. If consent is not given by the patient / partner, the notification that a pregnancy has occurred will be retained by UCL CTC, however no further action will be taken on the information detailed in the report.

All pregnancies must be reported by faxing a completed Pregnancy Report within 24 hours of becoming aware of the pregnancy to UCL CTC
Fax: +44 (0)20 7679 9861

12.6.1 Pregnancy Follow-Up Reports

All pregnancies must be followed-up until an outcome is determined. Where the mother has consented for pregnancy follow-up, a follow-up Pregnancy Report must be submitted to UCL CTC by fax within **24 hours** of learning of the outcome. Reports must include an evaluation of the possible relationship of the trial treatment to the pregnancy outcome. If the outcome of the pregnancy meets the criteria for an SAE, an SAE report will also be required.

12.6.2 SAEs During Pregnancy

Any SAE occurring in a pregnant patient must be reported using the trial specific SAE Report, according to SAE reporting procedures. **Refer to section 12.2.2 (Serious Adverse Events (SAEs)) for details.**

12.6.3 Pregnancy Report Processing at UCL CTC

UCL CTC will submit all Pregnancy Reports to Baxalta, Roche, Novartis and Swedish Orphan Biovitrum concerning patients who have been administered the products they supply for the trial in line with the requirements of the trial drug supply agreements for each company.

Should the pregnancy outcome meet the definition of a SUSAR, UCL CTC will submit the report to the MHRA, REC and investigators. **Also refer to section 12.4 (SUSARs).**

12.7 Development Safety Update Reports (DSURs)

Safety data obtained from the trial (for randomised patients only) will be included in DSURs that UCL CTC **will submit to the MHRA and the UK REC**.

UCL CTC will provide Baxalta, Roche, Novartis and Swedish Orphan Biovitrum with DSURs that include information regarding the products they supply for the trial.

13.0 Incident Reporting and Serious Breaches

13.1 Incident Reporting

Organisations must notify UCL CTC of all deviations from the protocol or GCP immediately. UCL CTC may require a report on the incident(s) and a form will be provided if the organisation does not have an appropriate document (e.g. Trust Incident Form for UK sites).

If site staff are unsure whether a certain occurrence constitutes a deviation from the protocol or GCP, the UCL CTC trial team can be contacted immediately to discuss.

UCL CTC will assess all incidents to see if they meet the definition of a serious breach.

13.2 Serious Breaches

Systematic or persistent non-compliance by a site with GCP and/or the protocol, including failure to report SAEs occurring on trial within the specified timeframe, may be deemed a serious breach.

In cases where a potential or actual serious breach has been identified, UCL CTC will inform the MHRA within 7 calendar days of becoming aware of the breach.

UK sites must have written procedures for notifying the sponsor of serious breaches (MHRA Guidance on the Notification of Serious Breaches).

UCL CTC will use an organisation's history of non-compliance to make decisions on future collaborations.

14.0 Withdrawal of patients

In consenting to the trial, patients are consenting to trial treatment, trial follow-up and data collection.

14.1 Withdrawal from Trial Treatment

The site investigator may withdraw a patient from the trial treatment whenever continued participation is no longer in the patient's best interests, but the reasons for doing so must be recorded. Reasons for discontinuing treatment may include:

- Disease progression whilst on therapy
- Unacceptable toxicity
- Intercurrent illness which prevents further trial treatment
- The patient withdraws consent to further trial treatment
- Any alterations in the patient's condition which justifies the discontinuation of treatment in the site investigator's opinion.

In these cases patients remain within the trial for the purposes of follow-up and data analysis according to the treatment option to which they have been allocated. If a patient wishes to withdraw from trial treatment, sites should explain the importance of remaining on trial follow-up, or failing this of allowing routine follow-up data to be used for trial purposes.

14.2 Withdrawal of Consent to Data Collection

If a patient explicitly states they do not wish to contribute further data to the trial their decision must be respected and recorded on the relevant CRF. In this event details should be recorded in the patient's hospital records. Data up to and including the date of withdrawal must be sent to UCL CTC. Thereafter, no further CRFs must be completed and no further data sent to UCL CTC.

14.3 Moving

For patients moving from the area, every effort should be made for the patient to be followed up at another participating trial site and for this new site to take over the responsibility for the patient, or for follow-up via GP. The process for transferring care to another partipating site is as follows:

- Liaise with the proposed new site and obtain written confirmation that they are willing to take over follow-up duties for the patient.
- Complete a Centre Transfer Form (provided as part of the CRF) and send to UCL CTC.
- CRFs relating to visits up to the point of transfer must be completed and submitted to UCL CTC.
- Provide the new site with a copy of the the patient's complete CRF up until the point of transfer.

The original site remains responsible for submitting all data due up to the date of transfer, and resolving any data queries relating to those data. The site to whom they are transferred will be responsible for completing CRFs pertaining to visits after the date of transfer only.

Details of participating study sites can be obtained from the UCL CTC study team upon request.

If the patient cannot be transferred to another participating site, the site that registered them into the trial remains responsible for obtaining and submitting follow up data on the patient.

14.4 Lost to follow-up

If a patient is lost to follow-up every effort should be made to contact the patient's GP (if consented) to obtain information on the patient's status.

Patients lost to follow up can be tracked via the NHS Information Centre.

15.0 Discontinuation/End of the Trial

15.1 Trial closure

For regulatory purposes the end of the trial will be defined as the end of the follow up phase (when the last randomised patient reaches 2 years after the end of maintenance or 4 years after the date of stem cell infusion if a transplant patient) at which point the 'declaration of end of trial' form will be submitted to participating regulatory authorities and ethical committees, as required.

Once the end of trial has been declared, no more prospective patient data will be collected but sites must cooperate with any data queries regarding existing data to allow for analysis and publication of results.

15.2 Archiving of Trial Documentation

At the end of the trial, the UCL CTC will archive securely all centrally held trial related documentation for 5 years. Arrangements for confidential destruction will then be made. It is the responsibility of the PIs to keep all data and essential documents relating to the trial held at site, for a minimum of 5 years after the end of the trial and in accordance with national legislation and for the maximum period of time permitted by the site.

Essential documents are those which enable both the conduct of the trial and the quality of the data produced to be evaluated and show whether the site complied with the principles of GCP and all applicable regulatory requirements.

UCL CTC will notify sites when trial documentation held at sites may be archived. All archived documents must continue to be available for inspection by appropriate authorities upon request.

15.3 Early discontinuation of trial

The trial may be stopped before completion upon recommendation of the TSC or IDMC (see section 16.2). Sites will be informed by UCL CTC in writing of reasons for early closure and actions to be taken with regards to trial patients. Patients should continue to be followed up as per protocol.

15.4 Withdrawal from trial participation by sites

If a site wishes to close to recruitment, the PI must inform UCL CTC in writing. Follow up as per protocol must continue for all patients recruited into the trial at that site and other responsibilities continue as per CTSA.

16.0 Trial Monitoring and oversight

16.1 Monitoring

UK participating sites and PIs must agree to allow trial-related on-site monitoring, Sponsor audits and regulatory inspections by providing direct access to source data/documents as required. Patients are informed of this in the patient information sheet and are asked to consent to their medical notes being reviewed by appropriate individuals on the consent form.

UCL CTC will determine the appropriate level and nature of monitoring required for the trial. Risk will be assessed on an ongoing basis and adjustments made accordingly.

16.1.1 Central monitoring

Sites will be requested to submit screening logs and staff delegation logs to UCL CTC at the frequency detailed in the trial monitoring plan or on request, and these will be checked for consistency and completeness.

Ensuring patient eligibility is the responsibility of the PI or other delegated Investigator(s). Checks of the criteria listed on the registration form will be undertaken by an appropriately trained UCL CTC staff member prior to registration/randomisation. Also refer to section 5.4 (Registration & Randomisations (B & T) at study entry).

Copies of completed drug accountability logs must be returned to UCL CTC for all randomised patients. Sites will be required to submit logs at the frequency detailed in the trial monitoring plan or on request. A proportion of these will be monitored centrally to ensure completeness and correlation with data captured in the CRF. Also refer to section 11.2 (Drug Accountability).

Sites will be requested to conduct quality control checks of documentation held within the Investigator Site File and Pharmacy Site File at the frequency detailed in the trial monitoring plan. Checklists detailing the current version/date of version controlled documents will be provided for this purpose.

Data received at UCL CTC will be subject to review in accordance with section 10.4 (Queries).
Where central monitoring of data and/or documentation submitted by sites indicates that a patient may have been placed at risk (e.g. evidence of an overdose having been administered, indication that dose modifications for an IMP were not observed following an adverse reaction, etc.), the matter will be raised urgently with site staff and escalated as appropriate (refer to section 13 (Incident Reporting and Serious Breaches) and 16.1.2 ('For cause' on-site monitoring) for further details).

16.1.2 'For Cause' On-Site Monitoring

On-site monitoring visits may be scheduled where there is evidence or suspicion of non-compliance at a site with important aspect(s) of the trial protocol/GCP requirements. Sites will be sent a letter in advance

outlining the reason(s) for the visit. The letter will include a list of the documents that are to be reviewed, interviews that will be conducted, planned inspections of the facilities, who will be performing the visit and when the visit is likely to occur.

16.1.3 Monitoring follow up

Following any monitoring visit, the monitor/TC will provide a follow up email to the site, which will summarise the documents reviewed, and a statement of findings, incidents, deficiencies, conclusions, actions taken and/or actions required. The PI at the site will be responsible for ensuring that monitoring findings are addressed in a timely manner, and by the deadline specified.

UCL CTC will assess whether it is appropriate for the site to continue participation in the trial and whether the incident(s) constitute a serious breach. Refer to section 13 (Incident Reporting and Serious Breaches) for details.

16.2 Oversight Committees

16.2.1 Trial Management Group (TMG)

The TMG will include the Chief Investigator, clinicians and experts from relevant specialities and UKALL14 trial staff from UCL CTC. The TMG will be responsible for overseeing the trial. The group will meet regularly and will send updates to PIs (via newsletters or at Investigator meetings) and report to the appropriate NCRI Clinical Studies Group.

The TMG will review substantial amendments to the protocol prior to submission to the REC, MHRA and other applicable regulatory authorities. All PIs will be kept informed of substantial amendments through their nominated responsible individuals.

16.2.2 Trial Steering Committee (TSC)

The role of the TSC is to provide overall supervision of the trial. The TSC will review the recommendations of the Independent Data Monitoring Committee and, on consideration of this information, recommend any appropriate amendments/actions for the trial as necessary. The TSC acts on behalf of the funder(s) and Sponsor.

16.2.3 Independent Data Monitoring Committee (IDMC)

The role of the IDMC is to provide independent advice on the data and safety aspects of the trial. Meetings of the Committee will be held periodically to review interim analyses, or as necessary to address any issues. The IDMC is advisory to the TSC and can recommend premature closure of the trial to the TSC.

16.2.4 Role of UCL CTC

UCL CTC will be responsible for the day to day coordination and management of the trial and will act as custodian of the data generated on the trial (on behalf of UCL). UCL CTC is responsible for all duties relating to pharmacovigilance which are conducted in accordance with section 12 (Pharmacovigilance).

17.0 Statistics

17.1 Population for analysis

The population for this trial will be adult patients age 19-65 years, inclusive. The primary objective of this study is to compare the event-free survival (EFS) in a phase III trial of patients with B-cell precursor ALL receiving either Rituximab in conjunction with chemotherapy to chemotherapy alone. The primary endpoint of the trial is event free survival (EFS) and all analysis for this endpoint will be on an intention to treat basis.

The incidence of cases of ALL within the age range of the trial (19-65 years) in England in the decade between 1997 and 2006 suggests that there will be 150 eligible patients per year in the UK. The aim is to recruit 80% of eligible patients, giving 120 per year. The target overall recruitment is thus 720 patients over 6 years or 840 patients over 7 years. We expect 576 or 672 of these patients to have B-lineage-ALL and be randomised equally between two treatment arms.
20% of patients are expected to be T-lineage, in which case 144 or 168 T-lineage patients will be recruited.

From implementation of protocol v.9.0, the recruitment target was increased to 811 (645 B-lineage and 157 T-lineage). This represented a further 91 patients who were to be recruited to replace patients treated prior to an urgent safety measure in April 2012 which implemented changes to the backbone chemotherapy regimen in order to address unacceptably high morbidity and mortality rates[69]. By replacing the initial 91 patients (78 B-lineage and 13 T-lineage), we aimed to ensure that 720 randomised patients were treated using the same background treatment regimen.
As of September 2016, the proportion of eligble T-lineage patients was slightly lower than originally expected (17.5%) therefore recruitment of T-lineage patients was extended to continue beyond recruitment of B-lineage patients in order to hit the T-lineage target.
Thirteen patients who had misdiagnoses (10 B-lineage and 3 T-lineage) and two T-lineage patients who were Philadelphia positive will not be included in the main analysis population and therefore these patients are also to be replaced, resulting in a recruitment target of 826 (664 B-lineage and 162 T-lineage).

17.2 Analysis of the primary objective(s)

The primary endpoint, EFS, is defined as time from randomisation to relapse or to death from any cause. Curves for the comparison of EFS will be produced using the method of Kaplan-Meier and compared using the log-rank test.

Subgroup analyses will be performed in patient groups aged ≤40 and ≥41 years. If a relationship between CD20 expression and response is found, this will be used to define positive and negative groups for subgroup analysis of EFS by treatment.

Primary objective in patients with B-lineage disease: effect of antibody on EFS

For the evaluation of additional benefit of antibody to chemotherapy in terms of EFS in patients with B-cell precursor ALL, the effect of the antibody will be tested at a two-sided significance level of 0.05, using the log rank test using all randomised patients (intention-to-treat analysis).

Primary objective in patients with T-lineage disease: effect of nelarabine on EFS

Patients with T-lineage disease enrolled in this study will be randomised between chemotherapy and the combination of Nelarabine and chemotherapy as a phase II study to determine if Nelarabine improves the EFS of patients with T-cell ALL.

17.3 Analysis of secondary objectives

Tolerability of Pegylated asparaginase in induction

Cases of hypersensitivity to pegylated asparaginase will be recorded, plus changes to Erwinia or withdrawal of asparaginase treatment.

Toxicity of non-myeloablative BMT

An early loss of 16% of patients due to induction failure or relapse is expected before transplant can be reached. About 1/3 of patients will have a matched sibling donor and about 1/2 of those without such a donor might be expected to be high risk, giving 2/3 eligible for transplant, of whom about half can be expected to be aged ≥41 years at study entry. Thus 720 or 840 recruited patients would result in 201 or 235 who might receive a non-myeloablative transplant. Based on UKALL12/E2993 data, some of these will be excluded on the basis of liver function, or other, tests, so we would expect perhaps 150 or 175 to receive the transplant. Currently the treatment related mortality (TRM) of transplant in this age group is over 40%. Unfortunately it would be impractical to randomise between standard and non-myeloablative BMT, and thus a historical comparison is necessary and results should be treated with the appropriate caution. It is expected that the reduction in toxicity will be substantial, maybe as much as halved. As there may be an increase in relapses, EFS will be the primary outcome with the current rate of 40% at 3 years post-transplant.

Palifermin randomisation – closed April 2016 as no longer clinically relevant

In the palifermin study, it was anticipated that approximately 150 or 175 patients treated with myeloablative HSCT would be randomised to evaluate the "standard dose" of palifermin vs. a single "collapsed" dose in reducing the incidence of Grades 3 or 4 mouth and throat soreness (MTS) scores. The randomisation was stratified by sex, sibling/MUD donor & precursor-B/Tlineage.

Outcome measures were:

- OMDQ
- Number of doses of MTX administered
- Acute GVHD

The randomisation was closed early in April 2016 (after 74 randomisations) when the surrender of the EU marketing authorisation for palifermin rendered the research question of its optimal dosing schedule no

longer clinically relevant. Data on patients randomised prior to April 2016 will be analysed and commented upon in the final study report.

17.3.1 Efficacy (secondary)

Secondary outcomes will be:

- Overall survival
- Complete remission rate
- Death in complete remission
- Relapse rate (actuarial incidence of relapse, excluding non-remitters and censoring at death in remission)
- Bone marrow relapse rate (actuarial incidence of relapse involving the bone marrow, excluding non-remitters and censoring at relapse at other sites and death in remission)
- Central nervous system (CNS) relapse rate (actuarial incidence of relapse involving the CNS, excluding non-remitters and censoring at relapse of other sites and death in remission)
- For the antibody randomisations only: anti-asparaginase levels and asparaginase activity prior to asparaginase infusions.
- Minimal Residual Disease at the end of Phase 1 Induction (antibody randomisation) or post-transplant

17.3.2 Safety

All grade 3 or 4 toxicities will be reported.
Patients treated before the April 2012 amendment will be excluded from the primary analyses in the final UKALL14 publication but analyses with all eligble, randomised patients will be included in a supplementary appendix.

17.4 Sample size calculations

<u>Addition of monoclonal antibody to standard induction chemotherapy in patients with precursor B-ALL</u>
The power calculations of the comparisons are made under the assumption that the 3-year EFS of the chemotherapy in this patient population (based on UKALL12/E2993 data) is approximately 40%. With 576 (or 672 with 7 years' accrual) patients randomised, and at least 6 months' follow-up after the last randomisation, we should be able to see 307 (357 over 7 years recruitment) events which would give us 84% (or 89%) power to detect an improvement in 3-year EFS from 40% to 52%.

<u>Addition of Nelarabine to standard induction chemotherapy in patients with precursor T-ALL</u>
With 144 (or 168 with 7 years' accrual) patients randomised, and at least 6 months' follow-up after the last randomisation, we should be able to see 45 events (63 over 7 years recruitment) which would give us over 86% power to detect an improvement in 3-year EFS from 50% to 75%, and about 68% (or 75% with 7 years accrual) power to detect an improvement to 70%, at a two-sided significance level of 0.05.

17.5 Power for analysis of critical secondary variables

Palifermin randomisation – closed April 2016 as no longer clinically relevant

Based on the references from Spielberger et al., and T. Shea et al., we assumed a range of incidence of Grades 3 or 4 MTS scores of 60%, 70% and 80% for the standard dose arm, and 30%, 40%, 50% and 60% for the collapsed dose arm, and a two-sided alpha level of 0.05, the table below provides the power for 75 patients per arm.

	**30%**	**40%**	**50%**	**60%**
**60%**	96%	69%	23%	N/A
**70%**	99%	96%	70%	24%
**80%**	99%	99%	97%	76%

The null hypothesis is that the incidence is the same between the standard dose and the collapsed dose arms. The alternative hypothesis is that there is a difference between the two arms. With 75 patients per arm, the study would have at least 69% power to detect a treatment difference of at least 20%.

Since the patients are hospitalised, the MTS evaluation period is relatively short (until discharge or 28 days post-transplant) and the worst score for a patient will be used for the incidence calculation, the sample size loss due to non-compliance and loss to follow up will be small and the resulting power loss will be negligible.

The palifermin randomisation was closed early in April 2016 due to lack of clinical relevance, at which time 74 patients were randomised (approximately 50% of anticipated accrual).

17.6 Interim analysis

There will be no official interim analyses for this trial, however an analysis of safety data and the main endpoints will be supplied approximately annually, in strict confidence, to the independent data monitoring committee (IDMC) In the light of these reports, the IDMC may advise the Trial Steering Committee to stop the trial if, in their view, the randomised comparisons in the trial have provided proof beyond reasonable doubt (2P<0.001) that for all or for some types of patient one treatment is clearly indicated or clearly contraindicated.

The main analyses will be performed using standard log-rank methods based on the intention to treat, i.e. all patients believed to be eligible at the time of randomisation will be included in the analysis, irrespective of protocol compliance, early relapse, etc. All analyses will assume that there may be some **quantitative** differences in the size of any treatment effects in different strata, but that there is unlikely to be any **qualitative** difference (i.e. harm in one group, benefit in another).

Final analyses will be performed when the last patient randomised has been followed up for 2 years, i.e. after all patients have finished their initial treatment.

18.0 Ethical and Regulatory Approvals

In conducting the Trial the Sponsor, UCL CTC and Sites shall also comply with all laws and statutes, as amended from time to time, applicable to the performance of clinical trials including, but not limited to:

- Applicable Good Clinical Practice requirements as outlined in Directives 2001/20/EC and 2005/28/EC and all laws implementing these in the relevant country(ies), and any amendments thereto and where applicable incorporating elements of ICH Harmonised Tripartite Guideline for Good Clinical Practice (CPMP/ICH/135/95)
- the Human Rights Act 1998
- the Data Protection Act 1998
- the Freedom of Information Act 2000
- the Human Tissue Act 2004
- the Medicines Act 1968
- the Medicines for Human Use (Clinical Trials) UK Regulations SI 2004/1031, and subsequent amendments
- Good Manufacturing Practice
- the Research Governance Framework for Health and Social Care, issued by the UK Department of Health (Second Edition 2005) or the Scottish Health Department Research Governance Framework for Health and Community Care (Second Edition 2006)

All non-UK sites shall comply with all their local laws and statutes applicable to the performance of clinical trials.

18.1 Ethical Approval

The trial will be conducted in accordance with the World Medical Associationthe Declaration of Helsinki entitled 'Ethical Principles for Medical Research Involving Human Subjects' (1996 version) and in accordance with the terms and conditions of the ethical approval given to the trial.

The trial has received a favourable opinion from the NRES Committee London – Fulham. The UCL CTC will submit Annual Progress Reports to the REC, which will commence one year from the date of ethical approval for the trial.

18.2 Regulatory Approval

A Clinical Trial Authorisation (CTA) has been granted for the trial.
The trial will be conducted at approved trial sites in accordance with the trial protocol and the terms of the CTA granted by the MHRA and other applicable regulatory authorities.

18.3 Site Approvals

The Lead Comprehensive Local Research Network (CLRN) Central and East London Comprehensive Local Research Network has given NHS permission following global governance checks. Local governance checks will be undertaken by local CLRNs associated with individual trial sites.
Evidence of approval from the Trust R&D for a trial site must be provided to UCL CTC. Sites will only be activated when all necessary local approvals for the trial have been obtained.

All non-UK sites must provide confirmation of approval of their local institution(s).

18.4 Protocol Amendments

UCL CTC will be responsible for gaining ethical and regulatory approval(s), as appropriate, for amendments made to the protocol and other trial-related documents. Once approved, UCL CTC will ensure that all amended documents are distributed to sites and CLRNs as appropriate.

Site staff will be responsible for acknowledging receipt of documents and for implementing all amendments.

18.5 Patient Confidentiality & DPA

Patient identifiable data, including initials, date of birth, hospital number and NHS/CHI number will be required for the registration/randomisation process and will be provided to UCL CTC. UCL CTC will preserve patient confidentiality and will not disclose or reproduce any information by which patients could be identified. Data will be stored in a secure manner and UCL CTC trials are registered in accordance with the Data Protection Act 1998 with the Data Protection Officer at UCL.

19.0 Sponsorship and Indemnity

19.1 Sponsor Details:

**Sponsor Name:**	**University College London**
**Sponsor Contact:**	**Director of Research Support**
**Telephone:**	+44 (0) 20 3447 9995/2178 (unit admin)
**Fax:**	+44 (0) 20 3447 9937
**Address:**	Joint Research Office
	Gower Street
	London
	WC1E 6BT

19.2 Indemnity:

University College London holds insurance to cover participants for injury caused by their participation in the clinical trial. Participants may be able to claim compensation if they can prove that UCL has been negligent. However, as this clinical trial is being carried out in a hospital, the hospital continues to have a duty of care to the participant of the clinical trial. University College London does not accept liability for any breach in the hospital's duty of care, or any negligence on the part of hospital employees. This applies whether the hospital is an NHS Trust or not. This does not affect the participant's right to seek compensation via the non-negligence route.

Participants may also be able to claim compensation for injury caused by participation in this clinical trial without the need to prove negligence on the part of University College London or another party. Participants who sustain injury and wish to make a claim for compensation should do so in writing in the first instance to the Chief Investigator, who will pass the claim to the Sponsor's Insurers, via the Sponsor's office.

Hospitals selected to participate in this clinical trial shall provide clinical negligence insurance cover for harm caused by their employees and a copy of the relevant insurance policy or summary shall be provided to University College London, upon request.

20.0 Publication Policy

All publications and presentations relating to the trial will be authorised by the TMG. The first publication of the trial results will be in the name of the TMG, if this does not conflict with the journal's policy. The TMG will form the basis of the writing committee and advise on the nature of publications. If there are named authors, these should include the Chief Investigator, Trial Coordinator, and Statistician involved in the trial. Contributing site investigators in this trial will also be acknowledged. Data from all sites will be analysed together and published as soon as possible. Participating sites may not publish trial results prior to the first publication by the TMG or without prior written consent from the TMG.

The trial data is owned by UCL CTC. Drug companies who have provided grants towards the trial will be permitted to see the draft manuscripts and make comments at least 30 days prior to submission for publication.

The EudraCT number (2009-012717-22) or the clinicaltrials.gov number (NCT01085617) will be quoted in any publications resulting from this trial.

21.0 References

1. Pui CH, Robison LL, Look AT. Acute lymphoblastic leukaemia. Lancet. 2008;371:1030-1043.
2. Pui CH, Evans WE. Treatment of acute lymphoblastic leukemia. N Engl J Med. 2006;354:166-178.
3. Rowe JM, Buck G, Burnett AK, et al. Induction therapy for adults with acute lymphoblastic leukemia: results of more than 1500 patients from the international ALL trial: MRC UKALL XII/ECOG E2993. Blood. 2005;106:3760-3767.
4. Mitchell CD, Richards SM, Kinsey SE, Lilleyman J, Vora A, Eden TO. Benefit of dexamethasone compared with prednisolone for childhood acute lymphoblastic leukaemia: results of the UK Medical Research Council ALL97 randomized trial. Br J Haematol. 2005;129:734-745.
5. de Vries MJ, Veerman AJ, Zwaan CM. Rituximab in three children with relapsed/refractory B-cell acute lymphoblastic leukaemia/Burkitt non-Hodgkin's lymphoma. Br J Haematol. 2004;125:414-415.
6. Jandula BM, Nomdedeu J, Marin P, Vivancos P. Rituximab can be useful as treatment for minimal residual disease in bcr-abl-positive acute lymphoblastic leukemia. Bone Marrow Transplant. 2001;27:225-227.
7. Ueda T, Miyawaki S, Asou N, et al. Response-oriented individualized induction therapy with six drugs followed by four courses of intensive consolidation, 1 year maintenance and intensification therapy: the ALL90 study of the Japan Adult Leukemia Study Group. Int J Hematol. 1998;68:279-289.
8. Thomas DA, O'Brien S, Jorgensen JL, et al. Prognostic significance of CD20 expression in adults with de novo precursor B-lineage acute lymphoblastic leukemia. Blood. 2008.
9. Dworzak MN, Schumich A, Printz D, et al. CD20 up-regulation in pediatric B-cell precursor acute lymphoblastic leukemia during induction treatment: setting the stage for anti-CD20 directed immunotherapy. Blood. 2008;112:3982-3988.
10. Kurtzberg J, Ernst TJ, Keating MJ, et al. Phase I study of 506U78 administered on a consecutive 5-day schedule in children and adults with refractory hematologic malignancies. J Clin Oncol. 2005;23:3396-3403.
11. Berg SL, Blaney SM, Devidas M, et al. Phase II study of nelarabine (compound 506U78) in children and young adults with refractory T-cell malignancies: a report from the Children's Oncology Group. J Clin Oncol. 2005;23:3376-3382.
12. Deangelo DJ, Yu D, Johnson JL, et al. Nelarabine induces complete remissions in adults with relapsed or refractory T-lineage acute lymphoblastic leukemia or lymphoblastic lymphoma: cancer and leukemia group B study 19801. Blood. 2007.
13. Avramis VI, Panosyan EH. Pharmacokinetic/pharmacodynamic relationships of asparaginase formulations: the past, the present and recommendations for the future. Clin Pharmacokinet. 2005;44:367-393.
14. Avramis VI, Spence SA. Clinical pharmacology of asparaginases in the United States: asparaginase population pharmacokinetic and pharmacodynamic (PK-PD) models (NONMEM) in adult and pediatric ALL patients. J Pediatr Hematol Oncol. 2007;29:239-247.
15. Wetzler M, Sanford BL, Kurtzberg J, et al. Effective asparagine depletion with pegylated asparaginase results in improved outcomes in adult acute lymphoblastic leukemia: Cancer and Leukemia Group B Study 9511. Blood. 2007;109:4164-4167.
16. Graham ML. Pegaspargase: a review of clinical studies. Adv Drug Deliv Rev. 2003;55:1293-1302.
17. Silverman LB, Gelber RD, Dalton VK, et al. Improved outcome for children with acute lymphoblastic leukemia: results of Dana-Farber Consortium Protocol 91-01. Blood. 2001;97:1211-1218.
18. Avramis VI, Sencer S, Periclou AP, et al. A randomized comparison of native Escherichia coli asparaginase and polyethylene glycol conjugated asparaginase for treatment of children with newly diagnosed standard-risk acute lymphoblastic leukemia: a Children's Cancer Group study. Blood. 2002;99:1986-1994.
19. Larson RA, Dodge RK, Burns CP, et al. A five-drug remission induction regimen with intensive consolidation for adults with acute lymphoblastic leukemia: cancer and leukemia group B study 8811. Blood. 1995;85:2025-2037.
20. Lazarus HM, Richards SM, Chopra R, et al. Central nervous system involvement in adult acute lymphoblastic leukemia at diagnosis: results from the international ALL trial MRC UKALL XII/ECOG E2993. Blood. 2006;108:465-472.
21. Pui CH. Central nervous system disease in acute lymphoblastic leukemia: prophylaxis and treatment. Hematology Am Soc Hematol Educ Program. 2006:142-146.

22. Hill FG, Richards S, Gibson B, et al. Successful treatment without cranial radiotherapy of children receiving intensified chemotherapy for acute lymphoblastic leukaemia: results of the risk-stratified randomized central nervous system treatment trial MRC UKALL XI (ISRC TN 16757172). Br J Haematol. 2004;124:33-46.
23. Tubergen DG, Gilchrist GS, O'Brien RT, et al. Prevention of CNS disease in intermediate-risk acute lymphoblastic leukemia: comparison of cranial radiation and intrathecal methotrexate and the importance of systemic therapy: a Childrens Cancer Group report. J Clin Oncol. 1993;11:520-526.
24. Annino L, Vegna ML, Camera A, et al. Treatment of adult acute lymphoblastic leukemia (ALL): long-term follow-up of the GIMEMA ALL 0288 randomized study. Blood. 2002;99:863-871.
25. Kantarjian H, Thomas D, O'Brien S, et al. Long-term follow-up results of hyperfractionated cyclophosphamide, vincristine, doxorubicin, and dexamethasone (Hyper-CVAD), a dose-intensive regimen, in adult acute lymphocytic leukemia. Cancer. 2004;101:2788-2801.
26. Sancho JM, Ribera JM, Oriol A, et al. Central nervous system recurrence in adult patients with acute lymphoblastic leukemia: frequency and prognosis in 467 patients without cranial irradiation for prophylaxis. Cancer. 2006;106:2540-2546.
27. Goldstone AH, Richards SM, Lazarus HM, et al. In adults with standard-risk acute lymphoblastic leukemia, the greatest benefit is achieved from a matched sibling allogeneic transplantation in first complete remission, and an autologous transplantation is less effective than conventional consolidation/maintenance chemotherapy in all patients: final results of the International ALL Trial (MRC UKALL XII/ECOG E2993). Blood. 2008;111:1827-1833.
28. Fielding AK, Richards SM, Chopra R, et al. Outcome of 609 adults after relapse of acute lymphoblastic leukemia (ALL); an MRC UKALL12/ECOG 2993 study. Blood. 2007;109:944-950.
29. Blume KG, Forman SJ, O'Donnell MR, et al. Total body irradiation and high-dose etoposide: a new preparatory regimen for bone marrow transplantation in patients with advanced hematologic malignancies. Blood. 1987;69:1015-1020.
30. Blume KG, Forman SJ, Snyder DS, et al. Allogeneic bone marrow transplantation for acute lymphoblastic leukemia during first complete remission. Transplantation. 1987;43:389-392.
31. Marks DI, Forman SJ, Blume KG, et al. A Comparison of Cyclophosphamide and Total Body Irradiation with Etoposide and Total Body Irradiation as Conditioning Regimens for Patients Undergoing Sibling Allografting for Acute Lymphoblastic Leukemia in First or Second Complete Remission. Biol Blood Marrow Transplant. 2006;12:438-453.
32. Spielberger R, Stiff P, Bensinger W, et al. Palifermin for oral mucositis after intensive therapy for hematologic cancers. N Engl J Med. 2004;351:2590-2598.
33. Blazar BR, Weisdorf DJ, Defor T, et al. Phase 1/2 randomized, placebo-control trial of palifermin to prevent graft-versus-host disease (GVHD) after allogeneic hematopoietic stem cell transplantation (HSCT). Blood. 2006;108:3216-3222.
34. Nash RA, Pepe MS, Storb R, et al. Acute graft-versus-host disease: analysis of risk factors after allogeneic marrow transplantation and prophylaxis with cyclosporine and methotrexate. Blood. 1992;80:1838-1845.
35. Kumar S, Wolf RC, Chen MG, et al. Omission of day +11 methotrexate after allogeneic bone marrow transplantation is associated with increased risk of severe acute graft-versus-host disease. Bone Marrow Transplant. 2002;30:161-165.
36. Baron F, Storb R. Current roles for allogeneic hematopoietic cell transplantation following nonmyeloablative or reduced-intensity conditioning. Clin Adv Hematol Oncol. 2005;3:799-819.
37. Martino R, Giralt S, Caballero MD, et al. Allogeneic hematopoietic stem cell transplantation with reduced-intensity conditioning in acute lymphoblastic leukemia: a feasibility study. Haematologica. 2003;88:555-560.
38. Hamaki T, Kami M, Kanda Y, et al. Reduced-intensity stem-cell transplantation for adult acute lymphoblastic leukemia: a retrospective study of 33 patients. Bone Marrow Transplant. 2005;35:549-556.
39. Piccaluga PP, Martinelli G, Malagola M, et al. Anti-leukemic and anti-GVHD effects of campath-1H in acute lymphoblastic leukemia relapsed after stem-cell transplantation. Leuk Lymphoma. 2004;45:731-733.
40. Chakraverty R, Peggs K, Chopra R, et al. Limiting transplantation-related mortality following unrelated donor stem cell transplantation by using a nonmyeloablative conditioning regimen. Blood. 2002;99:1071-1078.

41. Delgado J, Thomson K, Russell N, et al. Results of alemtuzumab-based reduced-intensity allogeneic transplantation for chronic lymphocytic leukemia: a British Society of Blood and Marrow Transplantation Study. Blood. 2006;107:1724-1730.
42. Morris E, Thomson K, Craddock C, et al. Outcomes after alemtuzumab-containing reduced-intensity allogeneic transplantation regimen for relapsed and refractory non-Hodgkin lymphoma. Blood. 2004;104:3865-3871.
43. Stein A, O'Donnell M, Snyder DS, et al. Reduced-Intensity Stem Cell Tansplantation for high-risk acute lymphoblastic leukaemia. Biology of Blood and Marrow Transplantation. 2007;13:134.
44. Dahlke J, Kroger N, Zabelina T, et al. Comparable results in patients with acute lymphoblastic leukemia after related and unrelated stem cell transplantation. Bone Marrow Transplant. 2006;37:155-163.
45. Kiehl MG, Kraut L, Schwerdtfeger R, et al. Outcome of allogeneic hematopoietic stem-cell transplantation in adult patients with acute lymphoblastic leukemia: no difference in related compared with unrelated transplant in first complete remission. J Clin Oncol. 2004;22:2816-2825.
46. Moorman AV, Harrison CJ, Buck GA, et al. Karyotype is an independent prognostic factor in adult acute lymphoblastic leukemia (ALL): analysis of cytogenetic data from patients treated on the Medical Research Council (MRC) UKALLXII/Eastern Cooperative Oncology Group (ECOG) 2993 trial. Blood. 2007;109:3189-3197.
47. Roy A, Bradburn M, Moorman AV, et al. Early response to induction is predictive of survival in childhood Philadelphia chromosome positive acute lymphoblastic leukaemia: results of the Medical Research Council ALL 97 trial. Br J Haematol. 2005;129:35-44.
48. Schmitz N, Bacigalupo A, Hasenclever D, et al. Allogeneic bone marrow transplantation vs filgrastim-mobilised peripheral blood progenitor cell transplantation in patients with early leukaemia: first results of a randomised multicentre trial of the European Group for Blood and Marrow Transplantation. Bone Marrow Transplant. 1998;21:995-1003.
49. Iwamoto S, Mihara K, Downing JR, Pui CH, Campana D. Mesenchymal cells regulate the response of acute lymphoblastic leukemia cells to asparaginase. J Clin Invest. 2007;117:1049-1057.
50. Garderet L, Labopin M, Gorin NC, et al. Patients with acute lymphoblastic leukaemia allografted with a matched unrelated donor may have a lower survival with a peripheral blood stem cell graft compared to bone marrow. Bone Marrow Transplant. 2003;31:23-29.
51. Moppett J, Burke GA, Steward CG, Oakhill A, Goulden NJ. The clinical relevance of detection of minimal residual disease in childhood acute lymphoblastic leukaemia. J Clin Pathol. 2003;56:249-253.
52. Cave H, van der Werff ten Bosch J, Suciu S, et al. Clinical significance of minimal residual disease in childhood acute lymphoblastic leukemia. European Organization for Research and Treatment of Cancer--Childhood Leukemia Cooperative Group. N Engl J Med. 1998;339:591-598.
53. Bruggemann M, Raff T, Flohr T, et al. Clinical significance of minimal residual disease quantification in adult patients with standard-risk acute lymphoblastic leukemia. Blood. 2006;107:1116-1123.
54. Hunault-Berger M, Chevallier P, Delain M, et al. Changes in antithrombin and fibrinogen levels during induction chemotherapy with L-asparaginase in adult patients with acute lymphoblastic leukemia or lymphoblastic lymphoma. Use of supportive coagulation therapy and clinical outcome: the CAPELAL study. Haematologica. 2008;93:1488-1494.
55. Mullighan CG, Goorha S, Radtke I, et al. Genome-wide analysis of genetic alterations in acute lymphoblastic leukaemia. Nature. 2007;446:758-764.
56. Mullighan CG, Su X, Zhang J, et al. Deletion of IKZF1 and prognosis in acute lymphoblastic leukemia. N Engl J Med. 2009;360:470-480.
57. Paulsson K, Cazier JB, Macdougall F, et al. Microdeletions are a general feature of adult and adolescent acute lymphoblastic leukemia: Unexpected similarities with pediatric disease. Proc Natl Acad Sci U S A. 2008;105:6708-6713.
58. Ferrando AA, Neuberg DS, Staunton J, et al. Gene expression signatures define novel oncogenic pathways in T-cell acute lymphoblastic leukemia. Cancer Cell. 2002;1:75-87.
59. Strefford JC, van Delft FW, Robinson HM, et al. Complex genomic alterations and gene expression in acute lymphoblastic leukemia with intrachromosomal amplification of chromosome 21. Proc Natl Acad Sci U S A. 2006;103:8167-8172.
60. Sulong S, Moorman AV, Irving JA, et al. A comprehensive analysis of the CDKN2A gene in childhood acute lymphoblastic leukemia reveals genomic deletion, copy number neutral loss of heterozygosity, and association with specific cytogenetic subgroups. Blood. 2009;113:100-107.

61. Parker H, An Q, Barber K, et al. The complex genomic profile of ETV6-RUNX1 positive acute lymphoblastic leukemia highlights a recurrent deletion of TBL1XR1. Genes Chromosomes Cancer. 2008;47:1118-1125.

62. Patel B, Rai L, Buck G, et al. Minimal residual disease is a significant predictor of treatment failure in non T-lineage adult acute lymphoblastic leukaemia: final results of the internationaltrial UKALL XII/ECOG2993. Br J Haematol. 2010;148:80–89.

63. Mohty M, Labopin M, Tabrizzi R, et al. Reduced intensity conditioning allogeneic stem cell transplantation for adult patients with acute lymphoblastic leukemia: a retrospective study from the European Group for Blood and Marrow Transplantation. Haematologica. 2008;93:303-306.

64. Nowak-Gottl U, Ahlke E, Fleischhack G, et al. Thromboembolic events in children with acute lymphoblastic leukemia (BFM protocols): prednisone versus dexamethasone administration. Blood. 2003;101:2529-2533

65. Igarashi S, Manabe A, Ohara A, et al. No advantage of dexamethasone over prednisolone for the outcome of standard- and intermediate-risk childhood acute lymphoblastic leukemia in the Tokyo Children's Cancer Study Group L95-14 protocol. J Clin Oncol. 2005;23:6489-6498.

66. Habermann TM, Weller E, Morrison VA, et al. Rituximab-CHOP versus CHOP alone or with maintenance rituximab in older patients with diffuse large B-cell lymphoma. J Clin Onc.2006;24:3121-3127

67. Marks DI, Woo KA, Zhong X, Appelbaum FR, Bachanova V, Marker JN, Brunstein CG, Gibson J, Kebriaei P, Lazarus HM, Olsson R, Perlaes MA, Pidala J, Savani B, Rocha V & Eapen M. Unrelated umbilical cord blood transplant for adult acute lymphoblastic leukaemia in first and second complete remission: a comparison with allografts from adult unrelated donors. Haematologica 2014; 99:322-8

68. Charrin C, Thomas X, Ffrench M, Le Q-H, Andrieux J, Mozziconacci M-J, Laï J-L, Bilhou-Nabera C, Michaux L, Bernheim A, Bastard C, Mossafa H, Perot C, Maarek O, Boucheix C, Lheritier V, Delannoy A, Fière D & Dastugue N. A report from the LALA-94 and LALA-SA groups on hypodiploidy with 30 to 39 chromosomes and near-triploidy: 2 possile expressions of a sole entity conferring poor prognosis in adulte acute lymphoblastic leukemia (ALL). Blood 2004; 104:2444-2451

69. Patel B, Kirkwood AA, Dey A, Marks DI, McMillan AK, Menne TF, Micklewright L, Patrick P, Purnell S, Rowntree CJ, Smith P, Fielding AK. Pegylated-asparaginase during induction therapy for adult acute lymphoblastic leukaemia: toxicity data from the UKALL14 trial. Leukemia. 2016. Sep 9; 1-7

Appendix 1: 'Registration only' sub-study

1. Background

The UKALL14 B-cell randomisation (standard induction ± Rituximab) closed in July 2017, having reached its recruitment target of 664 patients.
From implementation of protocol v11.0 onwards, newly-diagnosed B-cell patients have been given the opportunity to enter a 'registration only' sub-study, whereby they will receive standard ALL treatment as per their local clinican's choice, while contributing minimal data and samples (MRD and optional constitutional DNA) to address laboratory aims.
The UKALL14 T-cell randomisation (standard induction ± Nelarabine) is due to close mid 2018 when it has reached its target of 162 patients. From implementation of protocol v12.0 onwards or completion of the T-cell randomisation (whichever is later), newly-diagnosed T-cell patients will also be given the opportunity to enter the 'registration only' sub-study.

The sub-study is not an extension to the B1 or T1 arm of the randomised study. 'Registration only' patients will not be required to follow a protocol-defined treatment regimen, and any medication given as part of standard treatment will not be considered as an IMP.

The 'registration only' sub-study is purely observational and intended to address laboratory questions, allow patients to continue to contribute to leukaemia studies, and collect basic information about patient outcome to correlate with lab findings. The laboratory aims are linked to our ongoing peer-reviewed funded research programme including "personalising therapy for adults with ALL" and "defining a T-cell signature which predicts response to immune therapy in ALL".

2. Aims of the 'registration only' sub-study

Data and samples collected on 'registration only' patients will be used to address the following laboratory aims:

- To characterise the genomic landscape of adult ALL
- To investigate the clonal origins of relapsed ALL
- To define a T-cell signature which predicts response to allo-HCT
- To develop global risk models in adult ALL that integrate demographic, genetic and response information.

3. Consent of 'registration only' patients

Sites are responsible for assessing a patient's capacity to give informed consent.
Patients must consent in order to take part in the 'registration only' sub-study. The current approved version of the ***'Registration only' sub-study*** PIS and ICF must be used for this purpose. Please note that this is a different PIS/ICF to that used for randomised patients.

‘Registration only’ patients should also be asked to provide an optional buccal swab for constitutional DNA extraction. The ***Additional Genetic Testing (Buccal Swab)*** PIS and ICF should be used for this purpose. Patients may still enter the ‘registration only’ sub-study even if they do not wish to provide a buccal swab.

The consent process must be undertaken by appropriately trained staff, allocated that duty on the trial delegation log, in accordance with the requirements laid out in section 4.0 of the trial protocol.

A minimum of twenty-four hours should be allowed for the patient to consider and discuss participation in the sub-study. However, if this is not possible (e.g. patient needs to start treatment urgently), the patient may consent on the same day as being given the patient information sheet, provided the member of staff taking consent is satisfied that the patient understands the trial and its implications. A member of the research team at the hospital should then have a further discussion with the patient in the following days to confirm their ongoing willingness to participate in the sub-study. This should be recorded in the source data.

Site staff are responsible for:

- checking that the current approved version of the patient information sheet and consent form are used
- checking information on the consent form is complete and legible
- checking that the patient has completed/initialled all relevant sections and signed and dated the form
- checking that an appropriate member of staff has countersigned and dated the consent form to confirm that they provided information to the patient
- checking that an appropriate member of staff has made dated entries in the patient’s medical notes relating to the informed consent process (i.e. information given, consent signed, etc.)
- giving the patient a copy of their signed consent form and patient information sheet
- documenting and providing details of the consent process to UCL CTC
- following registration: adding the patient trial number to all copies of the consent forms, which should be filed in the patient’s medical notes and investigator site file.

The right of the patient to refuse to participate in the trial without giving reasons must be respected. All patients are free to withdraw at any time from the sub-study without giving reasons and without prejudicing further treatment. Any patient’s withdrawal of consent from the sub-study must be explicitly documented in the source documents and UCL CTC informed.

4. Pre-registration investigations for ‘registration only’ patients

Pre-treatment evaluations should be carried out as per local policy.

5. Eligibility for the sub-study

Patients entering the 'registration only' sub-study are expected to meet broadly the same criteria as patients entered into the randomised UKALL14 trial:

5.1 Inclusion criteria

a) Subjects aged ≥25 and ≤65 years with acute lymphoblastic leukaemia (≥19 and ≤65 years old if Philadelphia chromosome positive ALL)
b) Newly diagnosed, previously untreated ALL (a steroid pre-phase is permitted and can be started prior to registration)
c) Written, informed consent

5.2 Exclusion criteria

a) Known HIV infection
b) Known history of hepatitis B infection
c) Known history of hepatitis C infection
d) Pregnant or lactating women
e) Blast transformation of CML
f) Mature B-cell leukaemia (i.e. Burkitt's lymphoma t(8;14)(q24;q32) and variant c-myc translocations e.g. t(2;8)(p12;q24), t(8;22)(q24;q11)

Hepatitis testing remains mandatory for those patients where sites have chosen to obtain rituximab off licence for CD20 expressing B-lineage ALL, as per national MHRA guidance from December 2013:

"*Advice for healthcare professionals:*

- *Screening for hepatitis B virus is now recommended in all patients (not only those at risk of this infection) before starting treatment for all indications.*
- *Patients with active hepatitis B disease should not be treated with rituximab.*
- *A patient with positive serology for hepatitis B virus should be referred to a specialist in liver disease before starting treatment with rituximab. During treatment, these patients should be monitored and managed to prevent reactivation of the virus."*

The ALL working group has submitted an application to NHS England to obtain baseline funding for rituximab in the setting of CD20-positive pre B-ALL but a decision on this proposal is not expected until the final quarter of 2018.

6. Registration Procedure for 'registration only' sub-study

- Patient registration will be performed centrally at UCL CTC and must be performed prior to commencement of treatment (a steroid pre-phase is permitted and can be started prior to study entry).
- Following completion of pre-treatment evaluations at site, a registration form must be fully completed and faxed to UCL CTC. This will be used to confirm patient eligibility. N.B. If the site is unable to fax, registration forms may be sent by email. If emailing forms, patient identifiable

information from the form (e.g. NHS number, day and month of birth) must be redacted before it is emailed to ctc.ukall14@ucl.ac.uk. The identifiable information must be provided to UCL CTC via telephone so that UCL CTC can transcribe this information on to the form. The un-redacted form must then be posted to UCL CTC, and a copy kept in the patient file at site.

- A trial number will be assigned and details added to the form. UCL CTC will fax confirmation of the patient's inclusion in the trial and their trial number to the main contact. Trial numbers will take the following format:
  - 'Registration only' B-cell patients: 14-3-xxx
  - 'Registration only' T-cell patients: 14-4-xxx

Registration fax number:	020 7679 9861
Office hours:	09:00 to 17:00 (UK Time)<br>Monday to Friday (excluding public holidays)

Once a patient has been registered onto the sub-study, they must be provided with a copy of their signed consent form (if not given at time of consent).

7. Treatment for 'registration only' patients

'Registration only' patients will receive standard ALL treatment as per their local clinician's choice. Therefore the drugs used to treat these patients are not regarded as IMPs or non-IMPs for the purposes of the trial. All treatment will be sourced from local hospital stock.
If pegylated asparaginase, rituximab or nelarabine are given, they must be sourced from hospital stock and not from clinical trial supply.

Drug accountability is not required for treatment administered to 'registration only' patients.

8. Assessments and data collection

8.1 Assessment time points

A limited dataset will be collected for 'registration only' patients. Data will be collected at the following timepoints:

	'Registration only' patients
Initial diagnosis & pre treatment	X
Post induction phase 1 (at count recovery*)	X
Post induction phase 2 (at count recovery*)	X
After intensification	X
After last cycle of consolidation, before start of maintenance	If given
Every 3 months during maintenance	If given

therapy	
Day 100 post-transplant	If given
Annual follow up	X<br>From anniversary of completion of maintenance, transplant or early withdrawal from treatment
Relapse	X
Death	X

Data should be submitted within 1 month of the patient being seen, with the exception of the initial diagnosis and pre-treatment data, which will be submitted at the time of registration, and the relapse and death forms, which should be submitted as soon as possible after becoming aware of the death.

8.2 Routine Clinical and Laboratory Assessments during treatment

'Registration only' patients should be assessed at the end of each phase of treatment that they receive (see table above for timepoints). Investigations should be performed as per local practice and the following information will be collected, where available:

- Treatment given during the phase
- Survival status
- Remission status (% bone marrow blasts).

Sites are encouraged to collect as much of the information listed above as possible, however there is no obligation to carry out investigations in excess of standard of care. If a specific assessment is not done as standard at site at a given time point, it should be marked as not done (ND) on the case report form.

8.3 Schedule of testing for MRD and correlative science

MRD samples will be sent to the central laboratory at UCL Cancer Institute for 'registration only' patients to ensure standardised (standard of care) MRD testing and allow samples to be used for the laboratory aims of the sub-study. MRD for 'registration only' patients will be charged at the same rate as MRD for randomised patients (see Appendix 8 for details).

	**Specimens for local assessment**	**Specimens for correlative studies to be sent to central laboratory** (Samples to be sent to UCL Cancer Institute. See appx. 8 for UCL Cancer institute lab address)*****
**At Diagnosis**	As per local practice.<br><br>A copy of the local cytogenetics/ molecular assessment results should be sent to the Leukaemia Research Cytogenetics Group (LRCG) at Newcastle University. (Please refer to appx. 7 for details of the LRCG).	Bone marrow 3-5ml in EDTA (OR peripheral blood 30-50ml in EDTA if bone marrow not available) for identification of MRD markers.<br>BCR-ABL status will also be checked.<br><br>If patient has consented: Buccal swab for constitutional DNA extraction<br><br>The final date for baseline samples to be received at the MRD laboratory for the trial is 29th October 2021.

**At recovery** post Phase 1**	Bone marrow aspirate for remission assessment locally. Same day local FBC to determine peripheral count.	Bone marrow aspirate: 3-5ml in EDTA for MRD assessment - **Result not typically reported to site however can be made available upon special request** (Appx. 8) (IgH/TCR rearrangements for Ph-ALL, BCR-ABL for Ph+ALL)
**At recovery** post Phase 2**	Bone marrow aspirate for remission assessment locally. Same day local FBC to determine peripheral count.	Bone marrow aspirate: 3-5ml in EDTA for MRD assessment. - **Result reported to site within 10 working days of receipt of sample** (Appx. 8) (IgH/TCR rearrangements for Ph-ALL, BCR-ABL for Ph+ALL).
**At Relapse**	As per local practice	3-5ml bone marrow aspirate in EDTA (OR peripheral blood 30-50ml in EDTA if WCC > 30x 10^9/l).

***Please refer to Appendix 8 for guidelines for sending specimens to the central laboratory**
** "Recovery" is defined as neutrophils >0.75 x 10^9/l, platelets >75 x 10^9/l.

8.4 Assessments during follow up

Patients will be followed up annually for overall and progression-free survival until the end of the study or death.
Investigations should be performed as per local practice and suspected relapses investigated as clinically indicated. Confirmed relapses and deaths should be reported promptly.

8.5 Transfer of 'registration only' patients to another hospital

If the management of a 'registration only' patient is transferred to another hospital at any time during treatment or follow up, the procedures outlined in Protocol section 14.3 (Moving) must be followed to ensure that UCL CTC has an up to date record of which hospital is responsible for the patient's care and data submission.

- A centre transfer form must be completed and submitted to UCL CTC
- Any data pertaining to sub-study visits up to the point of transfer must be completed and submitted to UCL CTC
- A copy of the patient's CRFs up to the point of transfer must be provided to the new centre.

9. Pharmacovigilance

The 'registration only' sub-study is a purely observational sub-study where patients will receive standard ALL treatment as per their local clinician's choice and any medication given as part of standard treatment will not be considered as an IMP. Therefore, Clinical Trial Regulations do not apply to 'registration only' patients and AEs, SAEs or pregnancies do not need to be reported to the CTC. Clinicians should report adverse reactions and serious adverse reactions to the MHRA via the Yellow Card scheme, as per routine post-marketing surveillance.

10. Sample size

Recruitment to the 'registration only' sub-study will run from December 2017 to 29th October 2021 inclusive. We expect approximately 173 B-cell 'registration only' patients and 36 T-cell 'registration only' patients to be

recruited during this recruitment period.These numbers are a projection only and the final patient number may be higher or lower than these figures when recruitment to the trial ends on 31st December 2021. 'Registration only' patients will not be included in the primary analyses and final publication for UKALL14, but may be combined for further publications and scientific correlative studies.

Appendix 2: Abbreviations

**AE**	Adverse event
**ALL**	Acute lymphoblastic leukaemia
**ALT**	Alanine transaminase
**ANC**	Absolute neutrophil count
**AR**	Adverse reaction
**AST**	Aspartate aminotransferase
**AT**	Antithrombin
**APTT**	Activated partial thromboplastin time
**AVN**	Avascular necrosis
**BMT**	Bone marrow transplant
**CBT**	Cord blood transplant
**CI**	Chief Investigator
**CIBMTR**	Center for International Blood and Marrow Transplant Research
**CLS**	Country Lead Site
**CMV**	Cytomegalovirus
**CNA**	Copy number alteration
**CNS**	Central nervous system
**CR**	Complete response
**CRF**	Case report form
**CrCl**	Creatinine clearance
**CT**	Computerised tomography
**CTA**	Clinical Trial Authorisation
**CTAAC**	Clincal Trials Advisory & Awards Committee
**CTCAE**	see NCI CTCAE
**CTSA**	Clinical Trial Site Agreement
**DFS**	Disease free survival
**DLI**	Donor lymphocyte infusion
**DPA**	Data Protection Act
**DSUR**	Development Safety Update Report
**ECG**	Electrocardiogram
**ECOG**	Eastern Cooperative Oncology Group
**EDTA**	Ethyenediaminetetraacteic acid
**EFS**	Event free survival
**EU**	European Union
**EudraCT**	European Clinical Trials Database
**FISH**	Fluorescent in situ hybridisation
**FBC**	Full blood count
**G-CSF**	Granulocyte colony stimulating factor

**GVHD**	Graft versus host disease
**GFR**	Glomerular filtration rate
**GVL**	Graft versus lymphoma/Graft versus leukaemia
**HSCT**	Hematopoietic stem cell transplantation
**Hb**	Haemoglobin
**HLA**	Human leukocyte antigen
**HSv**	Herpes simplex virus
**ICH GCP**	International Conference of Harmonisation-Good Clinical Practice
**IDMC**	Independent Data Monitoring Committee
**IMP**	Investigational medicinal product
**ISRCTN**	International Standard Randomised Controlled Trial Number
**IV**	Intravenous
**LDH**	Lactate dehydrogenase
**LFT**	Liver function tests
**LMWH**	Low molecular weight heparin
**MUD**	Matched unrelated donor
**MRC**	Medical Research Council
**MREC**	Multicentre Research Ethics Committee
**MRD**	Minimal residual disease
**MHRA**	Medicines and Healthcare products Regulatory Agency
**MP**	Mercaptopurine
**MTX**	Methotrexate
**NCI CTCAE**	National Cancer Institute Common Terminology Criteria for Adverse Events
**NCRI**	National Cancer Research Institute
**NCRN**	National Cancer Research Network
**NIMP**	Non-investigational medicinal product
**NRES**	National Research Ethics Service
**OMDQ**	Oral Mucositis Daily Questionnaire
**OS**	Overall survival
**PCP**	Pneumocystis pneumonia
**PD**	Progressive disease
**PFS**	Progression free survival
**Ph**	Philadelphia chromosome / t(9;22)(q34;q11) / BCR-ABL1
**PI**	Principal Investigator
**PO**	By mouth
**PR**	Partial response
**RIC-SCT**	Reduced intensity conditioned stem cell transplant
**SAE**	Serious adverse event
**SAR**	Serious adverse reaction
**SCT**	Stem cell transplant

**SD**	Stable disease
**SPC**	Summary of Product Characteristics
**SUSAR**	Suspected unexpected serious adverse reaction
**TBI**	Total body irradiation
**TRM**	Transplant related mortality
**TMF**	Trial master file
**TMG**	Trial Management Group
**TSC**	Trial Steering Committee
**UCL CTC**	CR UK and UCL Cancer Trials Centre
**U&E**	Urea and electrolyte
**ULN**	Upper limit of normal
**VTE**	Venous thromboembolism
**VZV**	Varicella zoster virus
**WBC**	White blood cells
**WCC**	White cell count

Appendix 3: IMP Information

Up to date Summaries of Product Characteristics can be found for each of the IMPs via the Electronic Medicines Compendium website: www.medicines.org.uk/emc.

It is recommended that sites follow local guidelines for dose adjustments for obese patients.

Rituximab (Mabthera)

Formulation and storage

Chemical Name:	Rituximab
Other Names:	Mabthera®
Physical Characteristics:	Concentrate for solution for infusion. Clear, colourless liquid

Description

Rituximab is a genetically engineered chimeric mouse/human monoclonal antibody representing a glycosylated immunoglobulin with human IgG1 constant regions and murine light-chain and heavy-chain variable region sequences. The antibody is produced by mammalian (Chinese hamster ovary) cell suspension culture and purified by affinity chromatography and ion exchange, including specific viral inactivation and removal procedures.

Rituximab is supplied as a clear, colourless liquid. It is a concentrate for solution for infusion. Rituximab comes in 500mg and 100mg vials containing concentrate for solution for infusion. Each solution contains 10mg/ml of rituximab.

Rituximab should be stored in a refrigerator (2–8°C). Keep the container in the outer carton in order to protect from light.

The prepared infusion solution of Rituximab is physically and chemically stable for 24 hours at 2–8°C and subsequently 12 hours at room temperature.

Please refer to the SmPC for more information.

Administration

Aseptically withdraw the necessary amount of MabThera, and dilute to a calculated concentration of 1 to 4 mg/ml rituximab into an infusion bag containing sterile, pyrogen-free sodium chloride 9 mg/ml (0.9%) solution for injection or 5% Glucose in water. For mixing the solution, gently invert the bag in order to avoid foaming. Care must be taken to ensure the sterility of prepared solutions. Since the medicinal product does not contain any anti-microbial preservative or bacteriostatic agents, aseptic technique must be observed. Parenteral medicinal products should be inspected visually for particulate matter and discoloration prior to administration. Any unused product or waste material should be disposed of in accordance with local requirements.

The prepared Rituximab solution should be administered as an intravenous infusion through a dedicated line. It should not be administered as an intravenous push or bolus.

Rituximab infusions should be administered under the close supervision of an experienced physician, and in an environment where full resuscitation facilities are immediately available.
Premedication consisting of an anti-pyretic and an antihistaminic, e.g. paracetamol and diphenhydramine, should always be administered before each infusion of Rituximab. Premedication with glucocorticoids should also be considered.
The recommended initial rate for infusion is 50 mg/hr; after the first 30 minutes, it can be escalated in 50 mg/hr increments every 30 minutes, to a maximum of 400 mg/hr.
Subsequent doses of MabThera can be infused at an initial rate of 100 mg/hr, and increased by 100 mg/hr increments at 30 minutes intervals, to a maximum of 400 mg/hr.
For second and subsequent doses, it is acceptable to give Rituximab according to the escalated infusion protocol – to give 20% of the infusion over 30 minutes, with the remaining 80% given over an hour.
Patients should be closely monitored for the onset of cytokine release syndrome. Patients who develop evidence of severe reactions, especially severe dyspnoea, bronchospasm or hypoxia should have the infusion interrupted immediately. Patients should be evaluated for evidence of tumour lysis syndrome including appropriate laboratory tests and, for pulmonary infiltration, with a chest x-ray. In all patients, the infusion should not be restarted until complete resolution of all symptoms, and normalisation of laboratory values and chest x-ray findings. At this time, the infusion can be initially resumed at not more than one-half the previous rate. If the same severe adverse reactions occur for a second time, the decision to stop the treatment should be seriously considered on a case by case basis.

Mild or moderate infusion-related reactions usually respond to a reduction in the rate of infusion. The infusion rate may be increased upon improvement of symptoms.

A dose of 375mg/m^2 should be given by IV on days 3,10,17 & 24 of Phase 1 Induction Therapy.

Pegylated Asparaginase (Oncaspar)

Please note: Patients with Philadelphia positive ALL should NOT be given pegylated asparaginase.

Formulation and storage

Chemical Name:	Pegylated Asparaginase
Other Names:	Pegaspargase, Oncaspar ®
Physical Characteristics:	Solution for injection

Description

Oncaspar® is a medicinal product (cytostatic agent) which lowers the L-asparaginase level in the tumour cells so that the protein synthesis in these cells is inhibited.

Administration

When administered intravenously Oncaspar® should be given over a period of 1-2 hours in 100ml sodium chloride 0.9% or glucose 5% into a free flowing infusion.

Oncaspar® is available in packs with:

1 vial (type I glass) containing 5 ml ready-to-use solution for injection [N1] (Geman labelling) or

1 vial (type I glass) containing 5 ml ready-to-use solution for injection [N1] (Geman-English labelling).

Not all pack sizes may be marketed.

1 vial contains 3750 I.U. pegylated asparaginase (equivalent to 750 I.U./ml), in a clear colourless phosphate-buffered sodrium chloride solution, pH 7.3.

Oncaspar should be stored in a refrigerator (2–8°C), and should not be frozen or shaken. Discard any drug that remains unused. Do not use if the solution is cloudy or a precipitate has formed.

Please refer to the SmPC for more information.

Nelarabine (Atriance)

Formulation and storage

Chemical Name:	(2*R*,3*S*,4*R*,5*R*)-2-(2-amino-6-methoxy-purin-9-yl)-5-(hydroxymethyl)oxolane-3,4-diol
Other Names:	Nelarabine, Atriance
Physical Characteristics:	Solution for injection, Clear, colourless liquid

Description

Nelarabine is a purine analogue and is provided as a clear, colourless 5mg/ml solution for infusion. Each ml contains 5mg of nelarabine and each vial contains 250mg of nelarabine.

This medicinal product does not require any special storage conditions. Nelarabine is stable for up to 8 hours at up to 30°C once the vial is opened.

Please refer to the SmPC for more information.

Administration

Nelarabine is for intravenous use only and must only be administered under the supervision of a physician experienced in the use of cytotoxic agents. Nelarabine is not diluted prior to administration. The appropriate dose of nelarabine is transferred into polyvinylchloride (PVC) or ethyl vinyl acetate (EVA) infusion bags or glass containers and administered as a two-hour infusion in adult patients.

A dose of 1.5grams/m^2 should be given by IV on days 1, 3 & 5 following Phase 2 induction treatment.

Neurological adverse events

Severe neurological events have been reported with the use of Nelarabine. These events have included altered mental states including severe somnolence, central nervous system effects including convulsions, and peripheral neuropathy ranging from numbness and paresthesias to motor weakness and paralysis. There have also been reports of events associated with demyelination, and ascending peripheral neuropathies similar in appearance to Guillain-Barré Syndrome.

Full recovery from these events has not always occurred with cessation of Nelarabine. Therefore, close monitoring for neurological events is stongly recommended, and Nelarabine must be discontinued at the first sign of neurological events of NCI common Toxicity Grade 2 or greater.

Appendix 4: Non-IMP General Drug information

This appendix is intended as a guide for the administration of non-IMPs in the UKALL14 trial.
Those centres with a firm local policy which differs in administration detail (but not dose) from the information provided below may follow their local guidelines.

Body surface area should be calculated according to the Dubois formula:

Body Surface Area (m^2) = 0.007184 x (patient height in cm)$^{0.725}$ x (patient weight in kg)$^{0.425}$

Body surface area should be recalculated after each cycle of chemotherapy and with any major weight change.

It is recommended that sites follow local guidelines for dose adjustments for obese patients.
The site pharmacy should follow local procedures for traceability of non-IMPs.
Storage information stated below is for guidance only, and all non-IMP drugs should be stored and handled as per the SmPC for the brands used at site.

Drugs used in Induction – these may be generic or biosimilar brands as per local hospital policy.

Cyclophosphamide

Formulation	Powder for solution for injection. A white crystalline powder contained in clear glass injection vials.
Storage	Do not store above 25°C. Store in original container.<br>After reconstitution (for either intravenous or oral administration), store at 2–8°C and protect from light.
Administration	A dose of 1000mg/m^2 to be given in 250ml sodium chloride 0.9% over 30mins.<br>Give 125 mls/m^2/hour of dextrose/saline for 30 minutes before cyclophosphamide and for 3.5 hours afterwards i.e. 4 hours in total. Do not add potassium. Mesna is not needed.'

Cytarabine

Formulation	Clear, colourless solution for injection.
Storage	Do not store above 25°C. Keep container in the outer carton.
Administration	A dose of 75mg/m^2 to be given at a concentration of 20mg/ml in syringe by slow IV bolus or by IV infusion depending on local practice.

Dexamethasone

Formulation	Each tablet contains 2mg Dexamethasone PhEur.
Storage	Store below 25°C protected from light.
Administration	10mg/m^2 to be given orally in one dose with breakfast.

Daunorubicin

Formulation	Vial containing a red lyophilised powder for intravenous administration following reconstitution in water for injections and dilution with saline. Each vial contains 21.4 mg Daunorubicin hydrochloride (equivalent to 20mg as base).
Storage	Daunorubicin vials should be stored below 25°C, protected from light. After reconstitution Daunorubicin should be stored at 2–8°C, protected from light.
Administration	30mg/m^2 dose to be diluted in sodium chloride 0.9% to give final concentration of 1mg/ml and inject over 20 mins into side arm of freely running intravenous infusion of sodium chloride 0.9%. Alternatively the Daunorubicin may be added to a 100ml minibag of sodium chloride 0.9% and this solution infused over 20 minutes into the side arm of a freely running infusion of sodium chloride 0.9%. Take care to avoid extravasation.

Imatinib

Formulation	Glivec® 100mg film-coated tablets & Glivec® 400mg film-coated tablets
Storage	Do not store above 30°C. Store in the original package in order to protect from moisture.
Administration	The prescribed dose should be administered orally with a meal and a large glass of water to minimise the risk of gastrointestinal irritations. Doses of 400mg or 600mg should be administered once daily.<br><br>For patients unable to swallow the film-coated tablets, the tablets may be dispersed in a glass of mineral water or apple juice. The required number of tablets should be placed in the appropriate volume of beverage (approximately 50ml for a 100mg tablet, and 200ml for a 400mg tablet) and stirred with a spoon. The suspension should be administered immediately after complete disintegration of the tablet(s).

Methotrexate

Formulation	Methotrexate 2.5mg/ml for intrathecal use
Storage	See relevant SPC
Administration	12.5mg to be given intrathecally. Ensure national guidance on the safe administration of intrathecal chemotherapy is followed (Department of Health).

Vincristine

Formulation	A sterile, colourless solution for injection. Each 1 ml contains 1.0mg of vincristine sulphate
Storage	Store at 2 – 8°C. Keep container in the outer carton.
Administration	Ensure NPSA guidance for using Vinca Alkaloid minibags is followed (reference NPSA/2008/RRR 004.<br>When vinca alkaloids are prescribed, dispensed or administered in adult and adolescent units:<br>• Doses in syringes should no longer be used.<br>• The prescribed dose should be supplied from the hospital pharmacy ready to administer in a 50ml minibag of sodium chloride 0.9%.<br>• The following warning should be prominently displayed on the label of ALL vinca alkaloid doses 'For Intravenous Use Only – Fatal If Administered by Other Routes'.<br>• The vinca minibag should be infused intravenously over 5–10 minutes and the patient closely monitored for signs of extravasation. Incidents of extravasation should be reported and shared via the National Extravasation Information Service (www.extravasation.org.uk).

Mercaptopurine

Formulation	50mg tablets containing 50mg of Mercaptopurine
Storage	Store below 25ºC. Keep the bottle tightly closed.
Administration	60mg/m^2 orally taken once daily at the same time each day, should be administered at least 1 hour before or 3 hours after food or milk

Drugs used in Intensification

Imatinib

Formulation	Glivec® 100mg film-coated tablets & Glivec® 400mg film-coated tablets
Storage	Do not store above 30°C. Store in the original package in order to protect from moisture.
Administration	The prescribed dose should be administered orally with a meal and a large glass of water to minimise the risk of gastrointestinal irritations. Doses of 400 mg or 600 mg should be administered once daily.

	For patients unable to swallow the film-coated tablets, the tablets may be dispersed in a glass of mineral water or apple juice. The required number of tablets should be placed in the appropriate volume of beverage (approximately 50ml for a 100mg tablet, and 200ml for a 400mg tablet) and stirred with a spoon. The suspension should be administered immediately after complete disintegration of the tablet(s).

Methotrexate – HIGH DOSE

Formulation	1g or 5g vials
Storage	As per relevant SPC
Administration	3g/m^2 to be given by IV infusion (see **Appendix 15** for high dose Methotrexate guidelines)

Drugs used in Consolidation

Cyclophosphamide

Formulation	Powder for solution for injection. A white crystalline powder contained in clear glass injection vials.
Storage	Do not store above 25°C. Store in original container.<br>After reconstitution (for either intravenous or oral administration), store at 2–8°C and protect from light.
Administration	A dose of 1000mg/m^2 given at a concentration of 20mg/ml in syringe by IV bolus

Cytarabine

Formulation	Clear, colourless solution for injection.
Storage	Do not store above 25°C. Keep container in the outer carton.
Administration	A dose of 75mg/m^2 to be given at a concentration of 20mg/ml in syringe by slow IV bolus or by IV depending on local practice.

Daunorubicin

Formulation	Vial containing a red lyophilised powder for intravenous administration following reconstitution in water for injections and dilution with saline. Each vial contains 21.4 mg Daunorubicin hydrochloride (equivalent to 20mg as base).
Storage	Daunorubicin vials should be stored below 25°C, protected from light. After reconstitution Daunorubicin should be stored at 2 – 8°C, protected from light.
Administration	25mg/m^2 dose to be diluted in sodium chloride 0.9% to give final concentration of 1mg/ml and inject over 20 mins into side arm of freely running intravenous infusion of sodium chloride 0.9%. Alternatively the Daunorubicin may be added to a 100ml minibag of sodium chloride 0.9% and this solution infused over 20 minutes into the side arm of a freely running infusion of sodium chloride 0.9%. Take care to avoid extravasation.

Dexamethasone

Formulation	Each tablet contains 2.0mg Dexamethasone PhEur.
Storage	Store below 25°C protected from light.
Administration	10mg/m^2 to be given orally in one dose after breakfast.

Etoposide: HIGH DOSE

Formulation	Concentrate for solution for infusion (to dilute).
Storage	Store below 25° C, protected from light (keep vials in the outer carton). Do not freeze. Diluted solutions: Do not store the diluted product in a refrigerator (2–8 °C) as this might cause precipitation. Solutions showing any sign of precipitation should not be used.
Administration	Concentrate for solution for infusion 20 mg/ml must be diluted prior to use with either 5% dextrose in water, or 0.9 % sodium chloride solution to give a final concentration of 0.2 to 0.4mg/ml. (or as recommended by manufacturer). Give over at least 30 minutes.

Imatinib

Formulation	Glivec® 100mg film-coated tablets & Glivec® 400mg film-coated tablets
Storage	Do not store above 30°C. Store in the original package in order to protect from moisture.
Administration	The prescribed dose should be administered orally with a meal and a large glass of water to minimise the risk of gastrointestinal irritations. Doses of 400mg or 600mg should be administered once daily.<br>For patients unable to swallow the film-coated tablets, the tablets may be dispersed in a glass of mineral water or apple juice. The required number of tablets should be placed in the appropriate volume of beverage (approximately 50ml for a 100mg tablet, and 200ml for a 400mg tablet) and stirred with a spoon. The suspension should be administered immediately after complete disintegration of the tablet(s).

Methotrexate

Formulation	Methotrexate 2.5mg/ml for intrathecal use
Storage	See relevant SPC
Administration	12.5mg to be given intrathecally,ensure national guidance on the safe administration of intrathecal chemotherapy is followed (Department of Health).

Vincristine

Formulation	A sterile, colourless solution for injection. Each 1ml contains 1.0mg of vincristine sulphate
Storage	Store at 2–8°C. Keep container in the outer carton.
Administration	Ensure NPSA guidance for using Vinca Alkaloid minibags is followed (reference NPSA/2008/RRR 004).<br>When vinca alkaloids are prescribed, dispensed or administered in adult and adolescent units:<br>• Doses in syringes should no longer be used.<br>• The prescribed dose should be supplied from the hospital pharmacy ready to administer in a 50ml minibag of sodium chloride 0.9%.<br>• The following warning should be prominently displayed on the label of ALL vinca alkaloid doses 'For Intravenous Use Only – Fatal If Administered by Other Routes'.<br>• The vinca minibag should be infused intravenously over 5 – 10 minutes and the patient closely monitored for signs of extravasation. Incidents of extravasation should be reported and shared via the National Extravasation Information Service (www.extravasation.org.uk).

Mercaptopurine

Formulation	50mg tablets containing 50mg of Mercaptopurine
Storage	Store below 25ºC. Keep the bottle tightly closed.
Administration	60mg/m^2 aken once daily at the same time each day, should be administered at least 1 hour before or 3 hours after food or milk

Drugs used in Maintenance

Methotrexate oral

Formulation	2.5mg tablets
Storage	As per relevant SPC
Administration	20mg/m^2 to be given orally once a week

Methotrexate IV

Formulation	Injection
Storage	As per relevant SPC
Administration	20mg/m^2 to be given intravenously once a week (as IV bolus or infusion)

Prednisolone

Formulation	Enteric coated Tablet
Storage	As per relevant SPC
Administration	60mg/m^2 by mouth in the morning for 5 days every three months

Vincristine

Formulation	A sterile, colourless solution for injection. Each 1 ml contains 1.0 mg of vincristine sulphate
Storage	Store at 2–8°C. Keep container in the outer carton.
Administration	Ensure NPSA guidance for using Vinca Alkaloid minibags is followed (reference NPSA/2008/RRR 004).<br>When vinca alkaloids are prescribed, dispensed or administered in adult and adolescent units:<br>• Doses in syringes should no longer be used.<br>• The prescribed dose should be supplied from the hospital pharmacy ready to administer in a 50ml minibag of sodium chloride 0.9%.<br>• The following warning should be prominently displayed on the label of ALL vinca alkaloid doses 'For Intravenous Use Only – Fatal If Administered by Other Routes'.<br>The vinca minibag should be infused intravenously over 5 – 10 minutes and the patient closely monitored for signs of extravasation. Incidents of extravasation should be reported and shared via the National Extravasation Information Service (www.extravasation.org.uk).

Mercaptopurine

Formulation	50mg tablets containing 50mg of Mercaptopurine
Storage	Store below 25ºC. Keep the bottle tightly closed.
Administration	75 mg/m^2 orally aken once daily at the same time each day, should be administered at least 1 hour before or 3 hours after food or milk.

Drugs used in transplant conditioning regimens

Alemtuzumab

Formulation	MabCampath 30mg/ml concentrate for solution for infusion
Storage	Store in a refrigerator (2–8°C).<br>Do not freeze.<br>Store in the original package in order to protect from light.<br>Alemtuzumab should be used within 8 hours after dilution. Solutions may be stored at 15°C-30°C or refrigerated. This can only be accepted if preparation of the solution takes place under strictly aseptic conditions and the solution is protected from light
Administration	The required amount of the vial contents should be added to 100 ml of sodium chloride 9mg/ml (0.9%) solution for infusion or glucose (5%) solution for infusion. The bag should be inverted gently to mix the solution. Care should be taken to ensure the sterility of the prepared solution particularly as it contains no antimicrobial preservatives.<br><br>All doses should be administered by intravenous infusion over approximately 2 hours.<br><br>Patients should be premedicated with oral or intravenous steroids, an appropriate antihistamine and analgesic 30-60 minutes prior to each alemtuzumab infusion

Cyclophosphamide: HIGH DOSE

Formulation	Powder for solution for injection. A white crystalline powder contained in clear glass injection vials.
Storage	Do not store above 25°C. Store in original container.<br>After reconstitution (for either intravenous or oral administration), store at 2–8°C and protect from light.
Administration	A dose of 60mg/kg to be given, as 20mg/ml cyclophosphamide in sodium chloride 0.9% in empty ethyl vinyl acetate (EVA) bag over 2 hours

Etoposide:

Formulation	Concentrate for solution for infusion (100mg in 5ml vials)
Storage	Room temperature
Administration	60mg/kg To be given undiluted via central venous catheter over approx 4 hours. Use polyethylene or polyethylene lined line – new line for each syringe, primed with saline. Do not flush line, disconnect at source and use new line for each syringe. An extra 2ml Etoposide is supplied in each syringe to account for Etoposide lost in line.<br><br>Ensure anti-emetics are prescribed. Ensure patient remains well hydrated.<br><br>Patient must have a baseline ECG before starting the infusion and be coupled to a cardiac monitor throughout. Regular recordings of pulse and blood pressure should be made throughout the infusion, which can be slowed if necessary. Maintenance of blood pressure using fluid support or colloids may be necessary

Fludarabine

Formulation	Powder for solution for injection or infusion
Storage	See relevant SPC
Administration	Fludarabine should be prepared for parenteral use by aseptically adding sterile water for injection. When reconstituted with 2 ml of sterile water for injection, the powder should fully dissolve in 15 seconds or less. Each ml of the resulting solution will contain 25 mg of fludarabine phosphate, 25 mg of mannitol, and sodium hydroxide (to adjust the pH to 7.7). The pH range for the final product is 7.2 – 8.2.<br><br>Dilution<br>The required dose (calculated on the basis of the patient's body surface) is drawn up into a syringe. For intravenous bolus injection this dose is further diluted in 10 ml sodium chloride 9mg/ml (0.9%). Alternatively, for infusion, the required dose may be diluted in 100 ml sodium chloride 9mg/ml (0.9%) and infused over approximately 30 minutes.

Melphalan

Formulation	Freeze-dried powder for injection.
Storage	Store below 30° C, Protect from light & Do not refrigerate.
Administration	For intravenous administration, Melphalan Injection solution may be administered diluted in an infusion bag. Melphalan is not compatible with infusion solutions containing dextrose and it is recommended that only sodium chloride intravenous infusion 0.9% w/v is used. When further diluted in an infusion solution, Melphalan has reduced stability and the rate of degradation increases rapidly with rise in temperature. If Melphalan is infused at a room temperature of approximately 25°C, the total time from preparation of the injection solution to the completion of infusion should not exceed 1.5 hours. Should any visible turbidity or crystallisation appear in the reconstituted or diluted solutions, the preparation must be discarded. Hydration and forced diuresis is recommended but not mandatory- please follow your local practice.

Please check relevant Summary Product Characteristics for brand of drug used, for storage, special precautions and contraindications, interactions, undesirable effects and stability information.

Appendix 5: Dose modifications for toxicity

Steroids

Hypertension: Steroid should not be reduced. Sodium restriction and anti-hypertensives should be employed in an effort to control hypertension.

Malignant Hypertension: Reduce dose 33%. Sodium restriction and anti-hypertensive drugs may also be utilised.

Hyperglycemia: Steroids should not be reduced if the patient develops clinical signs of diabetes. Rather, insulin therapy should be employed to control the blood glucose level such that symptoms and signs are minimal.

Pancreatitis: Do not modify dose.

Psychosis: Administer half dosage of steroid.

Suspected steroid-induced myopathy: Measure CPK with isoenzymes, consider EMG studies.

Avascular necrosis: Contact CI or clinical coordinators if AVN develops before Maintenance therapy has begun. Omit further steroids if AVN develops during maintenance.

Varicella Zoster: Steroids should be held during active infection except during Induction (Discuss with co-ordinators). They should not be held during incubation period following exposure to Varicella,.

Severe Dexamethasone intolerance: change to Prednisolone 40mg/m^2.

Vincristine

Seizures: Hold 1 dose, then reinstitute.

Severe foot drop, paresis or ileus: Hold dose(s); institute aggressive regimen to treat constipation (except enemas if neutropenic), if present. When symptoms abate, resume at 1.0 mg/m^2; escalate to full dose as tolerated.

Jaw pain: Treat with analgesics; do not modify Vincristine dose.

Hyperbilirubinemia: Withhold if total bilirubin >50. Administer 50% of dose if total bilirubin 25–50. Do not alter dose for abnormal transaminases.

Asparaginase

Anaphylaxis or anaphylactoid reactions: Pegylated asparaginase should be discontinued if the patient develops a Grade 2–4 toxicity. Send blood samples to the Adult ALL MRD laboratory for asparaginase antibodies and change to Erwinase (appendix 14).

Symptomatic pancreatitis: Discontinue L-asparaginase in the presence of symptomatic pancreatitis documented by an elevated serum amylase or lipase level or ultrasonographic abnormalities. Do not give further doses if there is a prior history of asparaginase induced pancreatitis.

Hyperglycaemia: Do not modify dose. Administer Insulin as required.

Ketoacidosis: Hold L-Asparaginase until blood glucose can be regulated with insulin.

Coagulopathy: When significant symptomatic coagulopathy occurs, withhold L-asparaginase until resolved. Routine clotting screens are not recommended. Coagulopathy without bleeding is not an indication to withhold L-asparaginase.

Liver Dysfunction: Check LFTs only if patient jaundiced. Withhold if total bilirubin > 50. Do not alter dose for abnormal transaminases.

Anthracyclines (Daunorubicin)

Hyperbilirubinemia: If total bilirubin >120 omit dose; if >90 but <120 give 25% of dose. If >50 but <90 give 50% of dose, and if <50 give full dose. Check LFTs only if patient jaundiced. Do not alter dose for abnormal transaminases.

Intrathecal Methotrexate

Any significant neurotoxicity not due to lumbar puncture syndrome (low opening pressure, slow CSF flow, orthostatic symptoms) should be reported.

Systemic toxicity: The dosage for Intrathecal Methotrexate will not be reduced for systemic toxicity (myelosuppression, mucositis, etc).

Viral, bacterial or fungal meningitis: Omit until resolved.

Encephalopathy attributed to intrathecal Methotrexate: see section 7.2.1.

Oral Methotrexate

Mucositis: For grade 2 mucositis of over 3 days duration, decrease MTX dose by 30%. For grade 3-4, mucositis, withhold MTX until resolved; resume at 50% of the previously attained dose and subsequently escalate to 75% to 100% dose at 10 day intervals provided grade 3-4 toxicity does not recur. Consider culturing lesions for herpes simplex if mucositis persists or recurs.

Liver: Check LFT's only if patient jaundiced. If bilirubin is >50 micromoles/L omit MTX until it is less than 20 micromoles/L, and then restart at half of the previously attained dose. Escalate from 50% to 75% to 100% dose at 10-day intervals provided hyperbilirubinaemia does not recur. Do not modify dosage for elevated aminotransferases.

Kidney (Grade 3-4): Omit MTX until grade 0 toxicity (ie completely resolved). Resume at 100% of the previously attained dose and continue at 10-day intervals.

Intravenous Methotrexate

Please see Appendix 15.

Cyclophosphamide

Prior history of gross haematuria or microscopic haematuria: Hydrate at 125 ml/m^2/hr for 24 hours after dose and use Mesna 360 mg/m^2 pre, and 4, 7, 11 hours post dose.

Acute Fluid retention: Treat with Frusemide and saline; do not modify dose.

Cytarabine

Hyperbilirubinaemia: if total bilirubin >120 omit dose; if >90 but <120 give 25% of dose. If >50 but <90 give 50% of dose, and if <50 give full dose. Check LFT's only if patient jaundiced. Do not alter dose for abnormal transaminases.

Mercaptopurine

Hyperbilirubinaemia: If bilirubin >50micromol/l omit mercaptopurine until it is less than 20micromol/l and then restart at helf the previously attained dose. Escalate from 50% to 75% to 100% dose at 10-day intervals provided hyperbilirubinaemia does not recur. Do not modify dosage for elevated aminotranseferases.

Nelarabine

Nelarabine must be discontinued at the first sign of neurological events of National Cancer Institute Common Terminology Criteria Adverse Event (NCI CTCAE) grade 2 or greater. Delaying subsequent dosing is an option for other toxicities, including haematological toxicity.

Renal Impairment

Nelarabine has not been studied in individuals with renal impairment. Nelarabine and 9-β-D-arabinofuranosylguanine (ara-G) are partially renally excreted. There are insufficient data to support a dose adjustment recommendation for patients with a renal clearance of creatinine CrCl less than 50 ml/min. Patients with renal impairment must be closely monitored for toxicities when treated with nelarabine.

Hepatic Impairment

Nelarabine has not been studied in patients with hepatic impairment. These patients should be treated with caution.

Patients receiving nelarabine are recommended to receive intravenous hydration according to standard medical practice for the management of hyperuricemia in patients at risk of tumour lysis syndrome. For patients at risk of hyperuricemia, the use of allopurinol should be considered.

Appendix 6: Assessment of GVHD

Graft Versus Host Disease (GVHD) arises due to reactivity of cytotoxic T lymphocytes against recipient cells, through both HLA- and minor histo-incompatibility between the donor and the recipient. After the transplant the reaction usually requires 10 or more days for priming and proliferation of T-cells to occur.

GVHD is divided into two forms, each of which tend to produce distinct clinical syndromes:

- Acute GVHD: <100 days post-transplant (grades I to IV)
- Chronic GVHD: >100 days post-transplant (limited or extensive)

Organ involvement for GVHD should be staged using the criteria outlined in the tables below. Biopsy of each organ site at diagnosis or major change in disease activity will be performed unless clinical circumstances make it impossible.

Acute GVHD

Acute graft versus host disease predominantly affects three organs, either singly or together:

- Skin - maculopapular rash, erythema, desquamation
- Gastrointestinal tract - nausea, vomiting, diarrhoea
- Liver - raised alkaline phosphatase, bilirubin, later hepatitis

Glucksberg criteria for assessment of Acute GVHD: Table A

Stage	Skin	Liver	Gut
1	rash <25% body	bilirubin 35 – 50 uM/l	Diarrhoea <1 l/day
2	rash 25-50% body	bilirubin 51-100 uM/l	Diarrhoea 1-1.5 l/day
3	rash >50% body	bilirubin 101-250 uM/l	Diarrhoea >1.5 l/day
4	desquamation or bullae	bilirubin >250 uM/l	Pain or ileus

Glucksberg criteria for assessment of Acute GVHD: Table B

Grade	Skin stage	Liver stage	Gut stage
I	1-2	0	0
II	1-3	1	1
III	2-3	2-3	2-3
IV	2-4	2-4	2-4

Chronic GVHD

Classification scheme for chronic GVHD (Shulman *et al*):

**Limited**<br>Either or both:<br>1. Localised skin involvement<br>2. Hepatic dysfunction due to chronic GVHD
**Extensive**<br>Either:<br>1. Generalised skin involvement; or<br>2. Localised skin involvement and/or hepatic dysfunction due to chronic GVHD, plus:<br>a. Liver histology showing chronic aggressive hepatitis, bridging necrosis or cirrhosis; or<br>b. Involvement of eye: Schirmer's test with less than 5mm wetting; or<br>c. Involvement of minor salivary glands or oral mucosa demonstrated on labial biopsy; or<br>d. Involvement of any other target organ

Appendix 7: Cytogenetic Definitions and Detection Strategy

Definition and detection of high risk abnormalities

It is essential that conventional G-banded cytogenetics analysis is performed on all trial patients. In addition, FISH or RT-PCR to detect high risk cytogenetic abnormalities will be necessary in all patients with a normal karyotype or failed cytogenetics.

The four cytogenetic subgroups which will be treated as high risk in UKALL14 are:

- Philadelphia chromosome / t(9;22)(q34;q11)/*BCR-ABL1*
- t(4;11)(q21;q23) / *MLL-AFF1*
    - NB *AFF1* was previously known as *AF4* and *MLLT2*
- low hypodiploidy / near-triploidy (Ho-Tr)
- complex karyotype (CK).

Philadelphia chromosome / t(9;22)(q34;q11)/*BCR-ABL1*

Patients with Philadelphia positive ALL will receive imatinib in induction in UKALL14 and therefore all patients must be identified as quickly as possible. All patients must be screened by FISH using a dual-colour dual-fusion translocation probe or RT-PCR using standard primers.

T(4;11)(q21;q23) / *MLL-AF4*:

This translocation is readily detectable by conventional cytogenetics but patients with a normal karyotype or failed cytogenetics must be screened using either RT-PCR using standard primers or the following FISH strategy:

- FISH with a *MLL* dual colour break apart probe.
- Patients with a *MLL* split signal pattern should be screened using an appropriate *AF4* dual colour break apart probe, unless a t(4;11) is visible cytogenetically. Currently, no *AF4* probe is commercially available. Thus we advise the use of a home-grown dual-colour probe comprising the tiling path clones RP11-397E7 and RP11-476C8 which cover the 5′ and 3′ portions of the gene respectively. For further advice please contact the LRCG (see below). If necessary the LRCG will undertake this specific FISH test centrally.

Low hypodiploidy (30-39 chromosomes) / near-triploidy (60-78 chromosomes) (Ho-Tr):

This cytogenetic subgroup comprises two related entities a low hypodiploid clone and its "doubled" near-triploid counterpart[68]. Although both entities are usually detectable by conventional cytogenetics occasionally just one is visible. In cases where only the near-triploidy clone is visible there may be some confusion with high hyperdiploidy if the karyotype has 60-65 chromosomes. Distinction between the two subgroups can be made on the basis of chromosomes gained and the proportion of tetrasomic chromosomes. In particular, near-triploidy karyotypes in this subgroup are usually tetrasomic for chromosomes 1, 6, 11 and 18, and are almost always disomic for chromosomes 3, 7 and 15. If there is any doubt in classifying patients please contact the LRCG (see contact details below).

Patients with a normal karyotype or failed cytogenetics should be screened for Ho-Tr using one of the following techniques:

- Flow cytometry to determine the DNA content of the blast cells – cases with Ho-Tr typically display two aneuploid peaks – low hypodiploid (0.7-0.9) and near-triploid (1.4-1.6).
- FISH using the Chromoprobe Multiprobe-I System (CytoCell, www.cytocell.com) or equivalent, which allows the simultaneous enumeration of all chromosomes on a single slide.
- FISH using selected centromeric probes. Defining precise chromosomes to test is difficult as the key feature of this subgroup is the modal chromosome number of the karyotype rather than the gain or loss of any individual chromosome or sets of chromosomes. However, characteristic chromosomes include 1, 6, 11 and 18 which are usually disomic and tetrasomic and 3, 7 and 15 which are usually monosomic and disomic in the low hypodiploid and near-triploid clones respectively.

Complex karyotype (CK):

The definition of a complex karyotype is five or more chromosomal abnormalities in the absence of an established translocation (e.g. t(1;19)(q23;p13), t(11;19)(q23;p13), t(12;21)(p13;q22)/*ETV6-RUNX1* etc.) or ploidy subgroup (e.g. low hypodiploidy / near–triploidy, high hyperdiploidy, tetraploidy). Detection is by conventional cytogenetics only. If there is any doubt in classifying patients please contact the LRCG (see contact details below).

Contact details for Leukaemia Research Cytogenetics Group (LRCG)

Professor Anthony V Moorman, 0191 282 1323, anthony.moorman@ncl.ac.uk
Professor Christine J Harrison, 0191 282 1320, christine.harrison@ncl.ac.uk
Ms Claire Schwab, 0191 282 1324, claire.schwab@ncl.ac.uk

Northern Institute for Cancer Research, Level 5, Sir James Spence Institute,
Royal Victoria Infirmary, Queen Victoria Road, Newcastle upon Tyne, NE1 4LP
tel: 0191 282 1324 | fax: 0191 282 1326

Appendix 8: Central laboratories and trial schedule

The UCL Cancer Institute MRD laboratory will quantify MRD by immunoglobulin/T-cell receptor gene re-arrangements for Ph- ALL and by BCR-Abl quantification for Ph+ ALL.

The UCL Cancer Institute MRD Lab will be required to charge for MRD Analysis. This will be charged as per the national rate.

Request forms

These will be provided by our laboratory. They have been designed to provide sufficient information for each patient to be reliably identified and for MRD results to be correctly interpreted for reporting. It is important that you let us know the blast percentage in the marrow as we need this to interpret the result. It is important to let us know as much immunphenotyping information as you have available, since it assists us to set up the correct MRD marker panel. Please send a Sample Request Form with all samples that are sent to the UCL Cancer Institute MRD Lab for MRD Anaylsis and Correlative Science Testing.

MRD sample Collection and Transport

<u>At diagnosis (randomised and 'registration only' patients)</u>

3 – 5mls of bone marrow from all adults with suspected leukaemia should be placed into EDTA. When bone marrow is not available, 30 -50 mls of blood collected into EDTA is also acceptable at diagnosis. Please note that peripheral blood is of no value as an MRD sample at other time points.

The final date for baseline samples to be received at the MRD laboratory for the trial is 29th October 2021.

<u>End of phase 1 therapy (randomised and 'registration only' patients)</u>

5ml bone marrow aspirate in EDTA following Phase 1 therapy (this is for the secondary end-point of the antibody randomisations and will not be reported)

<u>End of phase 2 therapy (randomised and 'registration only' patients)</u>

5ml bone marrow aspirate in EDTA following Phase 2 induction therapy (this is for the risk stratification and will be reported within 10 working days of specimen receipt to sites. <u>The report will be sent to the Randomising Clinician and a copy sent to UCL CTC who will confirm the patient's risk status.</u>)

If the MRD result is not available (failed or specimen not sent) patients should be considered standard risk in absence of any other high risk features.

<u>Post-transplant (non-myeloablative transplant, randomised patients only)</u>

3-5ml bone marrow aspirate in EDTA to be sent at 3 monthly intervals from 3 months post-transplant until 2 years post-transplant or relapse.

<u>At relapse</u> <u>(randomised and 'registration only' patients)</u>

We hope that relapse will be infrequent, but if it does occur, we would be pleased to receive a specimen (3-5ml bone marrow aspirate in EDTA, or peripheral blood 30-50ml in EDTA if WCC > 30x 10^9/l) so that we can

identify whether or not the relapse has originated, or not, from the Ig-TCR confirmed clone which we have been using as an MRD marker.

Transport

All samples should be packaged appropriately using local packaging, including a sample request form and should be sent by courier or by 1st class post to arrive the same day or overnight to the following address:

Minimal Residual Disease Laboratory
URGENT UKALL14 STUDY SAMPLE: (FAO Adele Fielding or Krisztina Alapi)
UCL Cancer Institute
Paul O'Gorman Building
72 Huntley Street
London UK
WC1E 6DD

MRD Laboratory email: ALLMRDlab@ucl.ac.uk

The package should be clearly marked on the outside as "URGENT, UKALL14 STUDY SAMPLE"

Weekend and Bank Holiday Shipments

For samples taken on a Friday, please store in a fridge until Monday and send via courier or 1st class post. For samples taken before a Bank Holiday weekend, please store the sample in a fridge until the next working day and send via courier or 1st class post.

Sample processing

On receipt of bone marrow aspirates, the MRD laboratory will assign the patient and the sample a unique number according to the standard operating procedure. Cell counts will be recorded and DNA extracted within 48 hours of receipt of the sample. A minimum of 10 micrograms of DNA is required at diagnosis and 5 micrograms for follow-up samples.

UK MRD Network of Laboratories

MRD monitoring for this study will be undertaken at UCL Cancer Institute headed by Dr Adele Fielding. This laboratory has recently joined the UK network of MRD laboratories. This network was conceived for the purpose of MRD monitoring for the current UK childhood trial (UKALL 2003) as such it has demonstrated a robust framework for providing quality assured results.

The UK MRD network acts as a virtual single laboratory using a standard operating procedure and centrally distributed reagents to measure MRD by the Real time quantitative (RQ PCR) Allele Specific Oligonucleotide (ASO) PCR method. This technique is considered to be the most widely applicable and sensitive approach to MRD detection in ALL. The network is co-ordinated by clinical and scientific leads based at Bristol Children's Hospital (Dr J Hancock to co-ordinate) and headed by a Steering committee chaired by Professor Nicholas Cross. It participates in national and European External Quality Assurance schemes under Professor J Van Dongen's direction.

Funding

The UCL Cancer Institute MRD Laboratory will be required to charge participating centres for MRD testing as it has been deemed by CR-UK as an 'excess NHS treatment cost'.

Appendix 9: Donor Peripheral Blood Stem Cell Collection & Return

The donor will be given G-CSF 10 micrograms/kg/day s.c. from day -4 to day 0. Mononuclear cells will be collected by leucapheresis on days 0 and +1. If sufficient cells are harvested on day 0 no further collection is required. The leucapheresis product will be assessed for nucleated cell count, CD34 content and NK and T-cell subset content.
NB: It is mandatory to assess the leucapheresis product for nucleated cell cout and CD34. Evaluation of other cell subset content (NK cells, T-cells) is desirable but it is at the centre's discretion.

A minimum dose of $2x10^6$ CD34+ cells/kg will be returned to the patient on Day 0 of the transplant. The target dose for returned cells is > 4 $x10^6$ CD34+ cells/kg.
G-CSF 5 micrograms/kg s.c. (or 300 micrograms Filgrastim) will be given to the patient from day +6 until neutrophils $>1x10^9$/l on 2 consecutive days.

Chugai Pharma UK will provide a 25% retrospective reimbursement in stock for rHuG-CSF (Lenograstim) used within the study period.

Please contact **medicalaffairs@chugai-pharm.co.uk** with regards to making a claim.

Granocyte is available at NHS contract prices through AAH Hospital Service Telephone 0845 607 6699.

Please refer to the SpC for Lenograstim: www.medicines.org.uk

Appendix 10: Haematopoietic Cell Transplantation-Specific Comorbidity Index

Comorbidity index = sum of scores defined in table below

**Comorbidities**	**Definitions**	**Score**
Arrhythmia	Atrial fibrillation or flutter, sick sinus syndrome, or ventricular arrhythmias	1
Cardiac	Coronary artery disease*, congestive heart failure, myocardial infarction, or EF≤50%	1
Inflammatory bowel disease	Crohn's disease or ulcerative colitis	1
Diabetes	Requiring treatment with insulin or oral hypoglycaemic, but not controlled with diet alone	1
Cerebrovascular disease	Transient ischaemic attacks or cerebrovascular accident	1
Psychiatric disturbance	Depression/anxiety requiring psychiatric consult and/or treatment at the time of transplant	1
Hepatic (mild)	Chronic hepatitis, bilirubin>ULN to 1.5xULN, or AST/ALT>ULN to 2.5xULN	
Obesity	BMI>35 for adults or with BMI-for-age percentile of ≥95th percentile for children	1
Infection	Documented infection or fever of unknown aetiology requiring anti-microbial treatment before, during and after the start of conditioning regimen	1
Rheumatological	SLE, RA, polymyositis, mixed CTD and polymyalgia rheumatic	2
Peptic ulcer	Requiring treatment	2
Renal (moderate/severe)	Serum creatinine>2mg/dL†, on dialysis or prior to renal transplantation	2
Pulmonary (moderate)	DLCO and/or FEV_1 66-80% or dyspnoea on slight activity	2
Prior solid tumour	Treated at any point in the patients history, excluding non-melanoma skin cancer	3
Heart valve disease	Except asymptomatic mitral valve prolapse	3
Pulmonary (severe)	DLCO and/or FEV_1 ≤65% or dyspnoea at rest or requiring oxygen	3
Hepatic (moderate/severe)	Liver cirrhosis, bilirubin>1.5xULN, or AST/ALT> 2.5xULN	3

EF – ejection fraction; ULN – upper limit of normal; AST – aspartate aminotransferase; ALT – alanine aminotransferase; BMI – body mass index; SLE – systemic lupus erythematosus; RA – rheumatoid arthritis; CTD – connective tissue disease; DLCO – diffusion capacity of carbon monoxide; FEV_1 – forced expiratory volume in 1 second.

* one or more vessel coronary artery stenoses requiring medical treatment, stent or bypass graft.

† to convert creatinine from mg/dL to µmol/L, multiply by 88.4

Appendix 11: ECOG performance status

Score	Definition
0	Asymptomatic and fully active
1	Symptomatic; fully ambulatory; restricted physically strenuous activity
2	Symptomatic; ambulatory; capable of self-care; more than 50 percent of waking hours are spent out of bed
3	Symptomatic; capable of limited self care; spends more than 50 percent of time in bed but not bedridden.
4	Completely disabled; no self-care; bedridden

Appendix 12: Schedule of Assessments (including testing for MRD & Correlative Science)

Table 12.1a: Treatment & Follow up (randomised chemotherapy patients)

See table 12.1.b for assessments in patients who undergo transplant

Timing / Assessment	Registration	During Phase 1 treatment (timing dependent on age[8])	Upon recovery from Phase 1 induction	Upon recovery from Phase 2 induction	During intensification (d2 and d16[9])	After intensification	After Cycle 1 consolidation	After Cycle 2 consolidation	Before Cycle 3 consolidation d29	After Cycle 3 consolidation	After cycle 4 consolidation	During maintenance therapy (every 3 months)	Annual Follow up (until patient death)	2 year follow up appointment	At Relapse
Informed consent	X														
Full medical history & physical examination	X												X	X	
Height, Weight, & BSA	X														
ECOG Status	X														
FBC	X		X	X		X	X	X	X	X	X	X	X	X	
Biochemistry	X														
Bone marrow aspirate & trephine (**SENT TO MRD LAB @ UCL CANCER INSTITUTE**)	X[1]		X[2]	X[2, 10]											X[7]
Pregnancy test	X														
Confirmation of disease diagnosis	X														
Cytogenetics	X[3]														
Lumbar puncture	X[4]														
Peripheral blood sample- for asparaginase activity (**SENT TO MRD LAB @UCL CANCER INSTITUTE**)		X[5, 8]			X[5, 9]										
Plasma collected – for asparaginase coagulation activity **Stored locally at -80°C (or if not possible, -20°C)**		X[6, 8]			X[6, 9]										
Assessment of disease status													X	X	
Echocardiogram														X	
General Health Questionnaire (GHQ-12)														X	
Late Effects of Treatment Assessment														X	
Record of AVN or serious cardiac problems													X	X	

1= Bone marrow 3-5ml in EDTA (peripheral blood 30-50ml in EDTA if bone marrow unavailable). (**Please see protocol section 8.2.1 & Appendix 8)**
2= Bone marrow aspirate: 3-5ml in EDTA for MRD assessment. (**Please see protocol section 8.2.1 & Appendix 8**)
3= Cytogenetics/molecular assessment of BCR-ABL and MLL on bone marrow. A copy of the results should be sent to the Leukemia Research Cytogenetics Group. (**Please see Appendix 7**)
4= Lumbar puncture is not required at diagnosis except in the case of suspected central nervous system involvement. (**Please see protocol section 5.1**)
5= 5ml peripheral blood in a serum tube (for Asparaginase activity assay and anti-Asparaginase antibodies). (**Please see protocol section 8.2.1 & Appendix 8**)
6= 4.5ml sample in a sodium citrate tube (filled to the line) **(Please see protocol section 8.2.1)**
7 = Bone marrow 3-5ml in EDTA (peripheral blood 30-50ml in EDTA if WCC >$30x10^9$/l). (**Please see protocol section 8.2.1 & Appendix 8)**
8 = Timing of phase 1 samples as follows: Patient aged ≤41: asparaginase levels to be taken on days 3 or 4 & 18; plasma sample to be taken on days 3 or 4
Patients aged ≥41: asparaginase levels to be taken on days 18 & 32; plasma sample to be taken on day 18
9 = Timing of intensification samples as follows: asparaginase levels to be taken on days 2 & 16; plasma sample to be taken on day 2
10 = For T2 patients, end of phase 2 bone marrow should be taken **before** starting nelarabine

Table 12.1b Transplant & Follow up (for randomised transplant patients)

Transplant patients should follow the schedule of assessments on the previous page for Phase I & II treatment, and for intensification, if applicable.

**Timing** / **Assessment**	Pre-Transplant	100 days Assessment	3 monthly assessments (for 2 years following transplant)	Annual Follow up (until patient death)	2 year follow up appointment	Relapse
Full medical history & physical examination	X					
ECOG Status & Karnofsky Performance Status	X	X	X	X	X	
Co-morbidity Index	X					
Full blood count with differential	X			X	X	
Biochemistry (including LDH) & assessment of liver and renal function	X					
Pregnancy test, Microbial Titres, Urinalysis & clotting screen (including PT/APTT)	X					
Bone marrow (aspirate & trephine) (**SENT TO MRD LAB @UCL CANCER INSTITUTE)**			X[1]			X[4]
Assessment of cardiac & pulmonary function	X					
Peripheral blood sample or buccal swab - mini satellite regions (patient and donor; send to local chimerism lab)	X[2]					
Chimerism studies –Peripheral blood sample (patient only; send to local chimerism lab, anonymised report to UCL CTC)		X[3]	X[3]			X[2]
GVHD Assessment		X	X			
Donor Lymphocyte Infusion			If MRD+ or mixed chimerism			
Clinical examination (as necessary)				X	X	
Assessment of disease status		X	X	X	X	
Employment status			X	X	X	
Record of AVN or serious cardiac problems				X		
Echocardiogram					X	
General Health Questionnaire (GHQ-12)					X	
Late Effects of Treatment Assessment					X	

1= 3-5ml bone marrow aspirate in EDTA for MRD assessment. FOR NON-MYELOABLATIVE TRANSPLANT PATIENTS ONLY.
2= Peripheral blood or buccal swab from donor and recipient for assessment of mini-satellite regions, as per local practice. FOR NON-MYELOABLATIVE TRANSPLANT PATIENTS ONLY. NB anonymised local chimerism report must be sent to UCL CTC prompty
3 = Peripheral blood for chimerism, as per local practice. FOR NON MYELOABLATIVE TRANSPLANT PATIENTS ONLY. NB anonymised local chimerism report must be sent to UCL CTC prompty
4 = Bone marrow 3-5ml in EDTA (or peripheral blood 30-50ml in EDTA if WCC >30x109/l) (**Please see protocol section 8.2.1 & appendix 8)**

Appendix 13: General Health Questionnaire (GHQ-12)

General Health Questionnaire

Name...

We want to know how your health has been in general over the last few weeks. Please read the questions below and each of the four possible answers. Circle the response that best applies to you. Thank you for answering all the questions.

Have you recently:

1. been able to concentrate on what you're doing?

Better than usual	same as usual	less than usual	much less than usual
(0)	(1)	(2)	(3)

2. lost much sleep over worry?

Not at all   no more than usual   rather more than usual   much more than usual

3. felt that you are playing a useful part in things?

More so than usual   same as usual   less so than usual   much less than usual

4. felt capable of making decisions about things?

More so than usual   same as usual   less than usual   much less than usual

5. felt constantly under strain?

Not at all   no more than usual   rather more than usual   much more than usual

6. felt you couldn't overcome your difficulties?

Not at all   no more than usual   rather more than usual   much more than usual

7. been able to enjoy your normal day to day activities?

More so than usual same as usual less so than usual much less than usual

8. been able to face up to your problems?

More so than usual same as usual less than usual much less than usual

9. been feeling unhappy or depressed?

Not at all no more than usual rather more than usual much more than usual

10. been losing confidence in yourself?

Not at all no more than usual rather more than usual much more than usual

11. been thinking of yourself as a worthless person?

Not at all no more than usual rather more than usual much more than usual

12. been feeling reasonably happy, all things considered?

More so than usual same as usual less so than usual much less than usual

General Health Questionnaire Scoring

Scoring – Likert Scale 0, 1, 2, 3 from left to right.

12 items, 0 to 3 each item

Score range 0 to 36.

Scores vary by study population. Scores about 11-12 typical.

Score >15 evidence of distress

Score >20 suggests severe problems and psychological distress

Appendix 14: Guidance on Use of Erwinia Asparaginase (Erwinase®) in patients with systemic reactions to Pegylated-Asparaginase.

1. A licensed preparation of Erwinia Asparaginase (Erwinase®) is now available, thus providing an effective alternative for patients with hypersensitivity to E.Coli Asparaginase.
2. Erwinase®.will be marketed and distributed by EUSA Pharma
3. Erwinase® should be used in place of Pegylated E. Coli Asparaginase in the following circumstances:
   - Systemic hypersensitivity reactions to n Pegylated E.Coli Asparaginase (Oncaspar). This includes patients with generalised rash with or without anaphylactic symptoms, but not those with only local pain or redness at the site of injection.
   - Patients with previously documented systemic reactions to Pegylated E.Coli Asparaginase should receive Erwinase® in any remaining Asparaginase containing courses.
4. Each dose of Pegylated Asparaginase (Oncaspar) should be replaced with 6 doses of 20,000 Units/m^2 Erwinase® given on Mondays, Wednesdays and Fridays. (*or this could be 6 doses q 48 hours if preferred)*
5. Erwinase® should be administered by intra-muscular injection. The individual dose may be split between two injection sites if injection volume more than 4ml.
6. Please notify the trials office of patients switching to Erwinase®.

Chemical name – *Erwinia* L-asparaginase
Other names – ERWINASE®, Crisantaspase (Asparaginase from *Erwinia chrysanthemi*; *Erwinia* L-asparaginase)
Formulation - 10,000 Units/vial, Lyophilisate for solution for injection, White lyophilised powder in a vial.
Special warnings and precautions for use
Warnings: Anaphylactic reactions have been observed after the use of Erwinase. Facilities should be made available for management of an anaphylactic reaction, should it occur, during administration. Careful observation is required on re-exposure to L-asparaginase after any time interval, which may increase the risk of anaphylactic reactions occurring.
Careful monitoring before and during therapy is necessary:
• Serum amylase, lipase and/or insulin levels should be monitored to exclude hyperglycaemia and severe pancreatitis. Hyperglycaemia may be treated with insulin, if needed.
• Routine clotting screening may be performed before treatment initiation. If significant symptomatic coagulopathy occurs withhold L-asparaginase treatment until resolved then continue according to protocol.

• Hepatic function tests should be monitored regularly during therapy.

Storage *– Store at 2-8°C*

Administration – The contents of each vial should be reconstituted in 1 ml to 2 ml of sodium chloride (0.9%) solution for injection. Slowly add the reconstitution solution against the inner vial wall, do not squirt directly onto or into the powder. Allow the contents to dissolve by gentle mixing or swirling maintaining the vial in an upright position. Avoid froth formation due to excessive or vigorous shaking.

The solution should be clear without any visible particles. Fine crystalline or thread-like wisps of protein aggregates may be visible if shaking is excessive. If there are any visible particles or protein aggregates present the reconstituted solution should be rejected.

The solution should be administered within 15 minutes of reconstitution. If a delay of more than 15 minutes between reconstitution and administration is unavoidable, the solution should be withdrawn into a glass or polypropylene syringe for the period of the delay. The solution should be used within 8 hours.

Give by intra-muscular injection, if the volume is over 4ml the individual dose may be split between two injection sites.

See also SPC at www.medicines.org.uk

Appendix 15: Guideline for the administration of IV High-Dose Methotrexate

Regimen for administration of high-dose methotrexate

NOTE: The guidance for administration of high dose methotrexate is a detailed suggestion for trial participants to follow. It contains all the necessary information to give the drug safely. However, those centres who have a firm local policy in place which differs in administration detail (but not dose) from the suggestions put forward here can administer high dose methotrexate within UKALL14 according to their local policies.

One week before admission for the 1st methotrexate infusion the Creatine Clearance (measured according to local practice) should be determined. The initial Creatinine Clearance before starting methotrexate should ideally be >100 ml/minute.

Patients with a clearance between 80-100 ml/minute before the first dose of High Dose Methotrexate MUST have a measured Creatinine clearance (24 urine collection) BEFORE the second dose and dose adjustments (as above) made if the result is less than 80 ml/minute.

Also repeat Creatinine Clearance before the 2nd infusion if there is delayed methotrexate excretion after the first course.

Based on a dose of 3g/m^2 of Methotrexate and renal function pre-treatment use Dose reductions as follows:

Pre Cycle 1

CrCl (ml/min)	Dose
> 80 mls/min	100%
50-80 mls/min	50 %
<50 mls/min	0 %

Pre Cycle 2

CrCl (ml/min)	Dose
> 50 mls/min	100%
<50 mls/min	0 %

Consult the TMG if in any doubt regarding the high dose methotrexate.

METICULOUS ATTENTION SHOULD BE PAID AT ALL TIMES TO CHANGES IN CREATININE CLEARANCE DURING THE HIGH DOSE METHOTREXATE PHASE. (BOTH WITHIN AND BETWEEN EACH COURSE OF METHOTREXATE).

On admission for each methotrexate infusion, measure:

- Serum creatinine
- Bilirubin and AST or ALT
- Plasma sodium and potassium
- FBC

Guidelines for dosing high dose methotrexate in Liver impairment

Bilirubin (micromol/L)	AST	Dose
<50	And < 180	100%
51-85	Or > 180	75%
> 85		Contraindicated

It is expected that patients receiving high dose methotrexate will develop hypertransaminasemia and occasionally hyperbilirubinemia.These elevations can last up to 2 weeks following the methotrexate infusion and are not considered toxicities requiring discontinuation of repeated administration of methotrexate. Persistant hyperbilirubinemia and/or grade 3-4 hypertransaminasemia for longer than 3 weeks should result in discontinuation of the drug. Dose reduce, particularly in patients with concomitantly impaired renal function. The drug is contraindicated in severe hepatic impairment.

Pre-hydration – For at least 6 hours prior to the commencement of the intravenous methotrexate.

Hydration fluid – 1 litre dextrose saline to which has been added 50 mmol sodium bicarbonate and 20 mmol potassium chloride.

Infusion rate – 125 ml/m^2/hour.

Check urine pH – Adjust the sodium bicarbonate concentration to maintain the urinary pH between 7 and 8 (i.e. alkaline). A urinary pH of 7.5 or greater must be achieved before starting the methotrexate infusion.
Alternating bags of sodium chloride 0.9% and glucose 5% is acceptable.

HIGH-DOSE METHOTREXATE INFUSION
Methotrexate dose
Methotrexate 3 g/m^2 with:
10% (i.e. 300 mg/m^2) given over 1 hour (loading dose) in 200 mls sodium chloride 0.9%
90% (i.e. 2700 mg/m^2) given over next 23 hours in 1 litre sodium chloride 0.9%

NOTE: The infusion of methotrexate must always stop at 24 hours even if not completed for any reason.

FOLINIC ACID RESCUE MUST START AT 36 HOURS FROM THE START OF METHOTREXATE.
The first dose of folinic acid (to be given at 36 hours after the start of methotrexate infusion) must be written up at the time of prescribing the methotrexate infusion.

Dosage of folinic acid:
At 36 hours: Give 15 mg/m^2 iv.
36-48 hours: Give 15 mg/m^2 iv every 3 hours.
From then on: Give doses as per table below until methotrexate level is less than 0.1micromol/litre.

Monitoring of plasma methotrexate levels following infusion.
Times given are from time 0 (time of starting intravenous methotrexate infusion).
The following plasma samples are **required for patient's safe rescue** with folinic acid:
48 hours, 72 hours, and then every 24 hours until methotrexate level is less than 0.1micromol/litre

Table for the calculation of folinic acid rescue on the basis of MTX plasma levels.

**Time after starting MTX**	**MTX plasma concentration micromole/litre)**				
	<0.1	0.1-2	2-20	20-100	>100

48h	None[a]	15mg/m^{2}q6h[b]	15mg/m^{2}q6h	10mg/m^{2}q3h	100mg/m^{2}q3h
72h	None	15mg/m^{2}q6h	10mg/m^{2}q3h	100mg/m^{2}q3h	1g/m^{2}q3h
96h	None	15mg/m^{2}q6h	10mg/m^{2}q3h	100mg/m^{2}q3h	1g/m^{2}q3h
120h[c]	None	15mg/m^{2}q6h	10mg/m^{2}q3h	100mg/m^{2}q3h	1g/m^{2}q3h

Notes

a No extra folinic acid is required provided MTX levels are below 0.1 micromol/litre at 48h.

b Dose and schedule of folinic acid: q6h = every 6 hours.

c At time points after 120h folinic acid administration should be continued as recommended for 120h.

Hydration regimen during and after completion of intravenous methotrexate infusion

Continue to infuse at a rate of 125 ml/m^2/hour for a minimum of 48 hours after start of methotrexate with:
1L dextrose saline containing 50 mmol of sodium bicarbonate and 20 mmol potassium chloride.
Alternating bags of sodium chloride 0.9% and glucose 5% is acceptable.
Continue to ensure that urinary pH is above 7 by adjusting sodium bicarbonate dose.

After 48 hours from the start of the intravenous methotrexate, **ENSURE** a combined oral and/or intravenous intake greater than 3 litres/m^2/24 hours until plasma methotrexate levels<0.1 micromols/litre.

Check fluid balance at regular intervals (at least 4-hourly) through each day, taking early action if fluid overload occurs by giving furosemide if the urine output falls below 400 ml/m^2 in any given 4-hour period.

Other investigations during folinic acid rescue:
Daily Creatinine, sodium and potassium.
Alternate days Bilirubin, AST, ALT, albumin, full blood count.

These investigations should also be checked at least twice during the week following the first and second methotrexate infusion to detect any toxicity that might occur.

Conversion table for methotrexate levels expressed in different units

Molar (M)	µmol/l
1×10^{-3}	1013.0
2×10^{-4}	202.0
1×10^{-4}	101.0
2×10^{-5}	20.0
1×10^{-5}	10.1
2×10^{-6}	2.0
1×10^{-6}	1.01
2×10^{-7}	0.2
1×10^{-7}	0.10

Drug interactions

Drugs which compromise renal function eg. Aminoglycosides and cisplatin can decrease clearance of methotrexate and lead to systemic toxicity. Avoid concurrent use of Non steroidal anti inflamatories **(NSAIDs) including salicylates and sulphonamides**.
Large doses of penicillin may interfere with the active renal tubular secretion of methotrexate.

It is recommended that prophylactic co-trimoxazole be stopped one week before high dose MTX therapy, until consolidation therapy starts.

Appendix 16: Karnofsky Performance Status

KARNOFSKY PERFORMANCE STATUS SCALE DEFINITIONS RATING (%) CRITERIA

Able to carry on normal activity and to work; no special care needed.	100	Normal no complaints; no evidence of disease.
	90	Able to carry on normal activity; minor signs or symptoms of disease.
	80	Normal activity with effort; some signs or symptoms of disease.
Unable to work; able to live at home and care for most personal needs; varying amount of assistance needed.	70	Cares for self; unable to carry on normal activity or to do active work.
	60	Requires occasional assistance, but is able to care for most of his personal needs.
	50	Requires considerable assistance and frequent medical care.
Unable to care for self; requires equivalent of institutional or hospital care; disease may be progressing rapidly.	40	Disabled; requires special care and assistance.
	30	Severely disabled; hospital admission is indicated although death not imminent.
	20	Very sick; hospital admission necessary; active supportive treatment necessary.
	10	Moribund; fatal processes progressing rapidly.
	0	Dead

Appendix 17: Protocol Version History

**Protocol:**		**Amendments:**		
**Version no.**	**Date**	**Amendment no.**	**Section (no./title)**	**Summary of main changes from previous version**
1.0	11.11.2009	n/a	n/a	n/a
2.0	17.05.2010	2		Change to Palifermin Dosing Schedule.<br>Guidance added for following local policy for non IMP appendix, MTX appendix.<br>Other minor administrative changes
3.0	18.08.2010	19.08.2010		Removal of Epratuzumab.<br>Other minor administrative changes.
4.0	24.04.2012	24.04.2012		Urgent Safety Measure to amend the doses of Daunorubicin and Pegylated Asparaginase.
5.0	20.07.2012	03.08.2012		The age range was amended to include patients aged between 19-24 (Philadelphia Positive patients only)<br>An additional blood sample (4.5ml) is to be collected for further analysis.<br>Other minor administrative changes throughout.
6.0	20.06.2014	20.06.2014		Addition to transplant inclusion criteria.<br>Other minor administrative changes.<br>Removal of statements relating to Rituximab coming off patent and sites being charged for supplies. Philadelphia positive patients do not receive any Pegylated Asparaginase on the trial.<br>Changes to exclusion criteria, relating to Hep B/C reactivation and Rituximab
6.1	22.01.2015	#6.1	Page 3	Change of Trial Coordinator
			5.7	Bullet point amended as follows (deleted text ~~struck through~~): "Registration to either maintenance /consolidation or transplant will be performed centrally at the UCL CTC ~~must be performed prior to commencement of any transplant activity~~."<br><br>Bullet point amended as follows (newly added text in **bold**): "A maintenance/consolidation registration form or transplant registration form must be fully completed and faxed to UCL CTC **as soon as possible after completion of phase 2 induction. This** will be used to confirm transplant eligibility at UCL CTC."<br><br>New bullet point added: "For patients aged 40 years and under at study entry and undergoing myeloablative transplant. The palifermin (P) randomisation must be performed prior to commencement of any transplant activity."
			Throughout	Correction of typographic errors

Version no.	Date	Amendment no.	Section (no./title)	Summary of main changes from previous version
7.0	16.08.2015	#7.0	pp 3-4	Administrative changes to study contacts
			1.1	- Removal of Eire from 'target countries' section; addition of New Zealand<br>- Correction of duration of follow up<br>- Updates to details of IMP suppliers<br>- Clarification of groups intended to proceed to transplant
			2.8.1, 5.6.2, 7.2.10, 7.2.10.1,	Clarification of age group intended to receive myeloablative transplant
			2.8.3, 5.6.2, 7.2.10.2, 17.3	Clarification of age group intended to receive reduced intensity (non-myeloablative) transplant
			2.8.5, 5.6.1	Additional stem cell source incorporated: cord blood now permitted for patients without a sibling or matched unrelated donor
			3.3, 10.4, 10.5, 12.4, 12.7, 13.1	Removal of references to country coordinating centres as international collaborators will not have a CCC
			4.0	Removal of references to diary cards (never used for UKALL14 trial) and informed consent log (no longer used following a change to the monitoring plan)
			5.1, 9.7	Addition of optional collection of samples for constitutional DNA – either a buccal swab at baseline or extraction of constitutional DNA from a remission bone marrow sample already stored at the MRD laboratory
			5.4, 5.7	- Removal of reference to patient-specific CRFs being sent to sites following registration – patient-specific CRFs are no longer sent to sites<br>- CTC opening hours updated to specify "excluding public holidays"
			5.5, 7.2.10.7	If site carries out multilineage chimerism locally, it is acceptable not to send additional samples to the central laboratory for chimerism testing, however the site must send all relevant local reports to UCL CTC.
			7.1	Nelarabine supplier changed from GlaxoSmithKline to Novartis
			7.2	Important notes regarding treatment with Oncaspar, Nelarabine, non-IMP backbone chemotherapy and transplant conditioning added, intended to promote compliance and ensure patient safety
			7.2.1	- Additional contact provided for haemostatic advice<br>- Clarification that in the case of methotrexate encephalopathy, IT methotrexate should be discontinued

Version no.	Date	Amendment no.	Section (no./title)	Summary of main changes from previous version
			7.2.1, 12.3	Classification of venous thromboembolisms as AEs of special interest – reporting procedures clarified
			7.3.3, 8.2.1	Timing of samples for asparaginase levels clarified:<br>Age 40 & under: d3/4 & d18<br>Age 41 & over: d18 & d32
			7.2.4, 7.2.6, 8.2.1 appendix 7	Clarification of nature and timing of MRD samples
			5.3, 7.2, 7.2.5	Updated guidance given about the timing of nelarabine (following count recovery at the end of phase 2) and contraindications to nelarabine administration.
			7.2.8	Removal of mention of imatinib being continued until transplant in consolidation treatment tables (patients who have transplant will not receive consolidation)
			7.2, 7.2.9	Clarification that IT methotrexate doses should be made up if missed.
			7.2.10	Some flexibility now permitted with regard to the intensity of transplant administered. If the site does not wish to give a transplant as per protocol, this must be agreed in advance with the UKALL14 Transplant Coordinator, and transplant case report forms will be completed and sent. Follow up will be the same as for patients who had per-protocol transplants.
			7.2.10.1	- Clarification of permitted myeloablative conditioning regimens<br>- Reminder that deviations from protocol conditioning must be agreed in advance by the UKALL14 Transplant Coordinator<br>- Addition of suggested GvHD prophylaxis to treatment tables
			7.2.10.2	- Reminder that deviations from protocol conditioning must be agreed in advance by the UKALL14 Transplant Coordinator<br>- Addition of schedule for post-transplant IT Methotrexate administrations to treatment table<br>- Addition of suggested GvHD prophylaxis to treatment table<br>- Clarification of timeframe and nature of samples needed for chimerism & MRD assessments
			7.2.10.7	Clarification of timing of chimerism testing and DLI administration added
			7.2.10.8	Clarification of reporting timeframe for chimerism samples added
			7.2.10.9	Change of recommended T-cell depletion drug from the Campath-1-H brand to the generic alemtuzumab.

Version no.	Date	Amendment no.	Section (no./title)	Summary of main changes from previous version
			7.2.11	Clarification of timing of intrathecal methotrexate after transplant for RIC allograft patients
			8.1	Schedule of assessments changed from free text to table to improve clarity
			8.3	Annual follow-up to continue until end of study or until death.
			9.0, Appendix 7	Change of central laboratory details from Royal Free to UCL Cancer Institute following a recent move
			10.0; 10.4	Data management section updated in line with current UCL CTC protocol template
			12.2.2	Amendment of SAE reporting window for T-cell patients who do not receive Nelarabine;<br>Expansion of SAE reporting timeframes table to clarify reporting windows during intensification, consolidation and post-transplant
			12.2.2.5	Clarification of CTC's responsibilities with regard to reporting SAEs & SARs to drug suppliers
			12.6.3	Clarification of CTC's responsibilities with regard to reporting pregnanices to drug suppliers
			14.2	Clarification of CTC's policy regarding withdrawal of consent for data collection
			14.3	Process for transferring care to another participating site added
			15.1	Removal of non-interventional long term follow-up phase; data collection will now cease at declaration of end of trial
			15.2	Archiving section updated in line with current UCL CTC protocol template
			15.4	Site withdrawal section updated in line with current UCL CTC protocol template
			16.1.1	Removal of requirement for central monitoring of informed consent logs. Following a recent change to the monitoring plan, the informed consent process is now monitored 'in real time' via the randomisation case report form
			17.2, 17.3, 17.4, 17.5, 17.6	Clarifications and corrections to statistics section
			21.0	Addition of further references
			Appendix 1	Minor corrections and additions to abbreviations list
			Appendix 2	Link to electronic medicines compendium website added for reference
			Appendix 3	Clarification that storage information provided is for guidance, and sites should store and handle non-IMP drugs as per the SmPC for the brand stocked at site

			Appendix 7	Clarification of samples required for MRD testing
			Appendix 11	Minor corrections and addition of explanatory notes to ensure consistency with main body of protocol
			Throughout	Correction of typographical errors and other minor administrative changes
8.0	12/05/2016	#8.0	1.0, 1.1, 2.8.1, 2.8.2, 2.10, 12.2.2.5, 17.3, 17.5	Text updated to clarify that palifermin randomisation was closed in April 2016 as no longer clinically relevant
			1.2, 5.2.4, 5.3, 5.7, 7.1, 7.2.10.1, 7.2.10.5, 8.1, 11.2, 12.2.2.1, appendix 2, appendix 11, appendix 13 (now deleted)	Removal of text directly detailing processes and data collection for the palifermin randomisation
			5.2.4, 12.6.5	Change of pregnancy risk window and contraceptive guidance in line with information in SPCs
			7.1, 7.2	Minor clarifications to treatment section
			12.2.1.4	Clarification of what is classified as causally related
			7.2.10, 8.2.1	Clarification that mini satellite regions sample only required for RIC transplant patients
			1.1, 3.3, 12.4, 12.6.3, 12.7, 13.1	Removal of international references as set up at international sites no longer going ahead
			12.4	SUSAR reporting procedures updated in line with current UCL CTC protocol template
			12.6, 12.6.1	Pregnancy reporting section updated in line with current UCL CTC protocol template
			1.1, 7.0, 12.2.2.5, 12.6.3, 12.7	Updated to reflect change of supplier for oncaspar from Sigma Tau to Baxalta, following company acquisition
			1.1	Optional extraction of constitutional DNA added to summary of correlative science
			Throughout	Correction of typographical errors and other minor administrative changes
9.0	17.02.2017	#9.0	Signature page, Other Trial Contacts	Administrative changes to study contacts
			1.1, 17.1	Amended to reflect extended recruitment
			1.1, 7.0	Updated in the light of recent drug company acquisitions

			7.2.10	Amended to allow patients who have no features predictive of a high risk of relapse to remain on trial if they opt not to proceed to transplant
			7.2.10, 7.2.10.2, 7.2.10.7, 7.2.10.8, 8.2.1, 9.0, Appendix 7, Appendix 11	Following the move of the MRD lab from the Royal Free Hospital to UCL Cancer Institute, it is no longer possible to send chimerism samples and MRD samples to a single address
			9.7	Clarification added to explain the method of obtaining DNA samples for new patients
			16.1.3	Updated following recent change in UCL CTC's monitoring SOPs
			21.0	Additional reference, cited in section 17.1.
			Throughout	Correction of typographical errors and other minor administrative changes
10.0<br><br>NB Not implemented at sites	24.05.2017	#10.0	1.1, 1.2, 2.10, 5.4, 7.1.3, 7.2.12, 8.2, 8.2.1, 8.2.2, 8.2.3, 11.2, 12.2.1, 12.2.2, 12.6, 15.1, 17.1, 17.3.2, 17.4	Amended to reflect the inclusion of 'registration only' patients
			5.1, 5.2, 5.2.4, 5.3, 5.4, 7.1.1, 7.2, 7.2.10, 7.2.10.2, 7.2.10.7, 8.1, 8.1.2, 9.6, 12.0, 12.7, 16.1.1, Appendix 7, Appendix 11	Amended to distinguish between randomised patients and 'registration only' patients
			1.1, 2.6, 7.2.3, 7.2.7, 8.1.2, 9.2.1, 9.2.2, 17.3, Appendix 2, Appendix 4	Amended to standardise drug name throughout document
			4.0	Clarification regarding time for consenting when 24 hours is not possible
			5.2.2	Clarification regarding mandatory hepatitis testing
			7.1.2	Confirmation added regarding the non-IMPs for the purpose of the trial
			7.2.1	Clarification added regarding imatinib and azoles interaction
			8.1.1	Assessments added which had been missed in error
			8.1.2	Clarification added

			9.0	Clarification added regarding stored samples
			9.7	Clarification added regarding constitutional DNA sample consent
			9.7, Appendix 7	Updated contact names
			10.1	Amended to reflect current UCL CTC SOP
			11.1, 11.3	Added in line with current UCL CTC Protocol template
			17.3.2	Confirmation added that patients treated prior to the USM will be excluded from primary analyses
			Appendix 3	Clarification added to confirm that generic brand non-IMPs can be used
			Throughout	Correction of typographical errors and other minor administrative changes
11.0	11.09.2017	#11.0	1.1, 1.2, 2.10, 5.2, 5.3, 5.4 (section now deleted), 7.1, 7.2,8.2, 9.6, 11.2, 12.0, 12.2, 12.6, 17.1, 17.3.2, 17.4	Removal of 'registration only' pathway from within main protocol
			2.10, Appendix 1	Addition of 'registration only' sub-study
			5.3.2, 7.2.10, 8.1.2, 9.0, 9.4.2, Appendix 8, Appendix 12	Removal of Chimerism lab. Sites will now perform chimerism locally, taking samples as per local standards and send UCL CTC anonymised reports
			5.3.1, 5.3.4	Guidance on patient identifiers must be redacted if a registration form needs to be sent by email
			7.0, 11.0	References to Palifermin were previously removed in error therefore this has been listed again
			Throughout	Amendment of appendix numbering
			Throughout	Amended to standardise RIC transplant as 'non-myeloablative'
12.0	26.06.2018	#12.0	1.1, 17.0	Update of recruitment target to account for analysis exclusions
			2.10, Appendix 1	Addition of T-cell patients to 'registration only' sub-study
			5.2.4	Update to contraceptive advice to reflect updated Oncaspar SPC
			7.2.1	Clarification added to suggest that sites do not need to use hydrocortisone specifically as steroid cover for IT cytarabine
			7.2.5, 7.2.6, Appendix 12	Clarification added regarding timing of post-phase 2 MRD sample and nelarabine administration

			Appendix 1	Recruitment to 'registration only' sub-study extended
			Appendix 8	Clarification added to match MRD fees to the national rate
			Throughout	Correction of typographical errors and other minor administrative changes
13.0	16.07.2021	#13	TMG	Update to TMG members
			Contents page	Administrative changes
			Appendix 1	Recruitment to 'registration only' sub-study extended and adjustment of projected recruitment numbers.
14.0	13.10.2021	#14	Appendix 1	- Update to end of recruitment date for the 'registration only' sub-study and adjustment of projected recruitment numbers.
			Appendix 8	- An update to end date for baseline samples to be sent to the ALL MRD lab.
